# MEK inhibitors overcome resistance to BET inhibition across a number of solid and hematologic cancers

**DOI:** 10.1038/s41389-018-0043-9

**Published:** 2018-04-20

**Authors:** Anastasia Wyce, Jeanne J. Matteo, Shawn W. Foley, Daniel J. Felitsky, Satyajit R. Rajapurkar, Xi-Ping Zhang, Melissa C. Musso, Susan Korenchuk, Natalie O. Karpinich, Kathryn M. Keenan, Melissa Stern, Lijoy K. Mathew, Charles F. McHugh, Michael T. McCabe, Peter J. Tummino, Ryan G. Kruger, Christopher Carpenter, Olena Barbash

**Affiliations:** 1 Cancer Epigenetics DPU, Oncology R&D, GlaxoSmithKline, Collegeville, PA USA; 2 Target Sciences, GlaxoSmithKline, Upper Merion, PA USA; 3 Present Address: Janssen Pharmaceuticals, Spring House, Montgomery, PA USA

## Abstract

BET inhibitors exhibit broad activity in cancer models, making predictive biomarkers challenging to define. Here we investigate the biomarkers of activity of the clinical BET inhibitor GSK525762 (I-BET; I-BET762) across cancer cell lines and demonstrate that KRAS mutations are novel resistance biomarkers. This finding led us to combine BET with RAS pathway inhibition using MEK inhibitors to overcome resistance, which resulted in synergistic effects on growth and survival in RAS pathway mutant models as well as a subset of cell lines lacking RAS pathway mutations. GSK525762 treatment up-regulated p-ERK1/2 levels in both RAS pathway wild-type and mutant cell lines, suggesting that MEK/ERK pathway activation may also be a mechanism of adaptive BET inhibitor resistance. Importantly, gene expression studies demonstrated that the BET/MEK combination uniquely sustains down-regulation of genes associated with mitosis, leading to prolonged growth arrest that is not observed with either single agent therapy. These studies highlight a potential to enhance the clinical benefit of BET and MEK inhibitors and provide a strong rationale for clinical evaluation of BET/MEK combination therapies in cancer.

## Introduction

BET proteins (BRD2, BRD3, BRD4, and BRDT) modulate expression of genes involved in cell growth and oncogenesis by binding to acetylated chromatin via their bromodomains, which in turn recruit downstream effectors that promote transcription. Selective BET inhibitors, such as I-BET151 and the clinical molecule GSK525762 (I-BET762; I-BET)^1,2^, abrogate binding of BET proteins to acetylated chromatin thereby inhibiting BET-dependent transcription^1,2^. BET inhibitors exhibit broad anti-proliferative activity in cancer models^3^. Although several mechanisms have been implicated in the efficacy of BET inhibitors including transcriptional suppression of oncogenes^3^, there is no consensus and it is likely that mechanisms vary, thus making the identification of predictive biomarkers difficult.

Although BET inhibitors show broad activity in many cancer types, within each there are resistant models. Understanding the basis of BET inhibitor sensitivity and resistance is important to inform the clinical development of BET inhibitors as monotherapies and to identify rational combinations. To this end, we analyzed genetic data from a large set of cell lines treated with GSK525762 to identify biomarkers of sensitivity and resistance. From these studies, we identified KRAS mutations as a significant predictor of resistance to BET inhibition. This led us to hypothesize that combinations with inhibitors of RAS signaling, such as MEK inhibitors, may further improve upon BET inhibitor efficacy. Indeed, we observed broad synergistic effects for BET/MEK combinations across cancer models, which we attribute to profound and sustained inhibition of MEK/ERK signaling that is specifically observed with the combination ultimately leading to growth arrest and cell death.

## Results

### RAS mutations are novel biomarkers of resistance to GSK525762

**Fig. 1:**
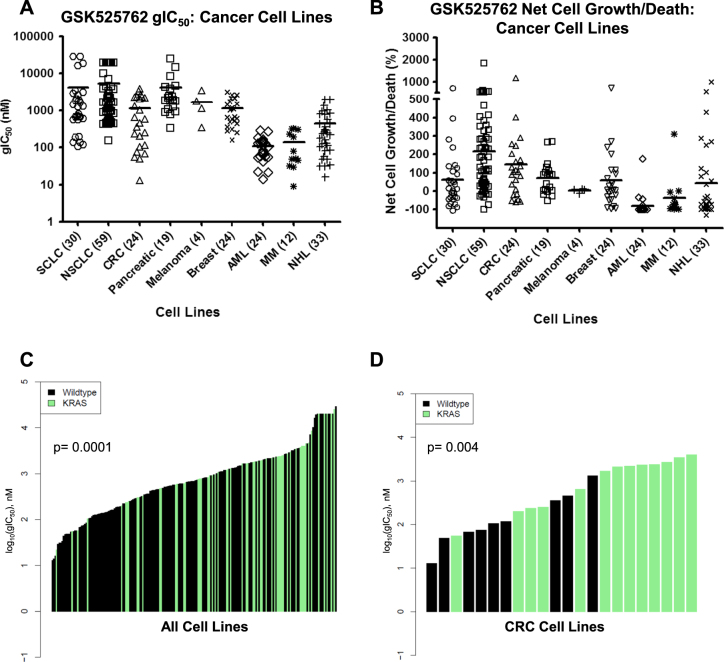
Reduced sensitivity to BET inhibition in RAS mutant cancer models. **a** gIC_50_s for GSK525762 in a panel of cancer cell lines from a 6 day proliferation assay. Mean gIC_50_ for individual tumor types indicated with a black bar. **b** Net cell growth or death of individual cancer cell lines (represented as a percent of a T0 measurement) following exposure to GSK525762 for 6 days. Mean net cell growth/death values for individual tumor types indicated with a black bar. **c** Association of RAS mutation status (black bars—WT; green bars—mutant) with GSK525762 gIC_50_ in cancer cell lines representing the tumor types detailed in **a**. Mutation data from CCLE^21^ was used for this analysis, and only cell lines with mutation data available in CCLE were included. Indicated *p*-value based on Wilcoxon rank sum test. **d** Association of RAS mutation status with GSK525762 gIC_50_ in colon cancer cell lines, as described in **c**

To identify genetic predictors of sensitivity or resistance to BET inhibitors we first examined the anti-proliferative activity of GSK525762 in ~230 cancer cell lines. Hematologic cancer cell lines were highly sensitive (low growth IC_50_ values and net cell death) to GSK525762, whereas solid tumor models exhibited a wide range in sensitivity (gIC_50_ 13 nM to > 29.3 µM; partial, cytostatic, and cytotoxic responses), providing an opportunity to compare the genetic profiles of a large number of sensitive and resistant lines (Fig. 1a, b, Supplemental Table S1). Using publicly available data for 19,312 genes with protein-changing mutations, we performed unbiased analyses of genetic predictors of sensitivity or resistance to GSK525762 based on gIC_50_ values. These analyses identified 634 genes with protein-changing mutations that correspond to resistance or sensitivity to GSK525762 (Wilcoxon rank sum test *p* < 0.05; Supplemental Table S2, Supplemental Figure S1). Notably, *KRAS* mutations were among the top five most significant (*p* = 0.0001) associations, with a clear enrichment of *KRAS* mutations among the more resistant cell lines (median gIC_50_ *KRAS* mutant = 1667 nM vs WT = 550 nM; Fig. 1c). Analysis of mutations at the amino acid level further identified *KRAS* G12 missense mutations as significantly (*p* = 0.00006) associated with resistance (Supplemental Figure S2). A similar association (*p* = 0.004) between *KRAS* mutations and resistance was observed in colorectal cancer (CRC) cell lines, where *KRAS* mutations are frequent (mutations in 13/22 lines; 59%) (Fig. 1d), indicating that the association with resistance is not driven by the general responsiveness of individual tumor types. Finally, *KRAS* mutations were significantly depleted (*p* = 0.003; Fisher’s exact test) among the cell lines exhibiting net cell death in response to GSK525762 (Supplemental Table S1), suggesting that activated RAS signaling may be a mechanism by which cancer cells are able to survive BET inhibition.

### Broad synergy for combinations of BET and MEK inhibitors in RAS pathway mutant cancer cell lines

**Fig. 2:**
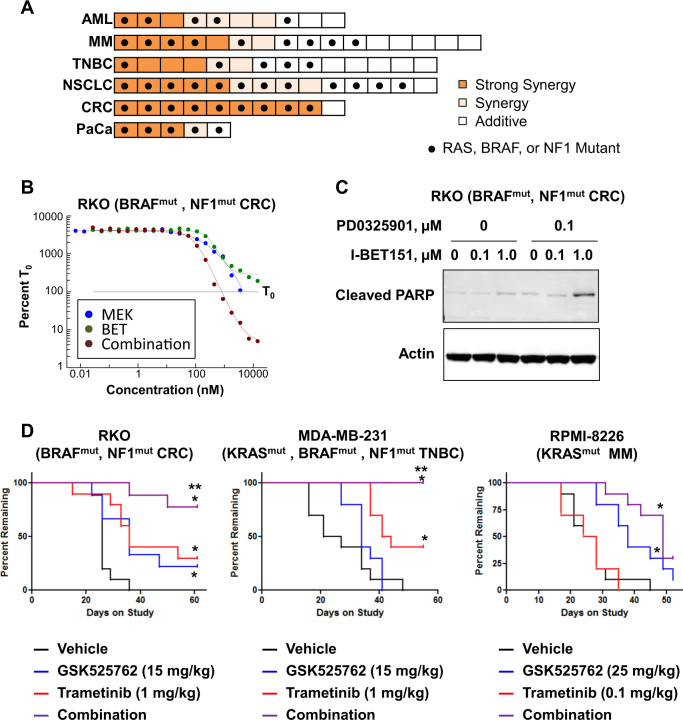
Broad synergy for the combination of BET and MEK inhibitors across solid and hematologic cancer cell lines. **a** Heat map of cancer cell line response to a combination of I-BET151 with the MEK inhibitor PD0325901 in a 3 or 6 day proliferation assay. Dark orange, light orange, and white bars reflect strongly synergistic, synergistic, and additive effects, respectively (see Materials and Methods). Black circles indicate cell lines possessing mutations in RAS, BRAF, or NF1 genes based on CCLE data. **b** Growth curves for RKO cells treated with I-BET151 and/or PD0325901 for 6 days. Data are presented as a percentage of cells present at the time of compound dosing (T_0_), which is set to 100%. Data presented is from a single experiment representative of typical results (biological *n* ≥ 2). **c** Western blot of cleaved PARP in RKO cells following a 3 day treatment with the indicated compounds. Cells were treated with 100 nM or 1 µM I-BET151, alone or in combination with 100 nM PD0325901. **d** Kaplan–Meier curves for GSK525762 and trametinib, dosed once daily as single agents or in combination in the cell line xenograft models RKO (CRC), MDA-MB-231 (TNBC), or RPMI-8226 (MM). For the RKO and MDA-MB-231 studies, GSK525762 was dosed at 15 mg/kg and trametinib was dosed at 1 mg/kg. For RPMI-8226, GSK525762 was dosed at 25 mg/kg and trametinib was dosed at 0.1 mg/kg. Single asterisk (*) indicates significant difference from vehicle (*p* < 0.05). Double asterisk (**) indicates significant difference from single agent efficacy (*p* < 0.05)

The strong association between KRAS mutations and resistance to GSK525762 suggested that agents targeting RAS signaling could sensitize cells to BET inhibitors. To test this hypothesis, we probed the activity of BET/MEK inhibitor combinations in cancer cell lines with and without RAS pathway mutations (mutations in RAS, BRAF, or NF1), and with varying levels of sensitivity to GSK525762. For these studies, we utilized the BET inhibitor I-BET151, (pharmacological profile highly similar to GSK525762^1^; Supplemental Figure S3), and the clinical MEK inhibitor PD0325901. Combination of I-BET151 and PD0325901 produced synergistic effects (CI < 0.78; see Methods) most commonly in, but not limited to, RAS pathway mutant models. Synergy was observed in multiple tumor types (Fig. 2a, Supplemental Table S3), with the combination improving growth inhibition (gIC_50_, gIC_100_) and/or cytotoxicity (dEC_50_) even in cell lines sensitive to single agent BET inhibition (Fig. 2b, c, Supplemental Table S1, S3). Across all cell lines evaluated, there was a significant correlation (*p* = 0.022; Fisher’s exact test) between RAS pathway mutations and synergy (Fig. 2a; Supplemental Table S3). However, synergy was not restricted to mutant models, suggesting that additional mechanisms may drive combination effects.

Given the reported variability in biological activity of MEK inhibitors based on differing mechanisms of inhibition^4^, we evaluated combinations with additional MEK inhibitors in cell lines that exhibited synergistic responses to I-BET151 and PD0325901. Synergy was observed with a variety of structurally distinct MEK inhibitors, including GDC-0623, trametinib, and cobimetinib (Supplemental Figs. S4A-4B). Importantly, combination of the clinical BET inhibitor GSK525762 with therapeutic concentrations of trametinib^5^ resulted in decreased cell viability (Supplemental Figure S4C).

To determine if in vitro synergy translates into improved in vivo efficacy, we profiled the activity of the BET/MEK combination in xenografts representing various tumor types and RAS pathway mutations. Combination of GSK525762 and trametinib significantly delayed tumor growth in xenograft models of CRC (RKO; BRAF^mut^, NF1^mut^), triple-negative breast cancer (TNBC; MDA-MB-231; KRAS^mut^, BRAF^mut^, NF1^mut^), and multiple myeloma (MM; RPMI-8226; KRAS^mut^) (Fig. 2d; Supplemental Figures S5-S7), and significantly improved tumor growth inhibition in a xenograft model of pancreatic cancer (PaCa; HPAF-II; KRAS^mut^) (Supplemental Figure S8) at well-tolerated doses (Supplemental Figure S9).

### Synergy for BET/MEK combinations in RAS pathway wild type cell lines

Although RAS pathway mutations predict resistance to BET inhibitors and sensitivity to BET/MEK combinations, we also observed synergy in cell lines lacking these mutations (Fig. 2a). To understand the basis of synergy in RAS pathway wild type (WT) cells we evaluated the combination in ER+breast cancer and small cell lung cancer (SCLC) cell lines where the frequency of RAS pathway mutations is low. Although no synergy was observed in ER+breast cancer models (Supplemental Table S3), we observed synergistic effects for the combination in 50% (2/4) of the SCLC cell lines evaluated (Supplemental Figure S10A). Combined BET and MEK inhibition in SCLC cell lines resulted in synergistic growth inhibition and cytotoxicity, even in cell lines (NCI-H1092) that are completely resistant to the monotherapies (Supplemental Figure S10A-B). Synergy was also observed in a subset of SCLC patient-derived xenografts treated with GSK525762 and PD0326901 ex vivo in a colony formation assay (Supplemental Figure S10C-D).

One of the two SCLC cell lines sensitive to the BET/MEK combination, NCI-H510, is NF1 mutant (D2184G) and exhibits high-basal levels of phosphorylated ERK1/2 (p-ERK1/2; Supplemental Figure S10E). NCI-H1092 cells, which are completely resistant to GSK525762 monotherapy but sensitive to the BET/MEK combination, lack RAS pathway mutations but also possess high levels of p-ERK1/2. In contrast, the SCLC cell lines exhibiting additive effects to the combination lacked RAS pathway mutations, and exhibited low levels of p-ERK1/2. Taken together with our observations in other tumor types, these data suggest that activated MEK/ERK signaling, either from RAS pathway mutations or other causes, is likely a driver of resistance to BET inhibition and sensitivity to combinations with MEK inhibitors.

### BET/MEK combinations uniquely sustain down-regulated expression of genes required for cell cycle progression

To explore the mechanisms driving synergy between BET and MEK inhibition we first evaluated gene expression changes in the CRC cell line RKO (BRAF V600E; NF1 V2205A) following treatment with GSK525762, trametinib, and the combination (Supplemental Figure S11A-B). We observed gene expression changes following GSK525762 and trametinib single agent treatment that were consistent with previous reports, including down-regulation of MYC and KRAS signatures, respectively (Supplemental Table S4). Combination treatment for 24 or 96 h resulted in greater numbers of gene expression changes in RKO cells compared to either single agent, and included the majority of gene expression changes observed with monotherapy (Supplemental Figure S11C-D) at each time point. At the gene signature level, these expression changes with combination treatment reflected sustained down-regulation of the same signatures observed with single agent treatment (ex: MYC, KRAS; Supplemental Table S4). In addition, combination treatment resulted in more widespread and significant down-regulation of E2F signatures and up-regulation of apoptotic signatures compared to monotherapy, consistent with the synergistic growth inhibition and cytotoxicity observed in our proliferation assays (Fig. 2a–c).

**Fig. 3:**
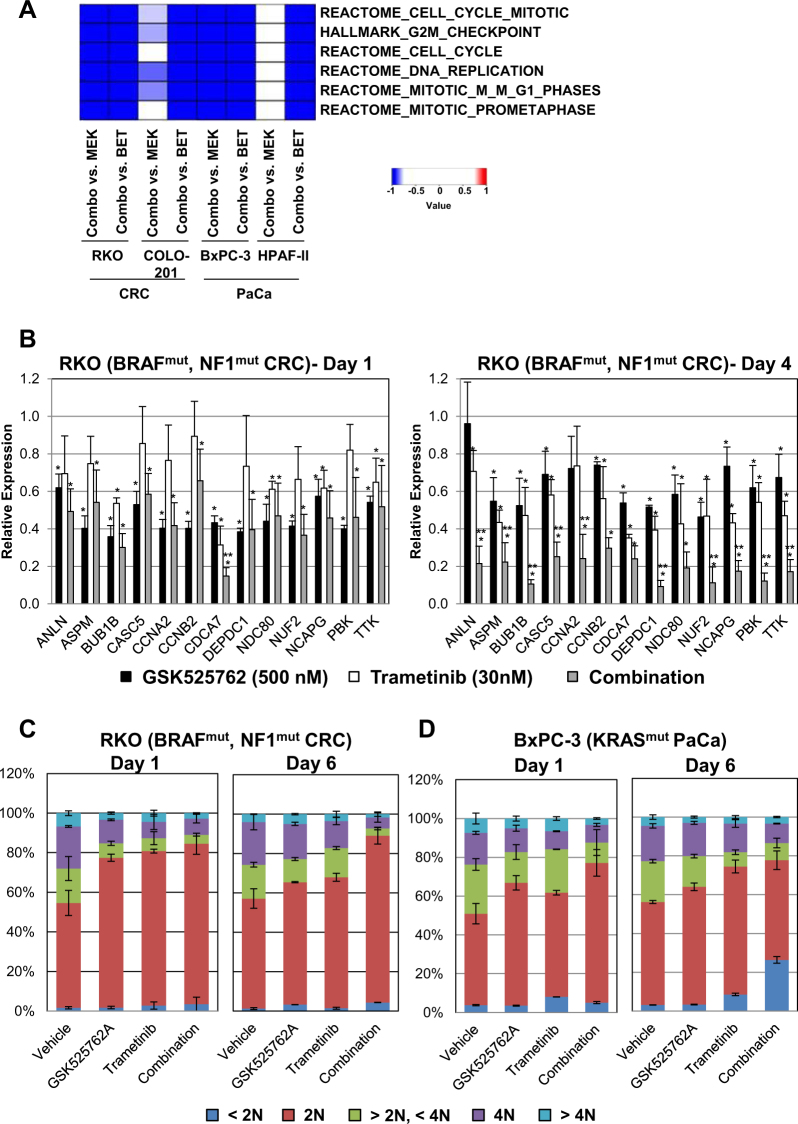
Profound and persistent effects on the cell cycle are specifically induced by combination of BET and MEK inhibition across cancer cell lines. **a** Heat map depicting gene set enrichment in the indicated cell lines following 96 h exposure to a combination of GSK525762 (500 nM) and trametinib (3 nM for COLO 201; 10 nM for BxPC-3, HPAF-II; 30 nM for RKO) compared to either GSK525762 (BET) or trametinib (MEK) single agent treatment. Heatmap is color coded based on signed (1-FDR) derived from individual GSEA analyses, where a negative value indicates down-regulation in combination-treated samples and a positive value indicates up-regulation. **b** qPCR analysis of the indicated genes in RKO cells following 24 or 96 h treatment with GSK525762 (500 nM) and/or trametinib (30 nM). Gene specific data were normalized to GAPDH expression, and are presented as average relative expression compared to DMSO controls. Error bars indicate standard deviation among biological replicates (*n* ≥ 3). Single asterisk indicates *p* ≤ 0.05 for comparison to DMSO controls (two-tailed paired Student’s *t*-test). Double asterisk indicates *p* ≤ 0.05 for comparison of combination to both GSK525762 and trametinib single agent treatments (two-tailed paired Student’s *t*-test). **c** Stacked bar graphs representing the average population of cells in various phases of the cell cycle following treatment with GSK525762 (500 nM) and/or trametinib (30 nM) for one or 6 days in the RKO cell line (biological *n* = 2). Standard deviation is indicated. **d** Stacked bar graphs representing the average population of cells in various phases of the cell cycle in the BxPC-3 cell line, as described in (**c**)

Analysis of the most robust (log_2_FC > 2 or < -2) gene expression changes unique to the combination revealed highly significant overlap between down-regulated genes and MSigDB signatures associated with mitosis (Supplemental Tables S5-S6) at both time points, with a more significant overlap occurring at 96 h. GSEA across a larger panel of CRC and PaCa cell lines treated with the BET/MEK combination revealed significant enrichment of these signatures in all cell lines evaluated (Supplemental Figure S11E; Supplemental Table S7), and in a majority of cell lines this enrichment was specific to or enhanced by the combination (Fig. 3a). Evaluation of a subset of these mitotic signature genes by qPCR in RKO cells revealed modest down-regulation with all treatments at 24 h (Fig. 3b). At 96 h, down-regulation was diminished in cells treated with GSK525762, whereas marginally improved down-regulation was observed with trametinib. In contrast, robust down-regulation of these genes was observed in combination-treated cells. Further confirmation of these time-dependent effects at the protein level were observed in RKO cells treated with single agent or combination therapy for 1 or 6 days (Supplemental Figure S12). Consistent with these in vitro effects, we observed significant down-regulation of a subset of these genes in combination-treated RKO xenograft tumors collected at the end of the efficacy study, whereas no significant effects were observed with either single agent therapy (Supplemental Figure S13). These data suggest that combined BET and MEK inhibition results in more profound and/or sustained growth arrest than can be achieved with either single agent therapy. Evaluation of cell cycle defects following single agent or combination treatment confirmed this hypothesis. In RKO cells, while we observed substantial G1 arrest at early time points in cells treated with BET or MEK inhibitors, by 6 days post-treatment growth arrest was diminished (Fig. 3c). In contrast, BET/MEK combination treatment resulted in sustained G1 arrest, with a greater proportion of cells arrested in G1 at 6 days compared to earlier time points. In BxPC-3 cells, more profound G1 arrest was observed with the combination compared to single-agent treatments following 1 day of treatment (Fig. 3d). After 6 days of treatment, cells treated with single agent BET or MEK inhibitors still exhibited modest G1 arrest, whereas substantial sub-G1 accumulation was observed with combination treatment indicative of cell death. In total, these data suggest that BET/MEK combination efficacy is driven by sustained inhibition of transcription signatures associated with cell cycle regulation that ultimately leads to G1 arrest and/or cell death.

### Sustained inhibition of MEK/ERK signaling by the BET/MEK combination

To explore the mechanistic consequences of the combination on MEK/ERK signaling compared to monotherapy, we evaluated the changes in p-ERK1/2 levels across a number of cell lines (RKO (BRAF^V600E^, NF1^V2205A^; CRC), HPAF-II (KRAS^G12D^; PaCa), (RPMI-8226 (KRAS^G12A^; MM), NCI-H510 (NF1^D2184G^; SCLC)). These cell lines represent multiple tumor types and mutations, and exhibit variable responses to the single agent therapies, thus allowing us to survey mechanisms promoting synergy across a wide variety of contexts.

Based on our cell cycle analysis (Fig. 3c), growth effects in RKO cells following single agent trametinib treatment appeared to diminish between 1 and 6 days, suggesting that cells were able to adapt to MEK inhibition over time. Consistent with this hypothesis, we observed reduced potency for single-agent trametinib on p-ERK1/2 inhibition over time in RKO (Supplemental Figure S14). Strikingly, combination of GSK525762 with trametinib in RKO cells attenuated this reactivation of ERK1/2. Similar effects were observed when p-ERK1/2 levels were evaluated by IHC in RKO xenografts in vivo. Pharmacodynamic evaluation of p-ERK1/2 levels after 7 days of treatment in RKO tumors revealed comparable down-regulation in trametinib and combination-treated animals, whereas minimal effects on p-ERK1/2 were observed with GSK525762 (Supplemental Figure S15). In contrast, tumors collected at the efficacy end point (tumor volume > 2000 mm^3^) revealed greater inhibition of p-ERK1/2 in tumors from combination-treated RKO xenografts compared to vehicle, GSK525762, or trametinib monotherapy (Supplemental Figure S16).

In contrast to RKO results, we observed minimal potency shifts for trametinib in other RAS pathway mutant cell lines sensitive to the BET/MEK combination, such as the PaCa cell line HPAF-II (Supplemental Figure S17), and co-treatment with GSK525762 did not further reduce p-ERK1/2 compared to trametinib treatment in these cells. Thus, reversal of adaptive resistance to MEK inhibition likely contributes to the synergy observed for BET/MEK combinations in a subset of RAS pathway mutant models (ex: RKO), but it is not the primary mechanism in all cell lines.

**Fig. 4:**
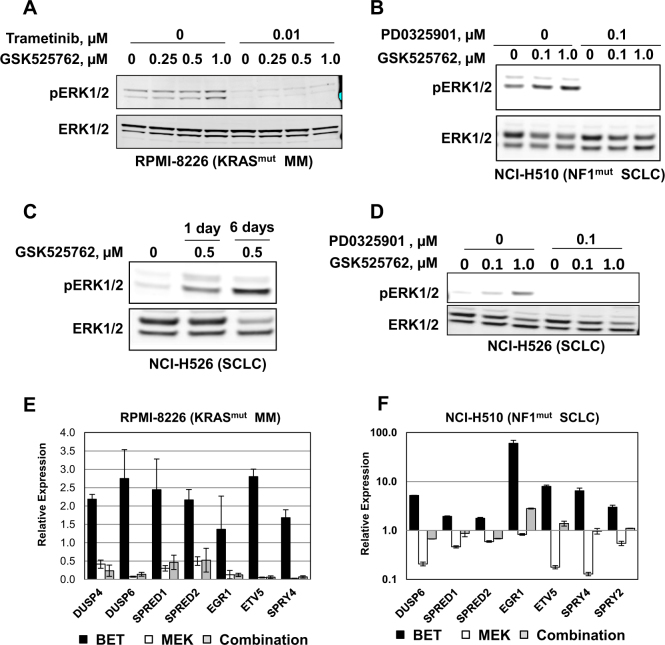
Adaptive activation of MEK/ERK signaling following BET single agent treatment is reversed by the BET/MEK combination. **a** p-ERK1/2 levels in the RPMI-8226 cell line following 4 days exposure to GSK525762 (250 nM, 500 nM, 1 µM), trametinib (10 nM), or a combination of the two agents. **b** p-ERK1/2 levels in the SCLC cell line NCI-H510 following 3 days exposure to GSK525762 (100 nM, 1 µM), PD0325901 (100 nM), or a combination of the two agents. **c** Western analysis of p-ERK1/2 levels in the SCLC cell line NCI-H526 following 1 or 6 days exposure to GSK525762 (500 nM). **d** Western analysis of p-ERK1/2 levels in the SCLC cell line NCI-H526 following 3 days exposure to GSK525762 (100 nM or 1 µM), PD0325901 (100 nM), or a combination of the two agents. **e** qPCR analysis of the indicated genes in RPMI-8226 cells following 96 h treatment with GSK525762 (500 nM) and/or trametinib (10 nM). Gene specific data were normalized to GAPDH expression, and presented as relative expression compared to DMSO controls. Standard deviation is indicated (biological *n* = 2). **f** qPCR analysis of the indicated genes in NCI-H510 cells following 96 h treatment with GSK525762 (1 µM) and/or PD0325901 (100 nM). Gene specific data were normalized to GAPDH expression, and presented as relative expression compared to DMSO controls. Standard deviation is indicated (biological *n* = 2)

We next explored the effects of the combination in RAS pathway mutant models that are intrinsically resistant to MEK inhibition, such as the MM cell line RPMI-8226 and the SCLC cell line NCI-H510 (Fig. 2d, Supplemental Figure S10A). While our single agent screen of GSK525762 revealed MM as a highly sensitive tumor type (Supplemental Table S1), the KRAS mutant cell line RPMI-8226 is one of the least responsive MM models to BET single agent treatment. Intriguingly, we observed that treatment of RPMI-8226 cells with GSK525762 for 4 days resulted in increased levels of p-ERK1/2 (Fig. 4a). Similar up-regulation of p-ERK1/2 levels were observed with the BET inhibitors I-BET151 and JQ-1 (Supplemental Figure S18), suggesting this is a general effect of BET inhibition in RPMI-8226. Up-regulated p-ERK1/2 was also observed in the SCLC cell line NCI-H510 (Fig. 4b), suggesting that synergy results from these cell lines becoming more dependent on MEK/ERK signaling as a result of adaptive pathway activation following GSK525762 treatment. Similar up-regulation of p-ERK1/2 was observed as early as 24 h post-treatment with GSK525762 in NCI-H526 SCLC cells that lack mutations in the RAS pathway (Fig. 4c, d), indicating that this mechanism is likely relevant in both the RAS pathway mutant and WT setting. Importantly, up-regulation of p-ERK1/2 in these cell lines was reversed by co-treatment with MEK inhibitors (Fig. 4a, b, d).

Consistent with the p-ERK1/2 effects, we observed time-dependent up-regulation of genes associated with ERK activation^6^ following GSK525762 treatment in NCI-H510 cells (Supplemental Figure S19A). Up-regulation of these genes was also observed following GSK525762 treatment in RPMI-8226 cells, but not in RAS pathway mutant PaCa or CRC cell lines (Supplemental Figure S19B). Combinations with MEK inhibitors in RPMI-8226 and NCI-H510 led to down-regulation of these genes compared to GSK525762 treatment (Fig. 4e, f). In total, these data suggest that activated MEK/ERK signaling is a mechanism of adaptive resistance to BET inhibition, thus providing a potential explanation for the broad synergy of BET/MEK combinations in RAS pathway mutant and WT cell lines that are resistant to MEK single agent therapy.

Recently, increased p-ERK1/2 levels were reported in ovarian cancer models rendered resistant to BET inhibition, which was suggested to result from transcriptional up-regulation of receptor tyrosine kinases (RTKs) that activate MEK/ERK signaling^7^. To explore this as a potential mechanism accounting for ERK activation in our studies, we profiled gene expression changes by microarray in the SCLC cell line NCI-H510 following 24 or 96 h of treatment with GSK525762. Consistent with our qPCR studies, we observe robust up-regulation of genes associated with ERK activation^6^ (Supplemental Table S8). In addition, we observe modest (FC < 2) but significant (*q* < 0.05) up-regulation of *FGFR2*, *FGFR3*, and *FGFR4*. Up-regulation of EGR1 and FGFR4 protein in NCI-H510 cells following GSK525762 treatment was confirmed by Western blot (Supplemental Figure S20). Taken together, these data suggest that adaptive RTK activation may account for the GSK525762-mediated induction of MEK/ERK signaling in this model.

## Discussion

**Fig. 5:**
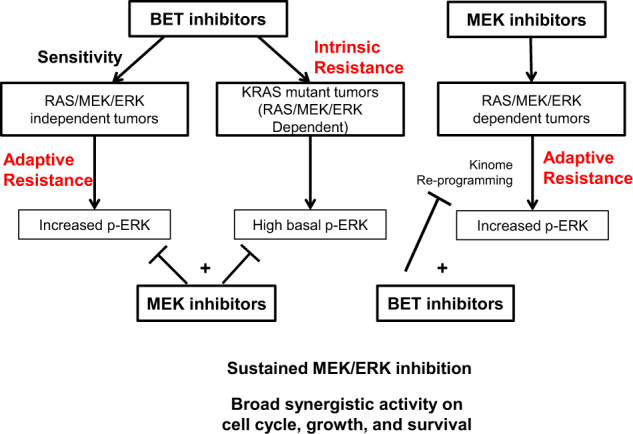
Mechanisms driving sensitivity to BET/MEK combinations in various contexts. Intrinsic and acquired resistance to BET inhibition is mediated by activation of ERK signaling, which can be reversed by co-treatment with MEK inhibitors. In specific RAS pathway-dependent models, acquired resistance to MEK inhibitors is mediated by kinome reprogramming, which can be reversed by co-treatment with BET inhibitors. In all scenarios, combined BET/MEK inhibitor treatment sustains inhibition of MEK/ERK signaling, leading to sustained growth arrest and synergistic effects on proliferation and cell death

Although previous literature has suggested potential utility of BET/MEK combinations in a few, specific settings^6–11^, our data show that combined BET and MEK inhibition is broadly synergistic across solid and hematologic cancer models. This broad synergy includes a few surprising settings, such as TNBC and SCLC, where RAS pathway mutations are infrequent but activated RAS signaling is observed^12,13^. We further show that the mechanisms driving sensitivity to the combination are context dependent (Fig. 5). Our data delineate a complex interaction between BET proteins and RAS signaling in which BET inhibition can either activate or inhibit ERK, depending on the context. However, in all cases the combination of BET and MEK inhibitors results in sustained inhibition of MEK/ERK signaling, which parallels the more profound and persistent growth arrest observed for the combination compared to monotherapy.

We demonstrate that combined BET/MEK inhibition reverses resistance observed with the single agent therapies in specific settings. First, we show that KRAS mutations are significantly associated with intrinsic resistance to GSK525762 (Fig. 1c, d; Supplemental Figures S1-S2). Although BET inhibitors transcriptionally down-regulate a number of oncogenic pathways^3^, these data suggest that BET inhibition is not sufficient to counteract the pro-growth/survival effects of activated RAS signaling in the KRAS mutant setting. Our findings provide a potential biomarker which can further refine patient populations likely to benefit from BET inhibitors, however one important question for further investigation is whether MAPK signaling activity, in comparison to KRAS mutations, is a better predictor of resistance. Further, while the combination of GSK525762 with MEK inhibition resulted in synergistic effects in many KRAS mutant models, there are exceptions in which the combination produced additive effects (Supplemental Table S3). It is possible that KRAS primarily signals through other effector pathways in these models, and therefore combinations between inhibitors of BET proteins and other RAS effector pathways should be explored.

We provide evidence that up-regulation of MEK/ERK signaling is a mechanism of adaptive resistance to BET inhibition in specific settings (e.g., MM, SCLC). This mechanism is observed in cell lines with variable responses to GSK525762, but that are intrinsically resistant to MEK inhibition irrespective of RAS pathway mutation status. Up-regulated RTK expression and p-ERK1/2 levels were recently reported in ovarian cancer models rendered resistant to the BET inhibitor JQ-1, resulting in sensitization to combinations with trametinib^7^. In addition, RNA-sequencing in a BET-resistant TNBC model revealed up-regulation of *FGFR1*, *SPRY2*, *SPRY4*, and *SPRED2* compared to parent cells, suggesting that activated RAS/MEK/ERK signaling may be associated with adaptive resistance to BET inhibition in TNBC^14^. Consistent with these reports, RTK and immediate early gene up-regulation and increased p-ERK1/2 are observed in NCI-H510 cells after GSK525762 treatment (Fig. 4b, Supplemental Table S8, Supplemental Figure S20). Based on these results, we suggest that NCI-H510 cells become more dependent on MEK/ERK signaling for survival following GSK525762 treatment, therefore explaining the synergistic growth inhibition and cytotoxicity observed for BET/MEK combinations in this model (Supplemental Figure S10A-B).

Although our data suggest activated MEK/ERK signaling as a mechanism of adaptive resistance to BET inhibition in SCLC and MM models, additional pathways promote BET inhibitor resistance in other contexts. For instance, increased Wnt/β-catenin signaling and BRD4 hyper-phosphorylation due to decreased PP2A activity have been cited as resistance mechanisms in specific models of AML and TNBC, respectively^14–16^. Additional studies are warranted to further define the mechanisms leading to BET inhibitor resistance in specific tumor types, which will facilitate the formulation of rational combination strategies to reverse these effects.

We further show that BET inhibition reverses adaptive re-activation of MEK/ERK signaling triggered by trametinib treatment in a MEK-dependent cell line (RKO). Recent studies have reported BET proteins as regulators of kinome reprogramming associated with resistance to targeted kinase inhibitors (TKIs)^17,18^. Mechanistically, BRD4 chromatin occupancy is altered following treatment with TKIs, resulting in activation of survival-promoting pathways that can be reversed by co-treatment with transcriptional inhibitors^19^. Consistent with these reports, BET inhibition attenuated the loss of p-ERK1/2 inhibition induced by prolonged exposure to trametinib in RKO cells in vitro and in vivo (Supplemental Figure S14-S16). However, outside of RKO, we have observed minimal evidence of adaptive MEK resistance based on changes in p-ERK1/2 inhibition in our experiments. Thus, reversal of adaptive resistance to MEK inhibition contributes to BET/MEK synergy in specific contexts, but is not the primary mechanism driving the broad activity of the combination.

Despite the opposing effects of BET inhibition on RAS/MEK/ERK signaling in specific contexts, combined BET and MEK inhibition results in sustained inhibition of MEK/ERK signaling, as well as robust and durable inhibition of mitotic gene signatures compared to monotherapy, across all cell types evaluated. The transient vs. persistent effects on mitotic gene expression for the single agent and combination therapies, respectively, is consistent with the cell cycle effects we observe in RKO cells. We propose that effects of the combination on mitotic gene expression and cell cycle arrest are intimately linked to the sustained inhibition of ERK activity, thus explaining why the combination of BET and MEK inhibition produces the greatest impact on cell growth and survival.

Currently, GSK525762 and other BET inhibitors are being evaluated in Phase 1/2 clinical trials. MEK inhibitors have been evaluated in dozens of clinical trials as monotherapy and in combination with other agents, and to-date, have only been approved for use in combination with BRAF inhibitors in BRAF mutant melanoma (clinicaltrials.gov). Our data highlights the potential of BET/MEK combinations in a broad range of tumors, including SCLC, TNBC, and PaCa, where patients have few treatment options. The ability of BET and MEK inhibitors to complement one another in maintaining inhibition of pro-growth pathways may result in efficacy that is not achievable with monotherapy. Emerging data suggest that additional combinations between BET and targeted kinase inhibitors may also provide benefit^7,17,18^. Further evaluation of the interplay between BET inhibitors and various signaling pathways in patient samples will be crucial to understand how this pre-clinical data may translate into clinical response.

## Materials and methods

### Cell lines and reagents

Cell lines were obtained from ATCC or DSMZ, authenticated via STR profiling, tested for mycoplasma contamination, and grown in recommended medium. Details on antibodies and TaqMan probes used are in Supplemental Methods.

### Cell line growth assays

Cell line growth assays were performed, as previously described^20^. Results were plotted as percent of the Time_0_ (T_0_) measurement (normalized to 100%) vs. compound concentration, and a 4-parameter equation was used to generate concentration response curves. Growth IC_50_ (gIC_50_) values correspond to the mid-point of the growth window (between DMSO and T_0_ values). Growth IC_100_ (gIC_100_) values correspond to the concentration resulting in 100% growth inhibition. Net cell growth or death was evaluated by subtracting the T_0_ value from the minimum of the curve (Y_min_). A positive Y_min_-T_0_ value indicates net cell growth during the assay, and a negative value indicates net cell death. Death EC_50_ (dEC_50_) values correspond to the concentration at which 50% net cell death is observed. A minimum of two biological replicates were evaluated for each cell line, and average values across biological replicates are reported.

For biomarker analyses, mutation data was obtained from CCLE^21^. Statistical analyses were performed using a Wilcoxon rank sum test on log_10_ transformed gIC_50_ values, comparing median values for wild type vs. mutant cell lines. Cell lines lacking mutation data in CCLE were excluded from this analysis.

### Combination growth assays

Growth assays were performed, as described above. BET and MEK inhibitors were titrated together at fixed ratios (typically 4-BET:1-MEK or 5-BET:1-MEK). Cells were treated with single agent or combination compound titrations for 3 or 6 days. Concentration response curves were generated for each single agent and combination treatment, as described above. Synergy was assessed by calculating combination indices (CI)^22^, assuming mutually non-exclusive effects. Combinations were deemed strongly synergistic if CI values were <0.44 and there was a minimum 5-fold shift in potency from each single agent curve in any single measured parameter (gIC_50_, gIC_100_, dEC_50_). Combinations were deemed synergistic if CI values were <0.78 and there was a minimum of a 3-fold shift in potency from each single agent curve in any single measured parameter. CI values between 0.78 and 1 were considered additive, as well as CI values <0.78 where the fold shift in potency from one of the single agents was < 3-fold. Statistical analyses were performed in GraphPad Prism using Fisher’s exact test.

### In vivo studies

All studies were conducted in accordance with the GSK Policy on the Care, Welfare and Treatment of Laboratory Animals and were reviewed the Institutional Animal Care and Use Committee either at GSK or by the ethical review process at the institution where the work was performed. RKO, MDA-MB-231, and RPMI-8226 xenograft studies were performed at Charles River (Morrisville, NC). RKO and MDA-MB-231 xenografts were grown in female NCr nu/nu mice (Crl:NU(NCr)-Foxn1^nu^, 10 or 11 weeks old, Charles River). RPMI-8226 xenografts were grown in female CB.17 SCID mice (CB17/Icr-Prkdc^scid^/IcrIcoCrl, 12 weeks old, Charles River). HPAF-II xenografts were grown in female beige SCID mice (CB17.B6-Prkdc<scid>Lyst<bg>Crl, 7.5 weeks old, Charles River). Tumors were measured with calipers and randomized using stratified sampling according to tumor size into groups of 10 mice when tumor volume reached 100–200 mm^3^. No blinding was performed in any study. GSK525762 was prepared as a solution in 1% methylcellulose vehicle containing 0.2% SDS. Trametinib was prepared as a solution in 0.5% Hydroxypropyl methylcellulose containing 0.2% Tween-80. GSK525762, trametinib, and vehicles were administered orally once daily by individual body weight at 10 ml/kg. For combination treatments, doses of GSK525762 and trametinib were staggered by 8 h. Mice were weighed and tumors were measured with calipers twice weekly, and mice were observed daily for any adverse treatment effects. In the HPAF-II study, mice were supplemented with Diet Gel (ClearH_2_O). In all studies, mice were sacrificed using CO_2_ inhalation or cervical dislocation according to AVMA guidelines after tumors exceeded a pre-determined volume, or if body weight loss exceeded pre-determined limits.

For the RKO, MDA-MB-231, and RPMI-8226 studies, each animal was sacrificed when its tumor reached the pre-defined endpoint tumor volume (2000 mm^3^ for RKO and RPMI-8226; 1500 mm^3^ for MDA-MB-231) or final study day, whichever came first, and the time to endpoint (TTE) was calculated. Treatment outcome was determined from percent tumor growth delay (%TGD), defined as the percent increase in median TTE for treated vs. control mice, with differences between two groups deemed statistically significant at *p* *≤* 0.05 using the Mantel–Cox test in GraphPad Prism. For the HPAF-II study, all groups were euthanized on day 21 when vehicle-treated animals began to exceed the pre-defined end-point tumor volume. Tumor growth inhibition (TGI) on day 18 was calculated for the individual treatment groups compared to vehicle, as detailed in Supplemental Methods. Statistical analyses on TGI were performed using the Mann–Whitney test.

### Affymetrix microarray profiling

Two biological replicates per sample were submitted to Expression Analysis (Durham, NC) for RNA isolation and profiling on Affymetrix GeneChip® Human Genome U133 Plus 2.0 arrays. CEL files were RMA normalized using the Affy package^23^ in Bioconductor^24^ and log_2_ transformed. For each gene, the corresponding probeset with the maximal mean expression across the sample set was selected for quantification^25^. For differential expression analysis, the normalized data were fit to the appropriate linear model and pairwise contrasts were performed by applying an empirical Bayes moderated *t*-statistic using the limma Bioconductor package^26^. The resulting *p*-values were adjusted for multiple comparisons by applying the false discovery rate (FDR) correction^27^. Hierarchical clustering was performed in R (www.R-project.org) using a Euclidean distance metric and complete linkage. Gene set enrichment analysis was performed using the javaGSEA application (version 2-2.2, http://software.broadinstitute.org/gsea) using the C6 (oncogenic signatures) collection or full set of gene signatures in the Molecular Signatures Database^28^ where gene lists were pre-ranked using -log(FDR)·sign(log_2_(fold-change)) from the limma analysis. Venn diagrams were generated using BioVenn (http://www.cmbi.ru.nl/cdd/biovenn /index.php).

Microarray datasets have been deposited in GEO under the accession number #GSE112282.

### Western blot, qRT-PCR, and cell cycle analyses

Western blot, qRT-PCR, and cell cycle analyses were performed, as previously described^20^. All Western blot data presented is from a single experiment representative of typical results (biological *n* ≥ 2). Numbers of biological replicates for individual qRT-PCR and cell cycle experiments are indicated in the Figure Legends. Statistical analyses of qRT-PCR results were performed using a two-tailed paired Student’s *t*-test.

## Electronic supplementary material

Supplemental methods

Supplemental figures

Supplemental Table S1

Supplemental Table S2

Supplemental Table S3

Supplemental Table S4

Supplemental Table S5

Supplemental Table S6

Supplemental Table S7

Supplemental Table S8

Supplemental Figure Legends
