# Supplementary material for: MEK inhibitors overcome resistance to BET inhibition across a number of solid and hematologic cancers
Source: Oncogenesis. 2018 Apr 20;7(4):35. doi: 10.1038/s41389-018-0043-9 (PMC5908790; doi:10.1038/s41389-018-0043-9)
Supplement: Supplementary file 1 — Supplemental methods [file 41389_2018_43_MOESM1_ESM.docx]

**Supplemental Materials and Methods:**

**Reagents**

Antibodies used in Western blot experiments are as follows:

| **Antibody Target** | **Vendor** | **Catalog Number** |
| --- | --- | --- |
| Cleaved PARP | Cell Signaling Technology | 9451, 5625 |
| phospho-ERK1/2 | Cell Signaling Technology | 9101 |
| ERK1/2 | Cell Signaling Technology | 4695 |
| FGFR4 | Cell Signaling Technology | 8562 |
| EGR1 | Cell Signaling Technology | 4154 |
| Tubulin | Sigma | T9026 |
| Actin | Sigma | A2228 |

Antibodies used in capillary-based Western experiments are as follows:

| **Antibody Target** | **Vendor** | **Catalog Number** |
| --- | --- | --- |
| phospho-ERK1/2 | Cell Signaling Technology | 4370 |
| ERK1/2 | ProteinSimple | 040-474 |

Antibodies used for IHC experiments are as follows:

| **Antibody Target** | **Vendor** | **Catalog Number** |
| --- | --- | --- |
| phospho-ERK1/2 | Cell Signaling Technology | 4376 |
| ERK1 | Novus | Y72 |
| ERK2 | Cell Signaling | 9108 |

All TaqMan assays for qPCR analysis were obtained from Life Technologies. Specific assay ID numbers or catalog numbers are listed below.

| **Gene Target** | **Catalog number** |
| --- | --- |
| SPRED1 | Hs01084559_m1 |
| SPRED2 | Hs00986220_m1 |
| SPRY2 | Hs01921749_s1 |
| SPRY4 | Hs01935412_s1 |
| EGR1 | Hs00152928_m1 |
| ETV5 | Hs00927557_m1 |
| DUSP4 | Hs01027785_m1 |
| DUSP6 | Hs04329643_s1 |
| ANLN | Hs01122612_m1 |
| ASPM | Hs10411505_m1 |
| BUB1B | Hs01084828_m1 |
| CASC5 | Hs00538241_m1 |
| CCNA2 | Hs00996788_m1 |
| CCNB2 | Hs01084593_g1 |
| CDCA7 | Hs00230589_m1 |
| DEPDC1 | Hs00874226_s1 |
| NDC80 | Hs01092161_m1 |
| NUF2 | Hs00230097_m1 |
| NCAPG | Hs00254617_m1 |
| PBK | Hs00902990_m1 |
| TTK | Hs01009870_m1 |
| GAPDH | 4352934E |

***In vivo* Studies**

For the HPAF-II efficacy study, the percentage of tumor growth inhibition (TGI) was calculated on the final day of dosing with a complete vehicle group (day 18), using the following formula:

1-[(average growth of the drug treated population Day_last_ - average growth of the drug treated population Day_0_) / (average growth of the vehicle treated control population on Day_last_ - average growth of the vehicle treated control population on Day_0_)]*100

All efficacy (% TGI) and statistical (p values) calculations were performed using Study Director Software (Version 3.1.297.184), StudyLog Systems Inc., South San Francisco, CA).

Change in TGI of single agent over combination was performed by subtracting the single agent TGI value from the combination TGI value for each agent. P values between groups were calculated using Study Director Software.

For pharmacodynamic studies, mice were euthanized as described in the Methods section. Tumors were harvested from euthanized mice and flash frozen in liquid nitrogen for RNA isolation as described in the Methods section. Alternatively, tumor portions were formalin-fixed and paraffin-embedded for immunohistochemistry studies as detailed in the Methods.

**Capillary-based Western Analysis**

For capillary-based Western analysis, equivalent amounts of protein were analyzed on a PEGGY instrument (ProteinSimple) using the manufacturer’s size preparation protocol. Briefly, protein lysates were prepared in RIPA buffer as described above and diluted to a concentration previously determined to be suitable for the antibody of interest. Lysates and Western reagents were then loaded into a 384-well plate, following the recommendations of the manufacturer (ProteinSimple). Lysates were then analyzed on the PEGGY instrument (ProteinSimple) using the manufacturer’s size preparation protocol. Peaks were identified, analyzed, and quantified using Compass software (ProteinSimple).

***Ex vivo C*ombination Assays in SCLC Patient-derived Xenografts**

Human biological samples were sourced ethically and their research use was in accord with the terms of the informed consents. *Ex* vivo combination assays in SCLC patient-derived xenografts were performed at Oncotest. Briefly, tumor xenografts growing subcutaneously in serial passages in thymus-aplastic nude mice (NMRI nu/nu strain) were removed under sterile conditions, mechanically disaggregated, and

incubated with an enzyme cocktail consisting of collagenase type IV (41 U/mL), DNase I

(125 U/mL), hyaluronidase (100 U/mL) and dispase II (1.0 U/mL) in RPMI 1640 medium at 37°C for 45

minutes. Cells were passed through sieves of 100 μm and 40 μm mesh size (Cell Strainer, BD Falcon™)

and washed twice with RPMI 1640 medium. Freshly prepared tumor cell suspensions were then

directly subjected to the assay.

The clonogenic assay was performed in a 96 well plate format according to a modified two-layer soft

agar assay ^2^. The test compounds were added after serial dilution in DMSO and transfer to cell culture medium, and left on the cells for the duration of the experiment. Cultures were incubated at 37°C and 7.5% CO2 in a humidified atmosphere for 8-13 days and monitored closely for colony growth using an inverted microscope. At the time of maximum colony formation, counts were performed with an automatic image analysis system (BIOREADER 5000-Vα, Biosys GmbH). 48 hours prior to evaluation, vital colonies were stained with a sterile aqueous solution of 2-(4-iodophenyl)-3-(4-nitrophenyl)-5-phenyltetrazolium chloride.

Drug effects were expressed in terms of the percentage of colony formation, obtained by comparison of

the mean signal in the treated wells with the mean signal of the untreated controls. Two-drug combinations were evaluated using Bliss Independence analysis ^1^. Results are presented in a heatmap format, where positive values (Bliss Index >> 0, blue) indicate synergism, negative values (Bliss Index << 0, red) indicate antagonism, and zero is the neutral value (white). All experiments were performed in biological duplicate.

**Immunohistochemistry**

Immunohistochemistry on paraffin-embedded xenograft tumors was performed by Mosaic Laboratories (Lake Forest, CA), following their standard operating procedures. Slides were subjected to H&E staining, and necrotic tissue was omitted from all analyses. One slide per animal was evaluated for each antibody, and staining across the entire tissue section from each slide was reviewed by a pathologist. Evaluation of reactivity by the pathologist involved a combination of the following: cellular localization of staining, staining intensity, and percentage of cells staining in the tissue type of interest. The p-ERK1/2, ERK1, and ERK2 assays were evaluated on a semiquantitative scale, and the percentage of cells staining at each of the following four levels was recorded: 0 (unstained), 1+ (weak staining), 2+ (moderate staining) and 3+ (strong staining). H-scores were calculated based on the summation of the product of percent of cells stained at each intensity using the following equation: (3 x % cells staining at 3+) + (2 x % cells staining at 2+) + (1 x % cells staining at 1+). Slides were scanned using an Aperio ScanScope CS system (Aperio, Vista, CA) to produce whole slide images.

**References**

1 Bliss CI. The Toxicity of Poisons Applied Jointly. Annals of Applied Biology 1939; 26: 585-615.

2 Hamburger AW, Salmon SE. Primary bioassay of human tumor stem cells. Science 1977; 197: 461-463.
