# Supplementary material for: MEK inhibitors overcome resistance to BET inhibition across a number of solid and hematologic cancers
Source: Oncogenesis. 2018 Apr 20;7(4):35. doi: 10.1038/s41389-018-0043-9 (PMC5908790; doi:10.1038/s41389-018-0043-9)
Supplement: Supplementary file 2 — Supplemental figures [file 41389_2018_43_MOESM2_ESM.pptx]

## Slide 1
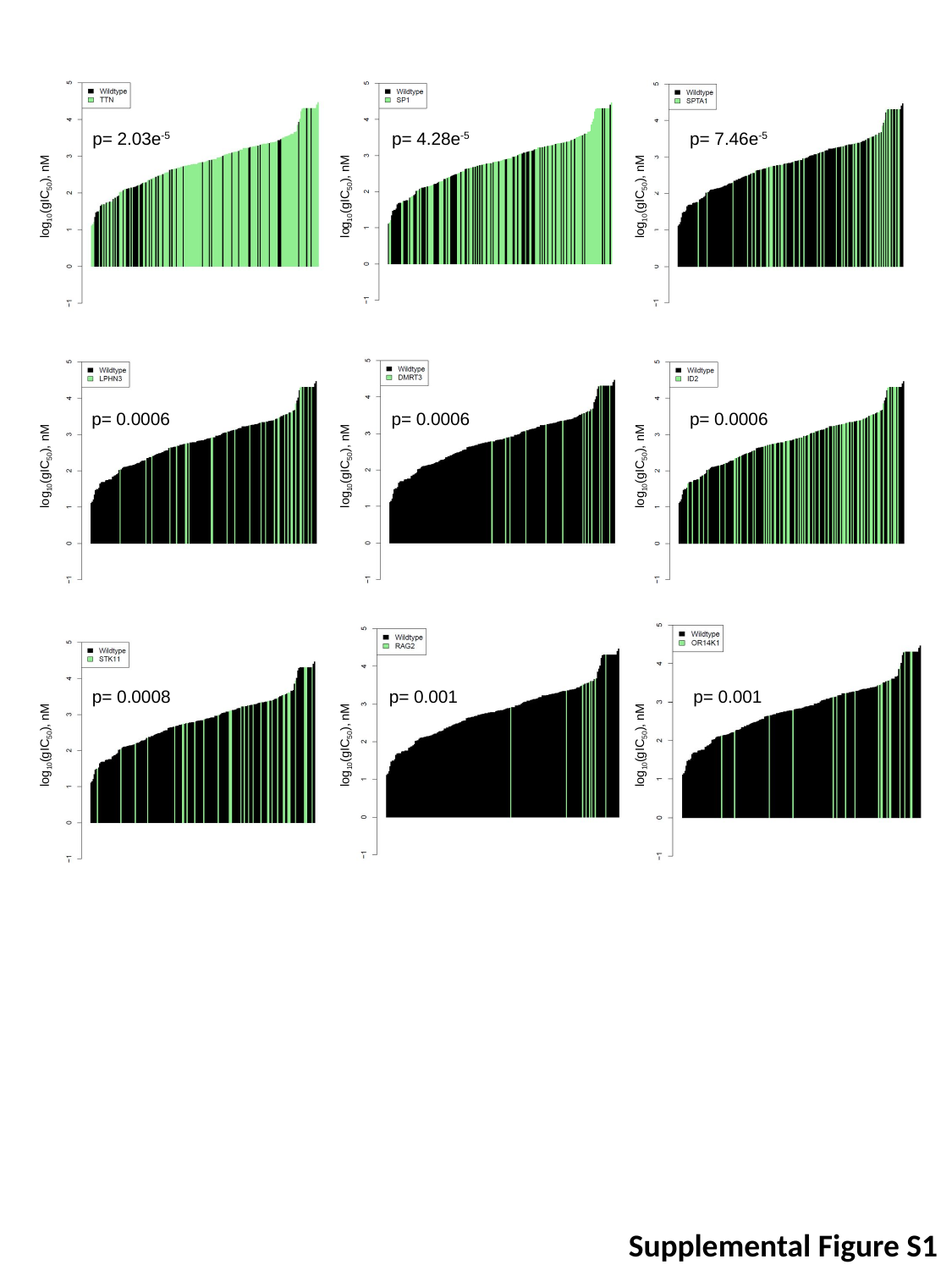

p= 2.03e-5
p= 4.28e-5
p= 7.46e-5
log10(gIC50), nM
log10(gIC50), nM
log10(gIC50), nM
p= 0.0006
p= 0.0006
p= 0.0006
log10(gIC50), nM
log10(gIC50), nM
log10(gIC50), nM
p= 0.0008
p= 0.001
p= 0.001
log10(gIC50), nM
log10(gIC50), nM
log10(gIC50), nM
Supplemental Figure S1

## Slide 2
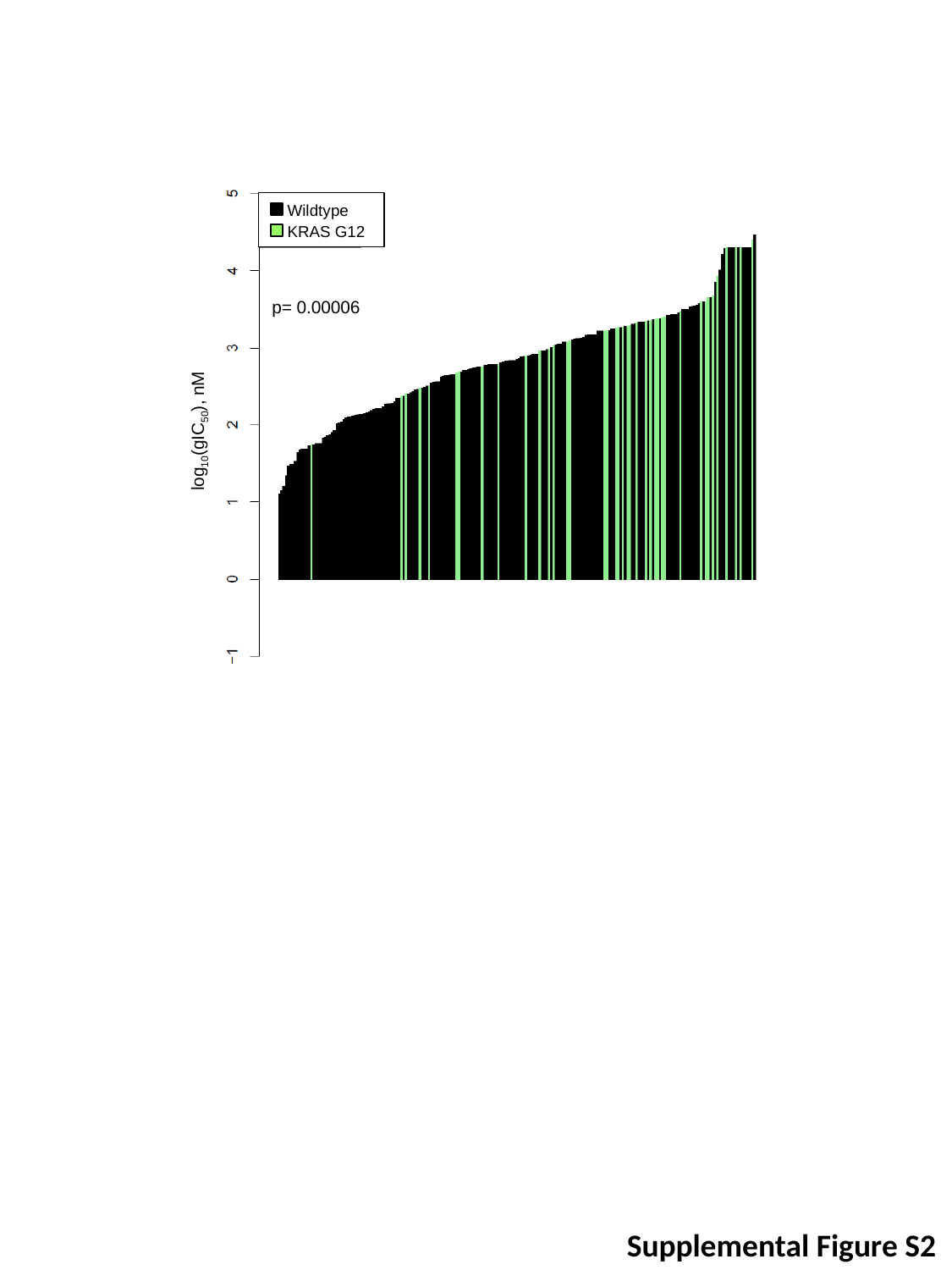

Wildtype
KRAS G12
p= 0.00006
log10(gIC50), nM
Supplemental Figure S2

## Slide 3
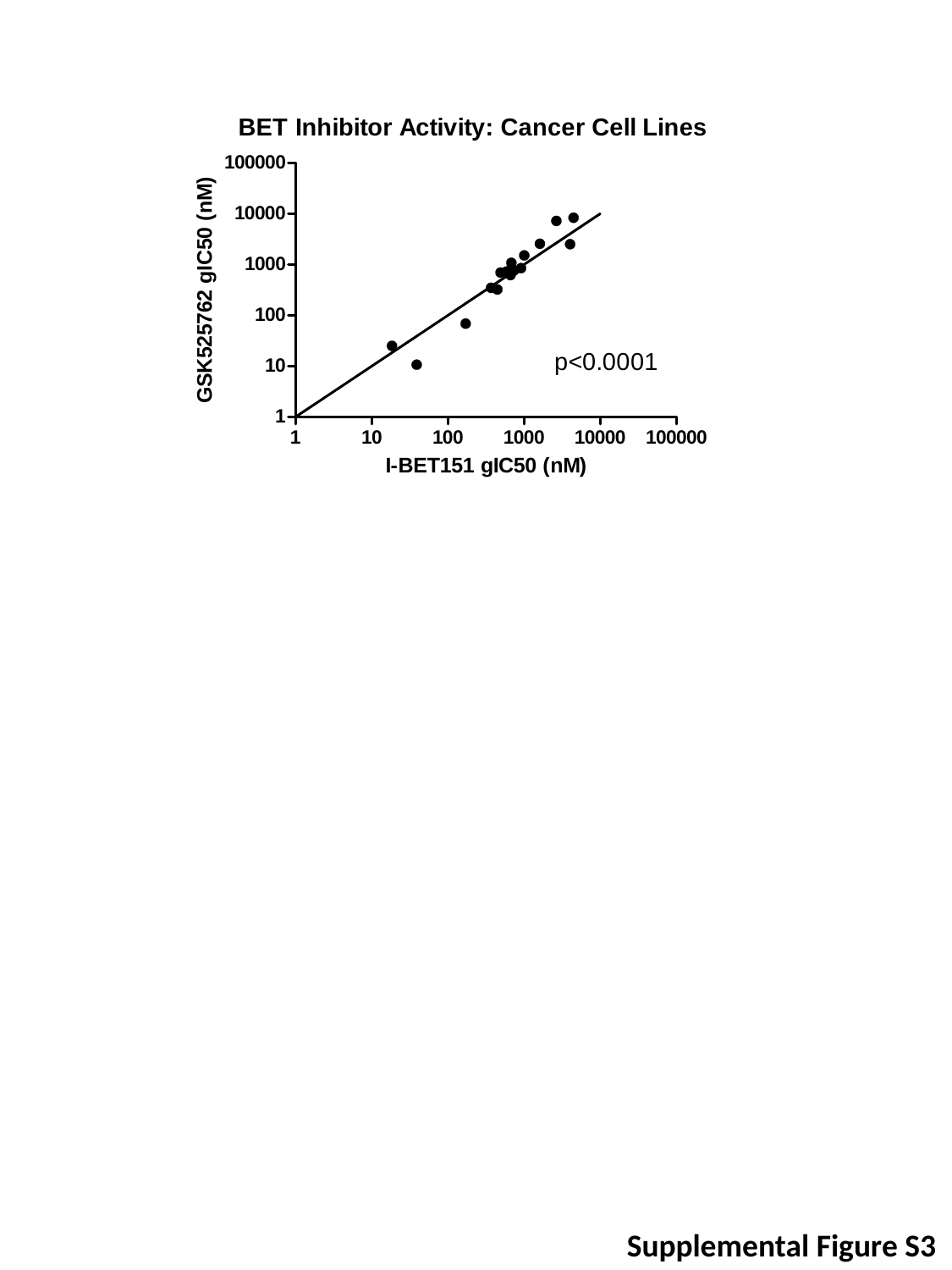

Supplemental Figure S3

## Slide 4
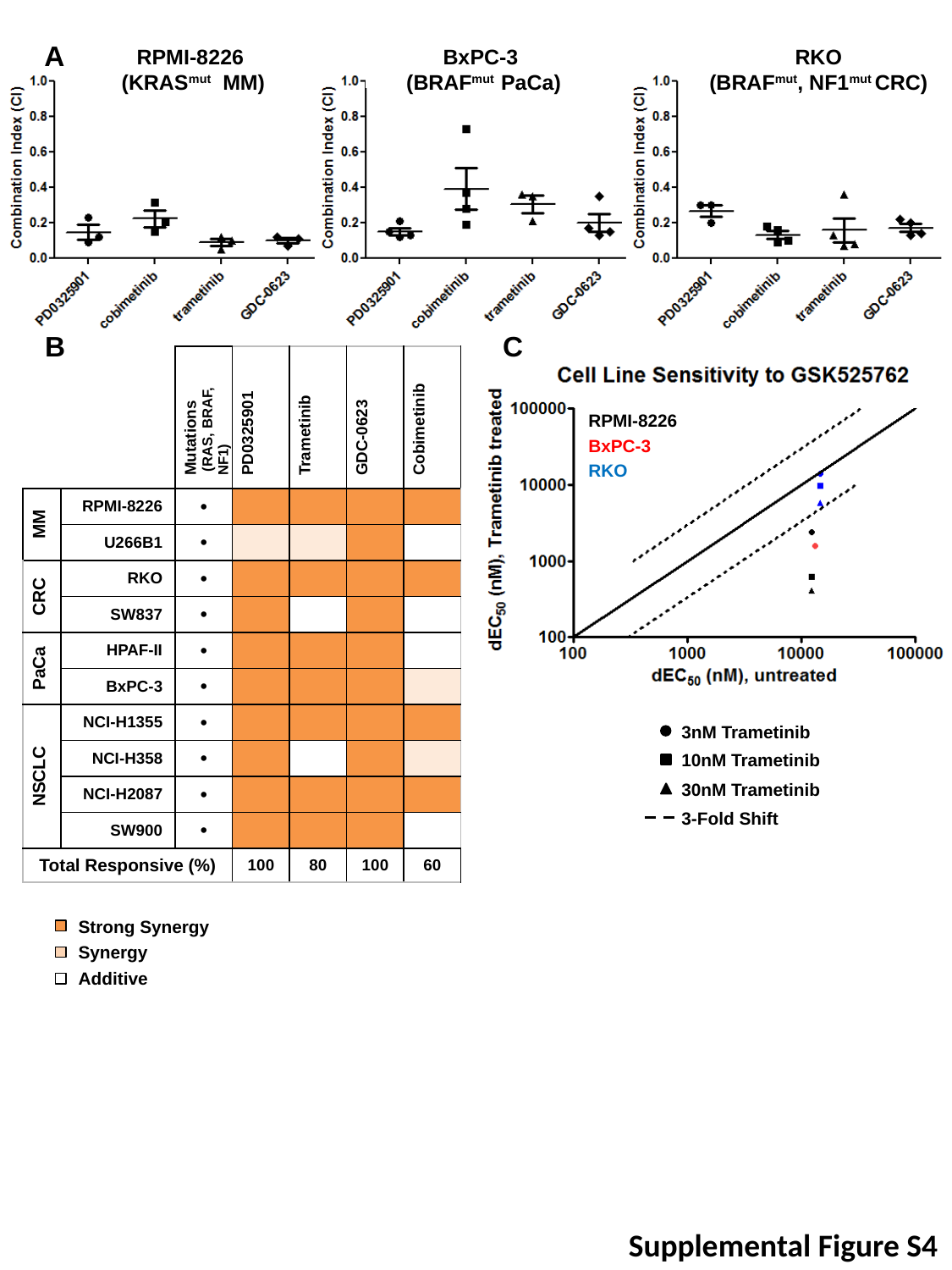

A
RPMI-8226
(KRASmut MM)
BxPC-3
(BRAFmut PaCa)
RKO
(BRAFmut, NF1mut CRC)
B
C
| | | Mutations (RAS, BRAF, NF1) | PD0325901 | Trametinib | GDC-0623 | Cobimetinib |
| --- | --- | --- | --- | --- | --- | --- |
| MM | RPMI-8226 | • | | | | |
| | U266B1 | • | | | | |
| CRC | RKO | • | | | | |
| | SW837 | • | | | | |
| PaCa | HPAF-II | • | | | | |
| | BxPC-3 | • | | | | |
| NSCLC | NCI-H1355 | • | | | | |
| | NCI-H358 | • | | | | |
| | NCI-H2087 | • | | | | |
| | SW900 | • | | | | |
| Total Responsive (%) | | | 100 | 80 | 100 | 60 |
RPMI-8226
BxPC-3
RKO
3nM Trametinib
10nM Trametinib
30nM Trametinib
3-Fold Shift
Strong Synergy
Synergy
Additive
Supplemental Figure S4

## Slide 5
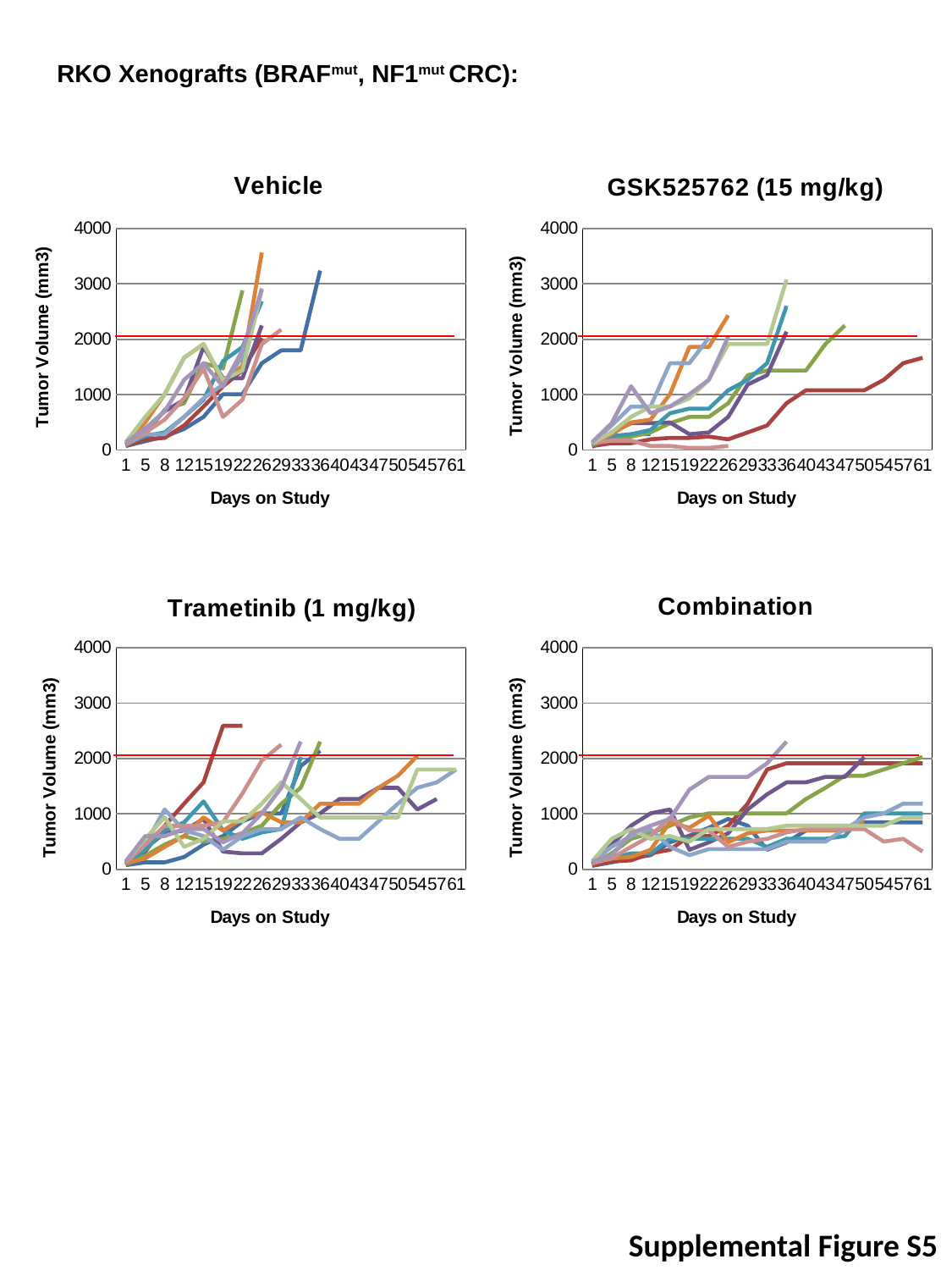

RKO Xenografts (BRAFmut, NF1mut CRC):
[unsupported chart]
[unsupported chart]
[unsupported chart]
[unsupported chart]
Supplemental Figure S5

## Slide 6
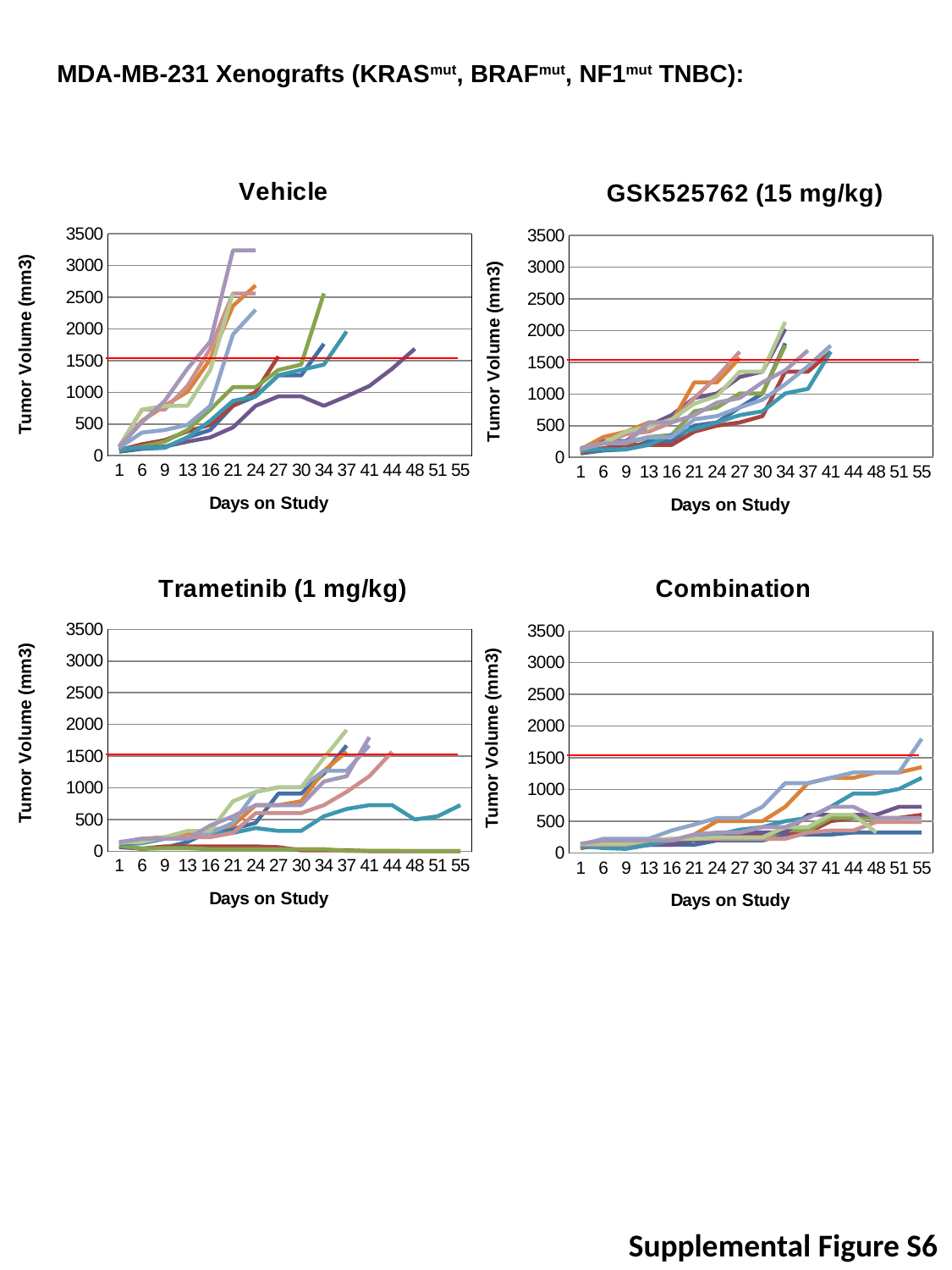

MDA-MB-231 Xenografts (KRASmut, BRAFmut, NF1mut TNBC):
[unsupported chart]
[unsupported chart]
[unsupported chart]
[unsupported chart]
Supplemental Figure S6

## Slide 7
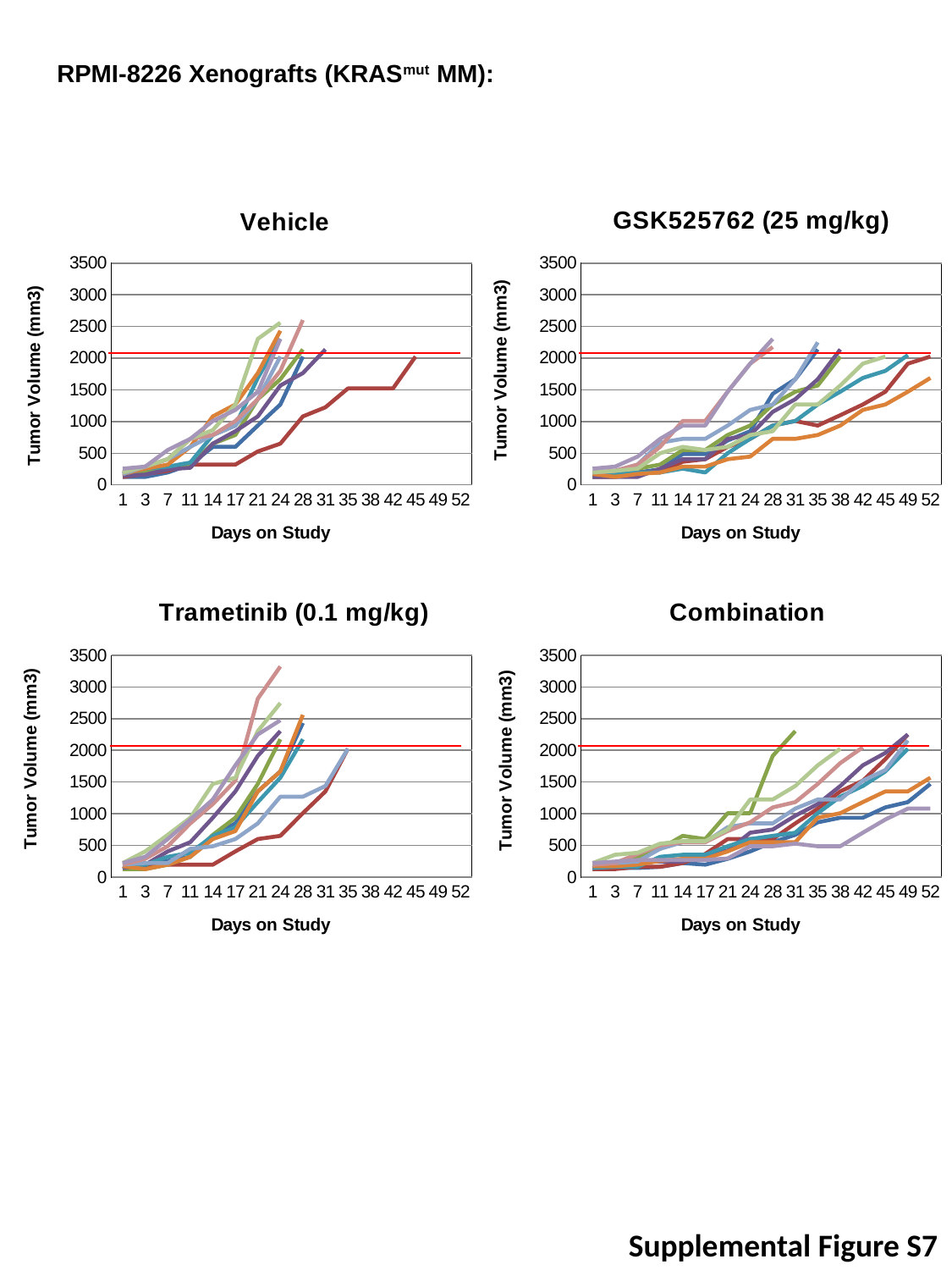

RPMI-8226 Xenografts (KRASmut MM):
[unsupported chart]
[unsupported chart]
[unsupported chart]
[unsupported chart]
Supplemental Figure S7

## Slide 8
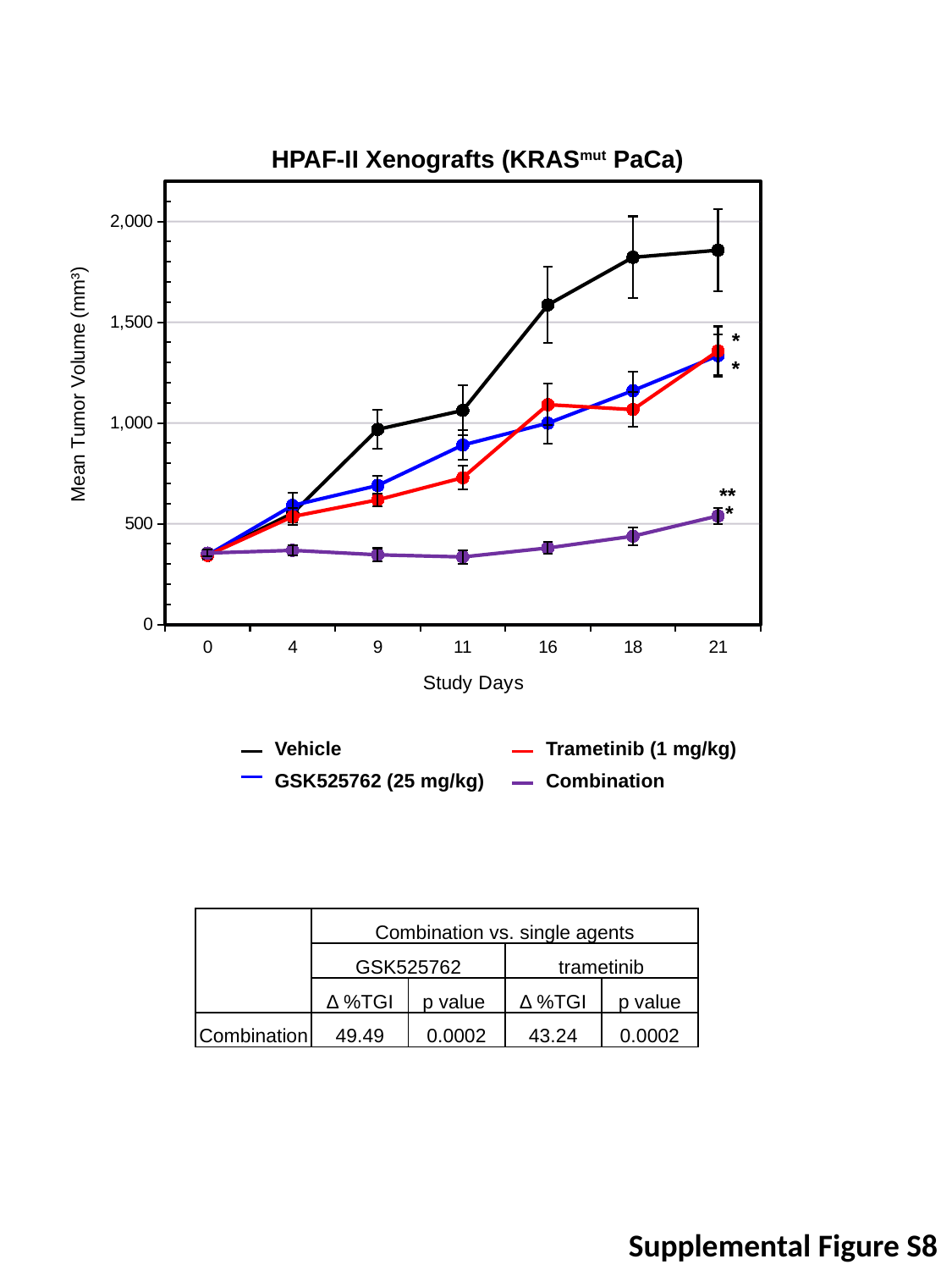

HPAF-II Xenografts (KRASmut PaCa)
### Chart
| Category | Group 1 Vehicle | Group 3 25 mg/kg GSK525762A | Group 5 1 mg/kg trametinib | Group 9 25 mg/kg GSK525762A + 1 mg/kg trametinib |
|---|---|---|---|---|
| 0 | 342.32508399999995 | 344.1380960499987 | 342.9109157499916 | 354.247127049999 |
| 4 | 551.89998115 | 590.6094886000005 | 535.2785860500156 | 367.66893834999894 |
| 9 | 967.9942994500001 | 689.5822909999995 | 618.4342573499995 | 345.4805456 |
| 11 | 1062.3190041 | 890.4796735000035 | 728.3516445499995 | 334.9773270999996 |
| 16 | 1585.8811480999586 | 999.0984900500035 | 1090.99790835 | 379.50349715000004 |
| 18 | 1822.0042686000002 | 1160.2388338 | 1066.436631350001 | 438.0324853 |
| 21 | 1857.4310496666667 | 1334.2443251999998 | 1357.429039 | 538.5730503888935 |*
*
**
*
Vehicle
Trametinib (1 mg/kg)
GSK525762 (25 mg/kg)
Combination
| | Combination vs. single agents | | | |
| --- | --- | --- | --- | --- |
| | GSK525762 | | trametinib | |
| | Δ %TGI | p value | Δ %TGI | p value |
| Combination | 49.49 | 0.0002 | 43.24 | 0.0002 |
Supplemental Figure S8

## Slide 9
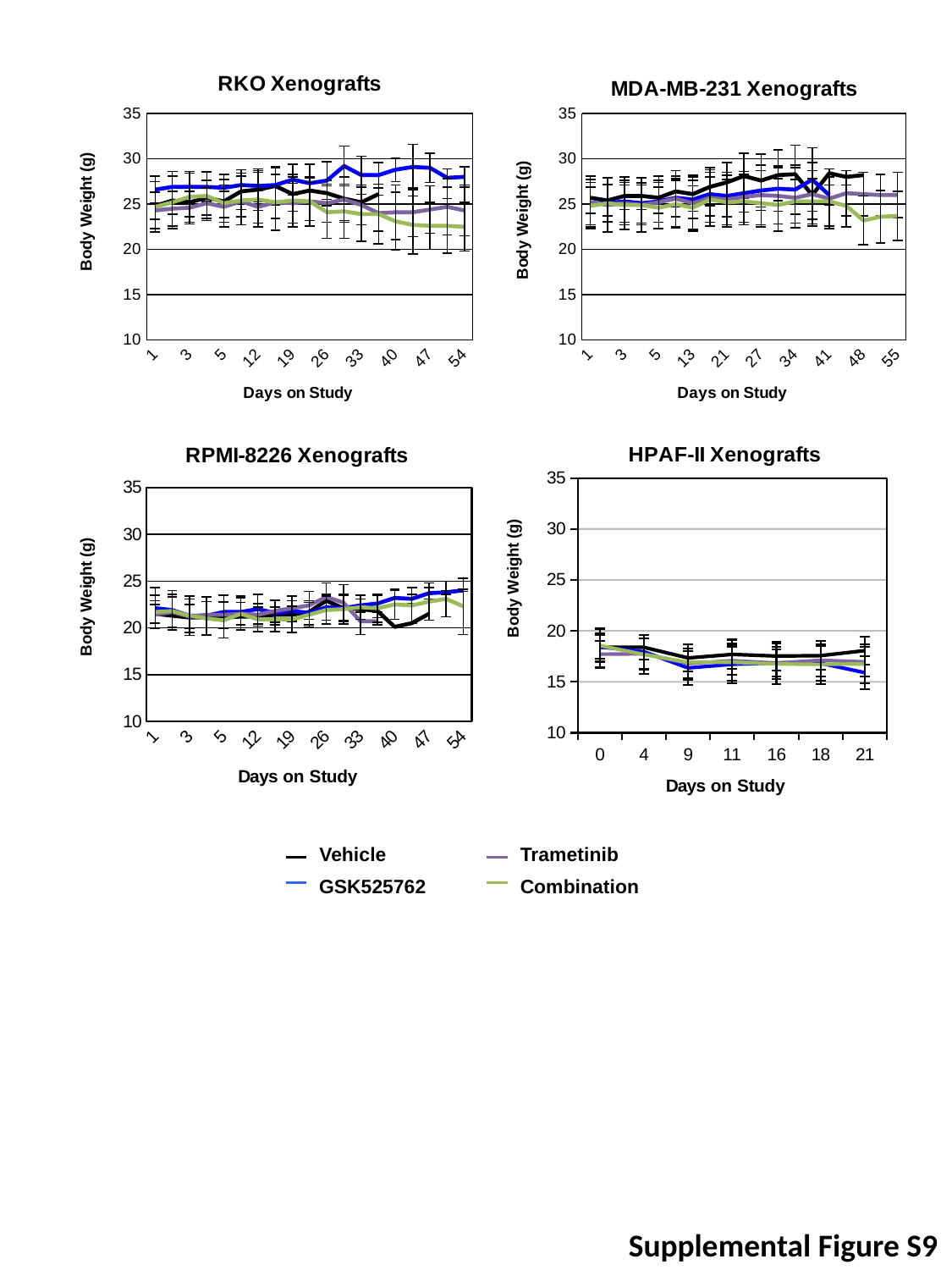

[unsupported chart]
[unsupported chart]
[unsupported chart]
### Chart: HPAF-II Xenografts
| Category | Group 1 Vehicle | Group 3 25 mg/kg GSK525762A | Group 5 1 mg/kg trametinib | Group 9 25 mg/kg GSK525762A + 1 mg/kg trametinib |
|---|---|---|---|---|
| 0 | 18.39 | 18.43 | 17.71 | 18.56 |
| 4 | 18.38 | 17.95 | 17.739999999999988 | 17.68 |
| 9 | 17.34 | 16.36 | 16.8 | 16.91 |
| 11 | 17.69 | 16.72 | 17.07 | 16.92 |
| 16 | 17.53 | 16.85 | 16.84 | 16.75 |
| 18 | 17.57 | 16.82 | 17.11000000000003 | 16.71 |
| 21 | 18.055555555555554 | 15.9 | 16.959999999999987 | 16.755555555555556 |Vehicle
Trametinib
GSK525762
Combination
Supplemental Figure S9

## Slide 10
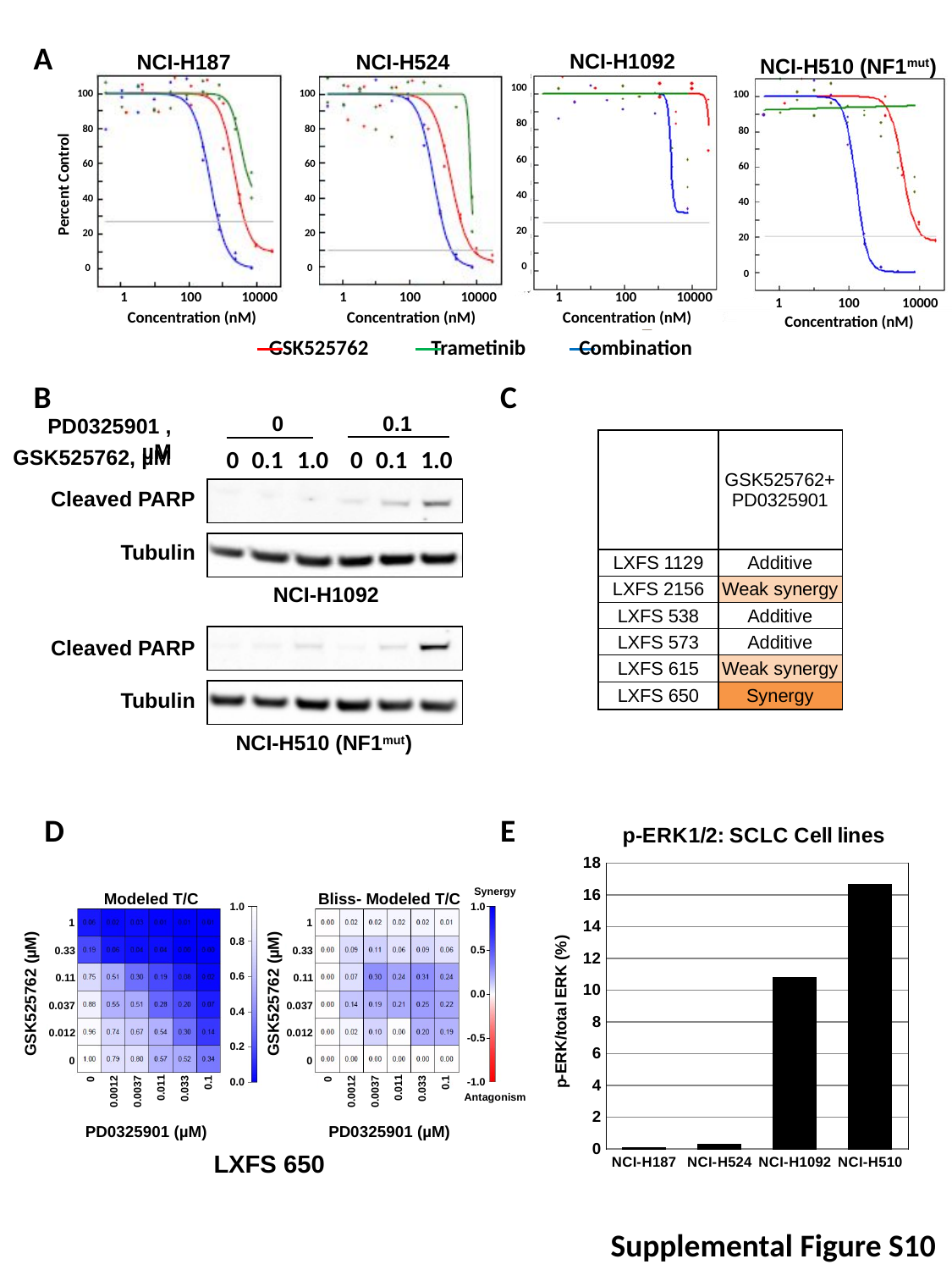

NCI-H1092
100
80
60
40
20
0
1
100
10000
Concentration (nM)
NCI-H187
100
80
60
Percent Control
40
20
0
1
100
10000
Concentration (nM)
NCI-H524
100
80
60
40
20
0
1
100
10000
Concentration (nM)
NCI-H510 (NF1mut)
Concentration (nM)
100
80
60
40
20
0
1
100
10000
A
GSK525762
Trametinib
Combination
B
C
0
0.1
PD0325901 , µM
GSK525762, µM
0
0.1
1.0
0
0.1
1.0
Cleaved PARP
Tubulin
NCI-H1092
Cleaved PARP
Tubulin
NCI-H510 (NF1mut)
| | GSK525762+PD0325901 |
| --- | --- |
| LXFS 1129 | Additive |
| LXFS 2156 | Weak synergy |
| LXFS 538 | Additive |
| LXFS 573 | Additive |
| LXFS 615 | Weak synergy |
| LXFS 650 | Synergy |
### Chart: p-ERK1/2: SCLC Cell lines
| Category | |
|---|---|
| NCI-H187 | 0.08906254489039563 |
| NCI-H524 | 0.2912186379928379 |
| NCI-H1092 | 10.818527918781726 |
| NCI-H510 | 16.652656018574117 |D
E
Synergy
Modeled T/C
Bliss- Modeled T/C
LXFS 650
1.0
1.0
0.5
0.0
-0.5
-1.0
1
1
0.33
0.11
0.037
0.012
0
0.8
0.33
0.6
0.11
GSK525762 (µM)
GSK525762 (µM)
0.037
0.4
0.012
0.2
0
0
0
0.0
Antagonism
0.0012
0.0037
0.011
0.033
0.1
0.0012
0.0037
0.011
0.033
0.1
PD0325901 (µM)
PD0325901 (µM)
Supplemental Figure S10

## Slide 11
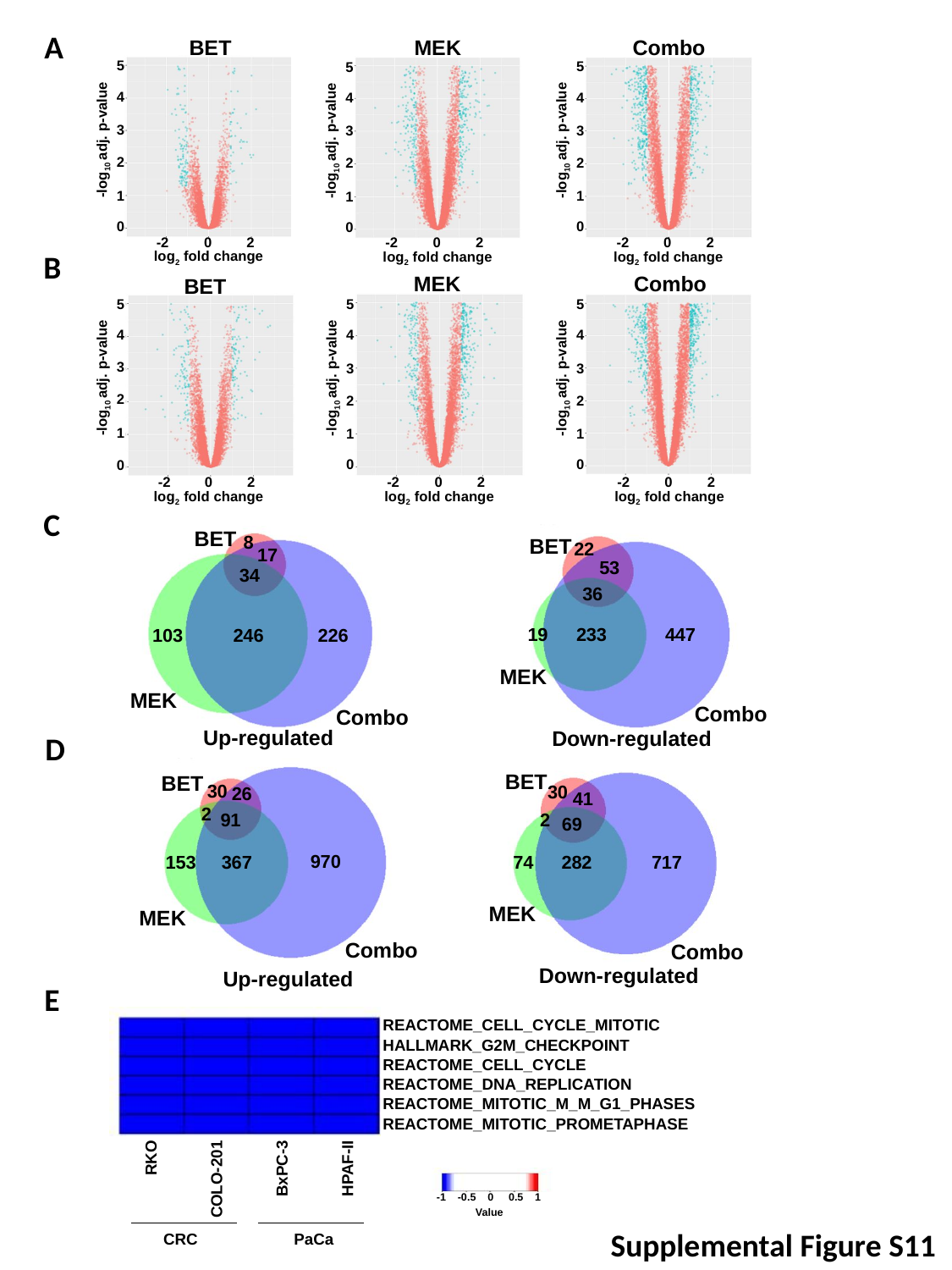

A
MEK
5
4
3
-log10 adj. p-value
2
1
0
-2
0
2
log2 fold change
Combo
5
4
3
-log10 adj. p-value
2
1
0
-2
0
2
log2 fold change
BET
5
4
3
-log10 adj. p-value
2
1
0
-2
0
2
log2 fold change
B
B
MEK
5
4
3
-log10 adj. p-value
2
1
0
-2
0
2
log2 fold change
Combo
5
4
3
-log10 adj. p-value
2
1
0
-2
0
2
log2 fold change
BET
5
4
3
-log10 adj. p-value
2
1
0
-2
0
2
log2 fold change
C
BET
8
17
34
103
246
226
MEK
Combo
Up-regulated
BET
22
53
36
19
233
447
MEK
Combo
Down-regulated
D
BET
30
26
2
91
970
153
367
MEK
Combo
Up-regulated
BET
30
41
2
69
74
282
717
MEK
Combo
Down-regulated
E
REACTOME_CELL_CYCLE_MITOTIC
HALLMARK_G2M_CHECKPOINT
REACTOME_CELL_CYCLE
REACTOME_DNA_REPLICATION
REACTOME_MITOTIC_M_M_G1_PHASES
REACTOME_MITOTIC_PROMETAPHASE
RKO
BxPC-3
HPAF-II
COLO-201
-1
-0.5
0
0.5
1
Value
CRC
PaCa
Supplemental Figure S11

## Slide 12
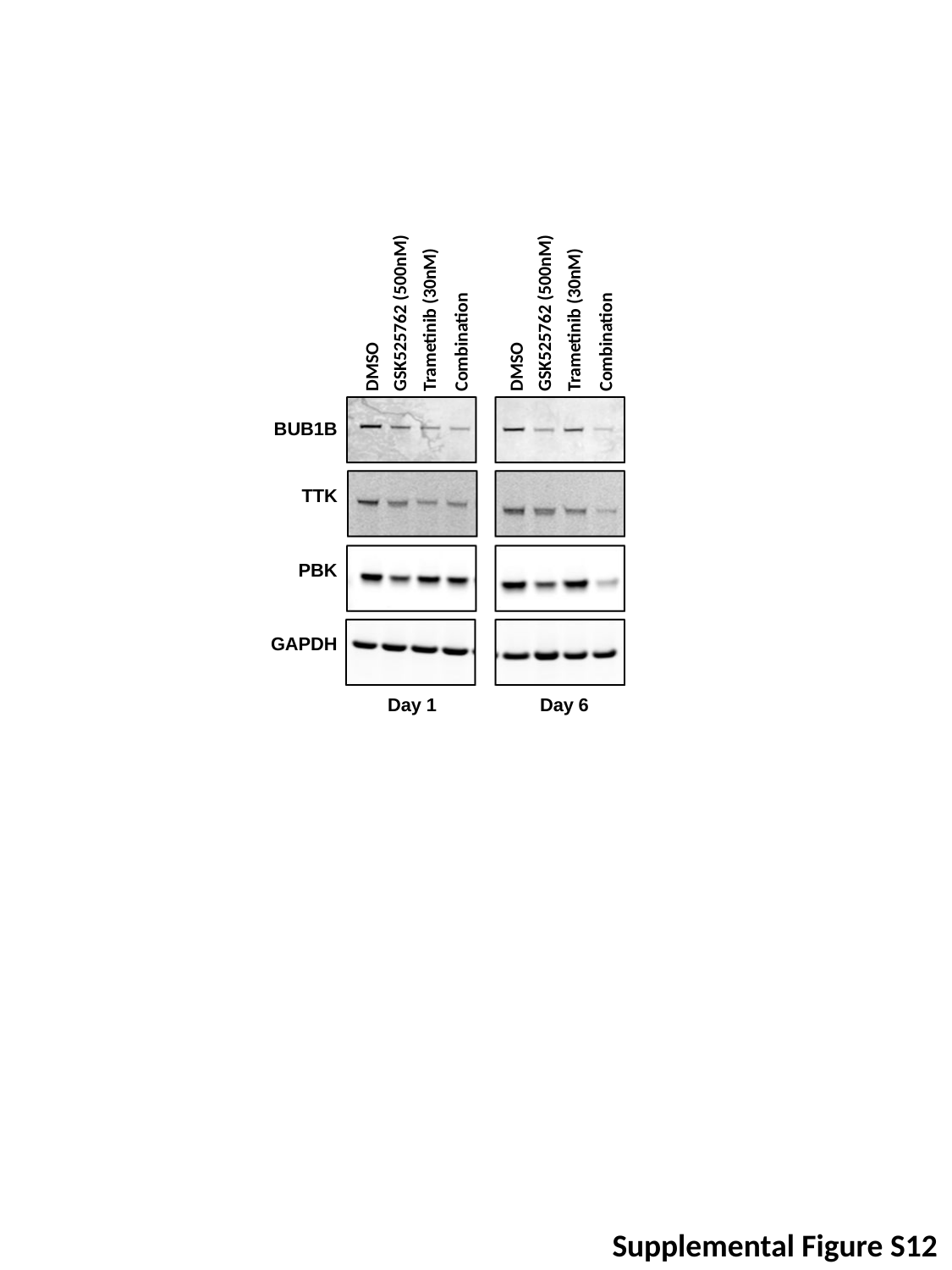

Trametinib (30nM)
GSK525762 (500nM)
Combination
Trametinib (30nM)
GSK525762 (500nM)
Combination
DMSO
DMSO
BUB1B
TTK
PBK
GAPDH
Day 1
Day 6
Supplemental Figure S12

## Slide 13
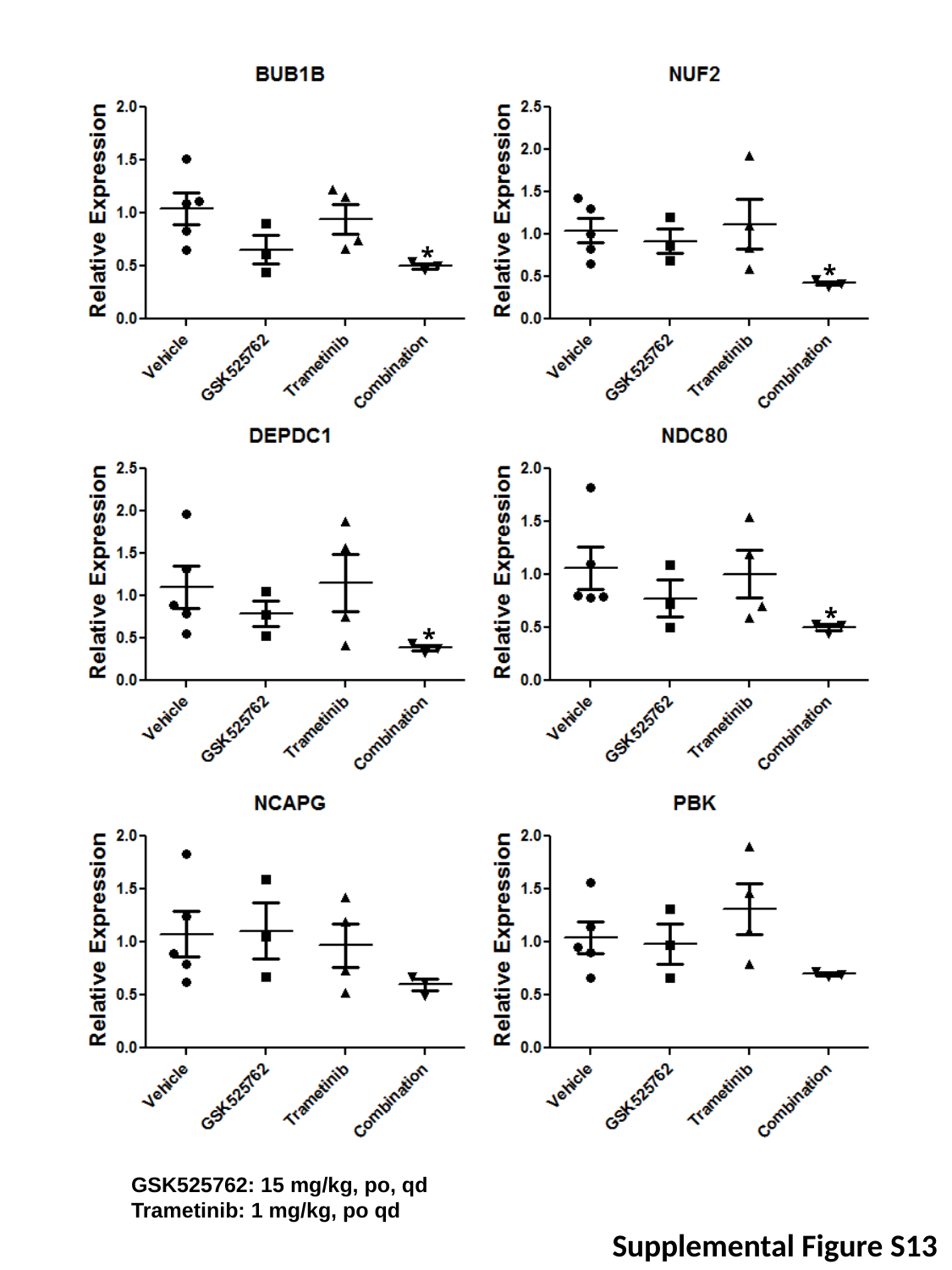

*
*
*
*
GSK525762: 15 mg/kg, po, qd
Trametinib: 1 mg/kg, po qd
Supplemental Figure S13

## Slide 14
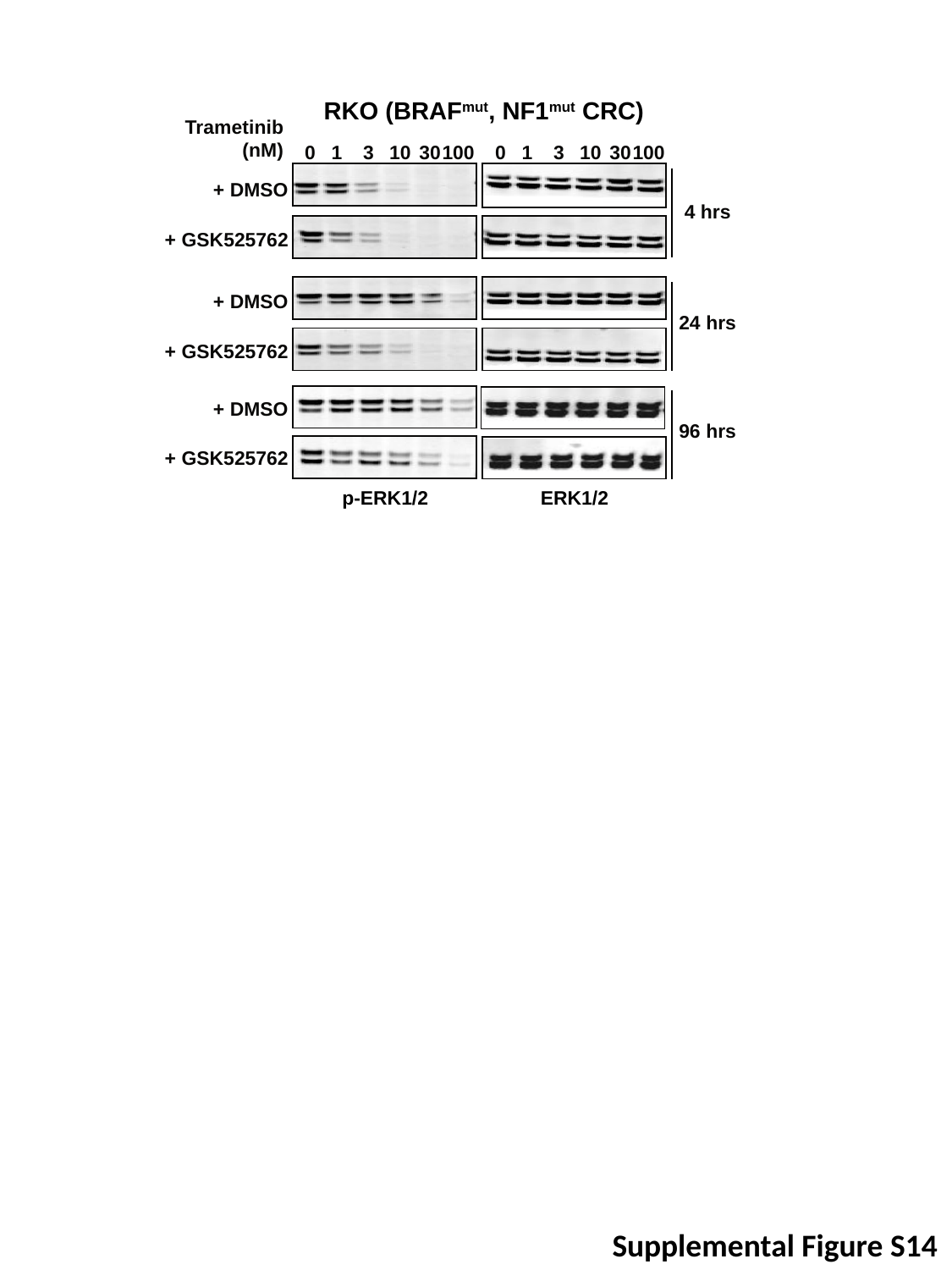

RKO (BRAFmut, NF1mut CRC)
Trametinib
 (nM)
0
1
3
10
30
100
0
1
3
10
30
100
+ DMSO
4 hrs
+ GSK525762
+ DMSO
24 hrs
+ GSK525762
+ DMSO
96 hrs
+ GSK525762
p-ERK1/2
ERK1/2
Supplemental Figure S14

## Slide 15
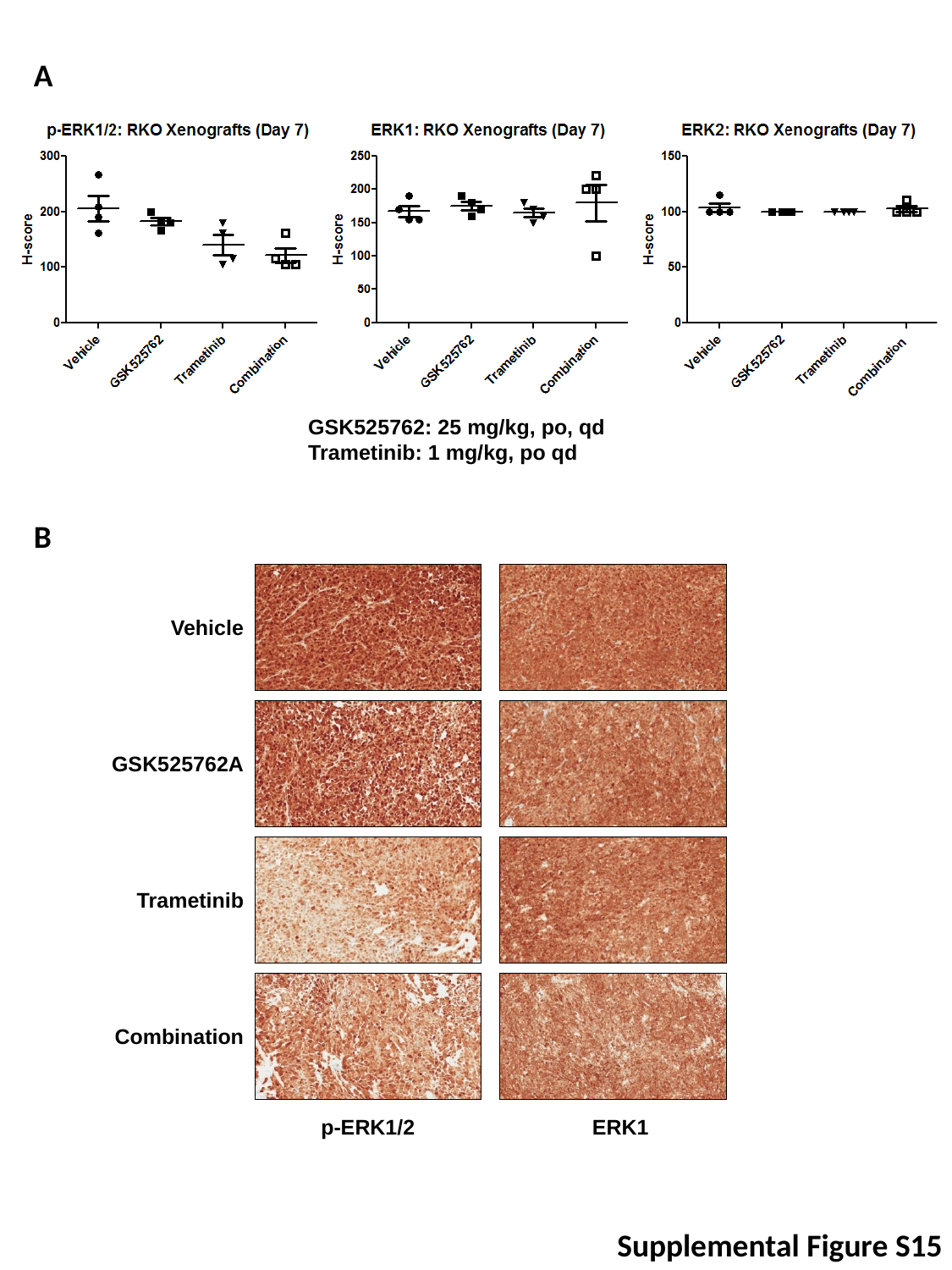

A
GSK525762: 25 mg/kg, po, qd
Trametinib: 1 mg/kg, po qd
B
Vehicle
GSK525762A
Trametinib
Combination
p-ERK1/2
ERK1
Supplemental Figure S15

## Slide 16
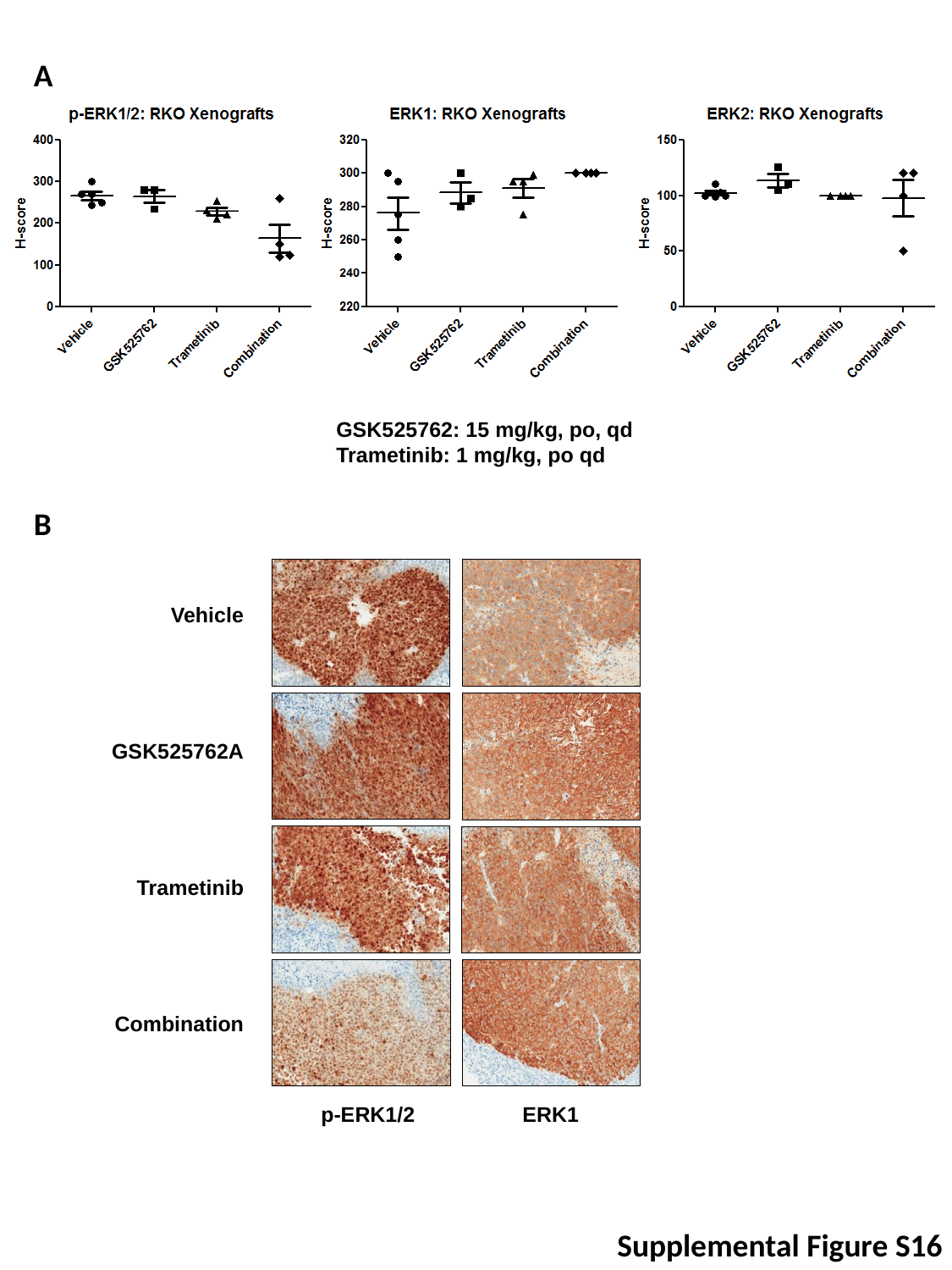

A
GSK525762: 15 mg/kg, po, qd
Trametinib: 1 mg/kg, po qd
B
Vehicle
GSK525762A
Trametinib
Combination
p-ERK1/2
ERK1
Supplemental Figure S16

## Slide 17
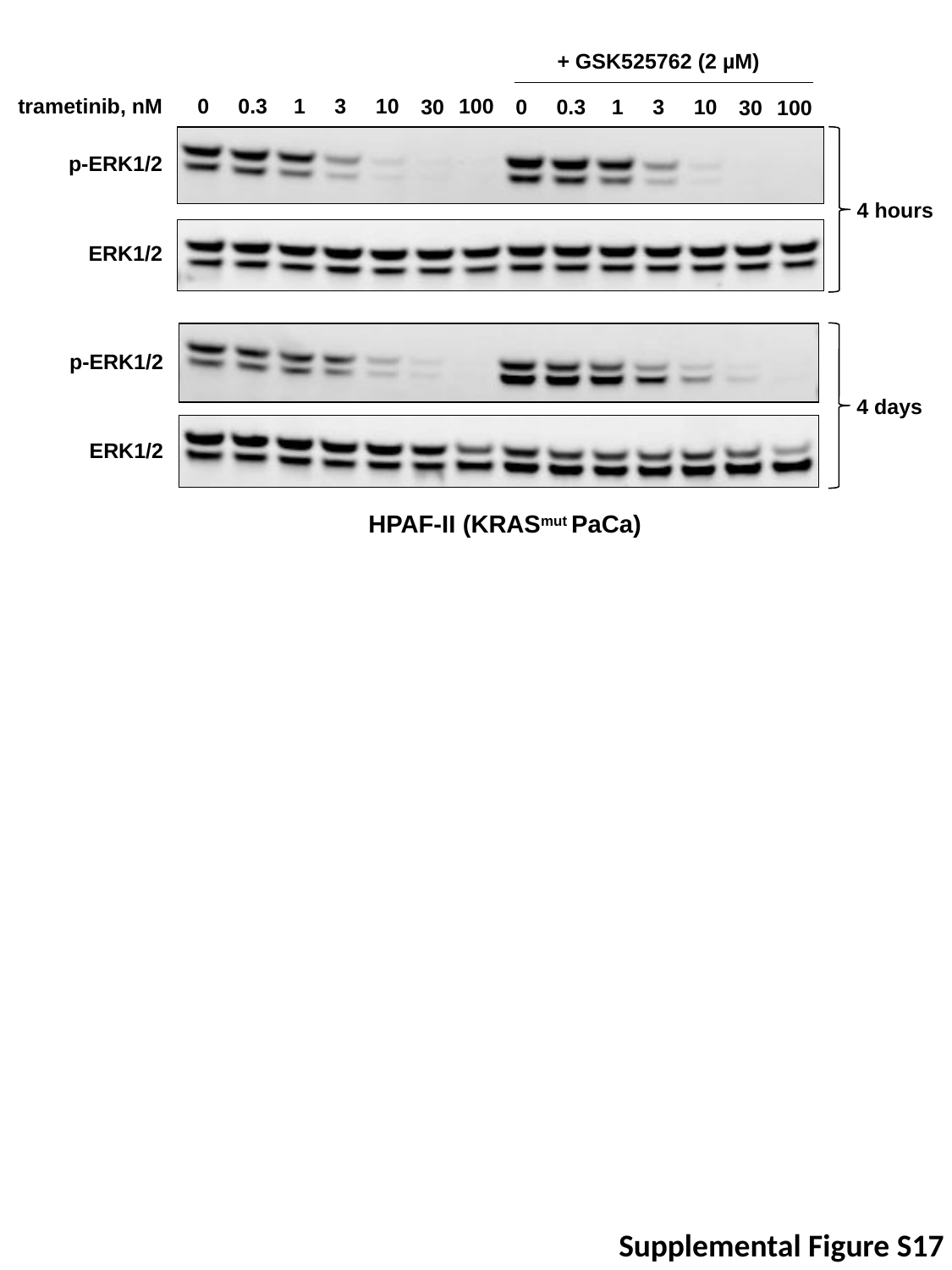

+ GSK525762 (2 µM)
trametinib, nM
0
0.3
1
3
10
100
30
0
0.3
1
3
10
100
30
p-ERK1/2
ERK1/2
4 hours
p-ERK1/2
4 days
ERK1/2
HPAF-II (KRASmut PaCa)
Supplemental Figure S17

## Slide 18
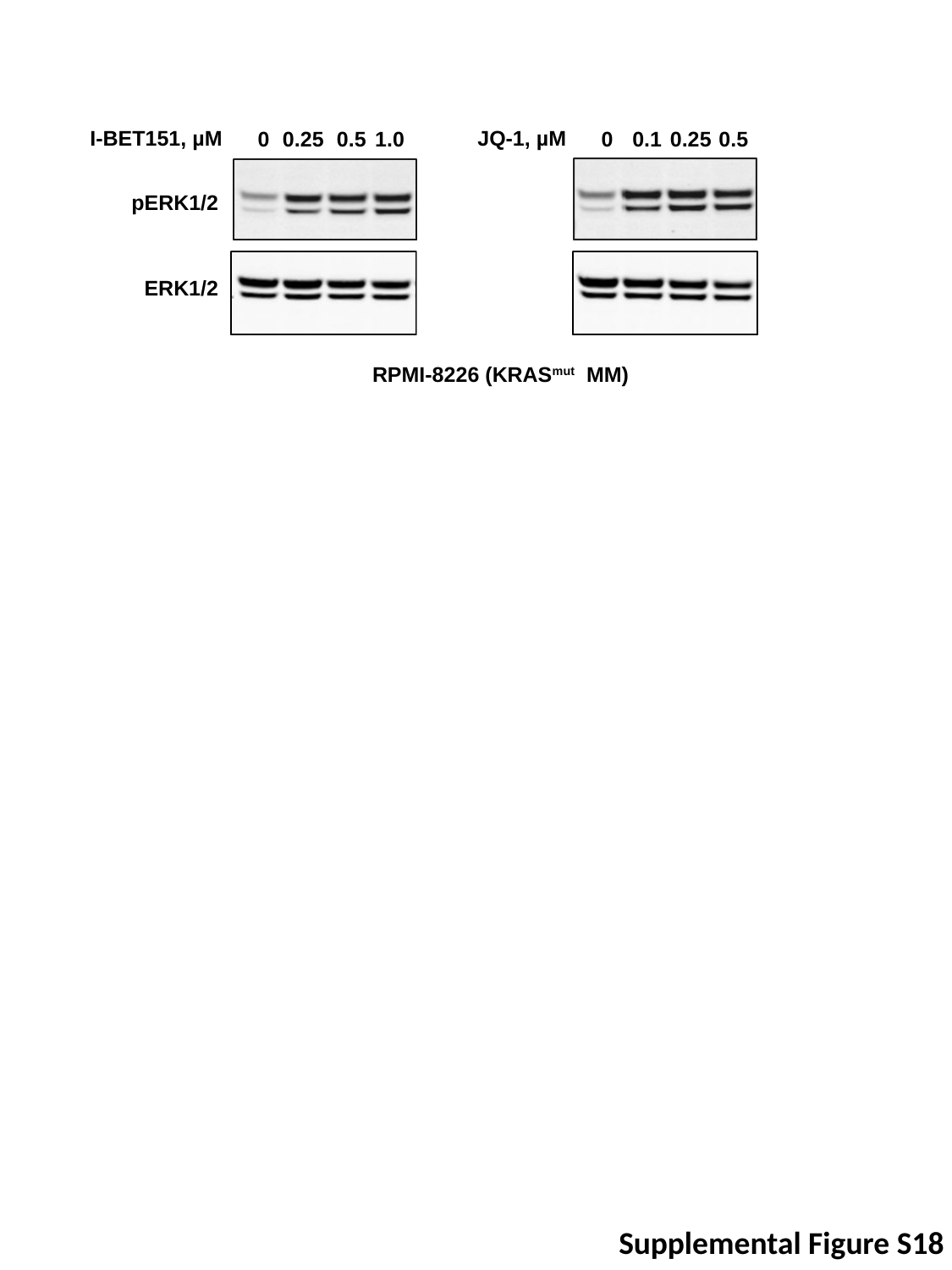

I-BET151, µM
JQ-1, µM
0
0.25
0.5
1.0
0
0.1
0.25
0.5
pERK1/2
ERK1/2
RPMI-8226 (KRASmut MM)
Supplemental Figure S18

## Slide 19
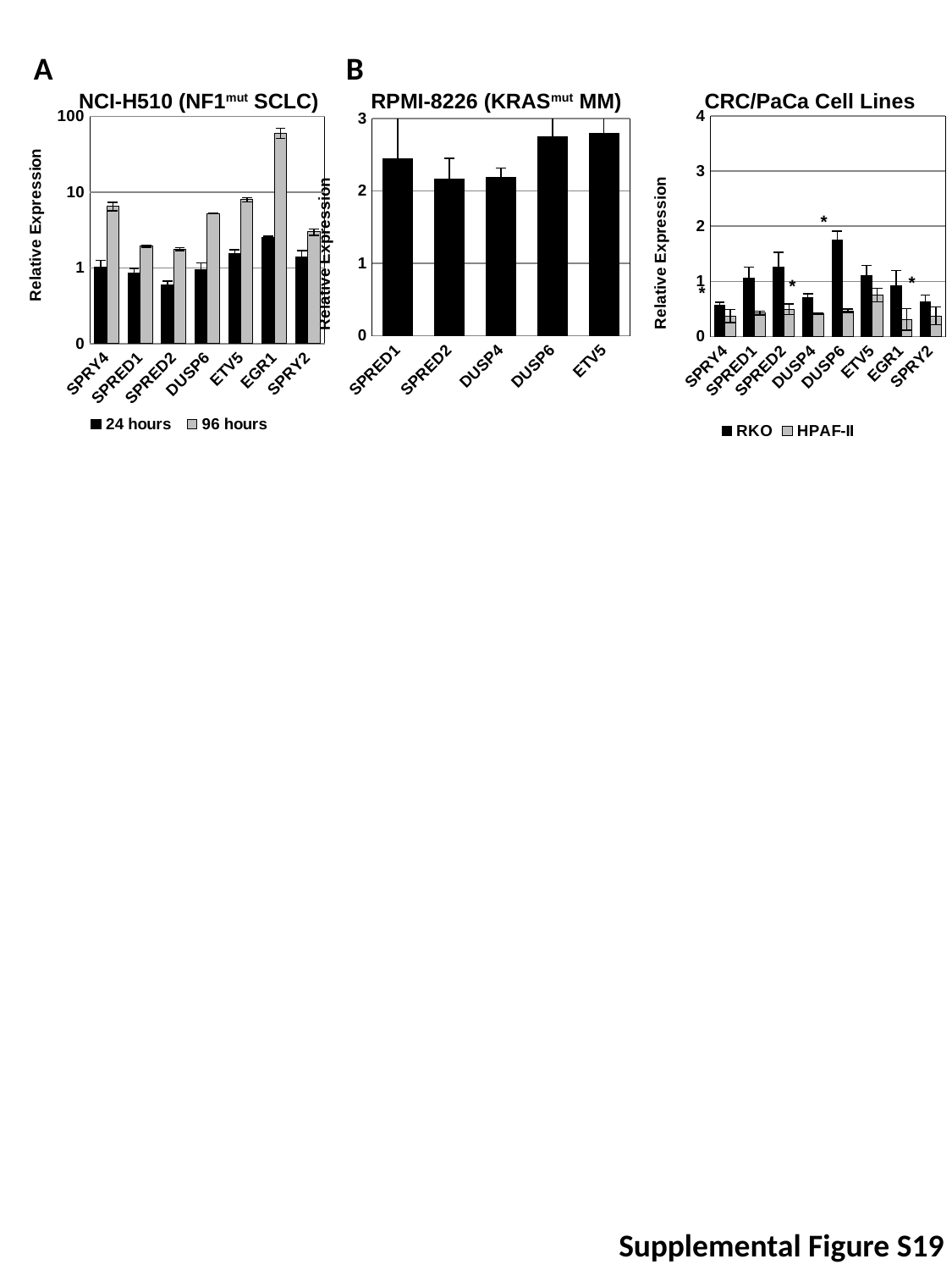

A
B
NCI-H510 (NF1mut SCLC)
### Chart
| Category | | |
|---|---|---|
| SPRY4 | 1.0319069476969718 | 6.4948451103823714 |
| SPRED1 | 0.859686054785189 | 1.9382549642096067 |
| SPRED2 | 0.5918610731764308 | 1.7765712252022559 |
| DUSP6 | 0.9463466658955115 | 5.211449038192916 |
| ETV5 | 1.5599053691031548 | 7.971169970503201 |
| EGR1 | 2.548200365795321 | 60.59558721094688 |
| SPRY2 | 1.384886334069483 | 2.993055153295654 |RPMI-8226 (KRASmut MM)
### Chart
| Category | |
|---|---|
| SPRED1 | 2.4415793235865904 |
| SPRED2 | 2.1661704800538577 |
| DUSP4 | 2.186569377370144 |
| DUSP6 | 2.748715520684087 |
| ETV5 | 2.7996328792944447 |CRC/PaCa Cell Lines
### Chart
| Category | RKO | HPAF-II |
|---|---|---|
| SPRY4 | 0.568612565703008 | 0.3689297821548081 |
| SPRED1 | 1.0590957410888509 | 0.4245358031862928 |
| SPRED2 | 1.2624360015293399 | 0.49159098168602033 |
| DUSP4 | 0.7104655824400896 | 0.4108274085887436 |
| DUSP6 | 1.7527875646507465 | 0.4640689382382769 |
| ETV5 | 1.0981107025913661 | 0.7515253509662472 |
| EGR1 | 0.921455210978639 | 0.3071839158814976 |
| SPRY2 | 0.6299116373038351 | 0.3720583296311845 |*
*
*
*
Supplemental Figure S19

## Slide 20
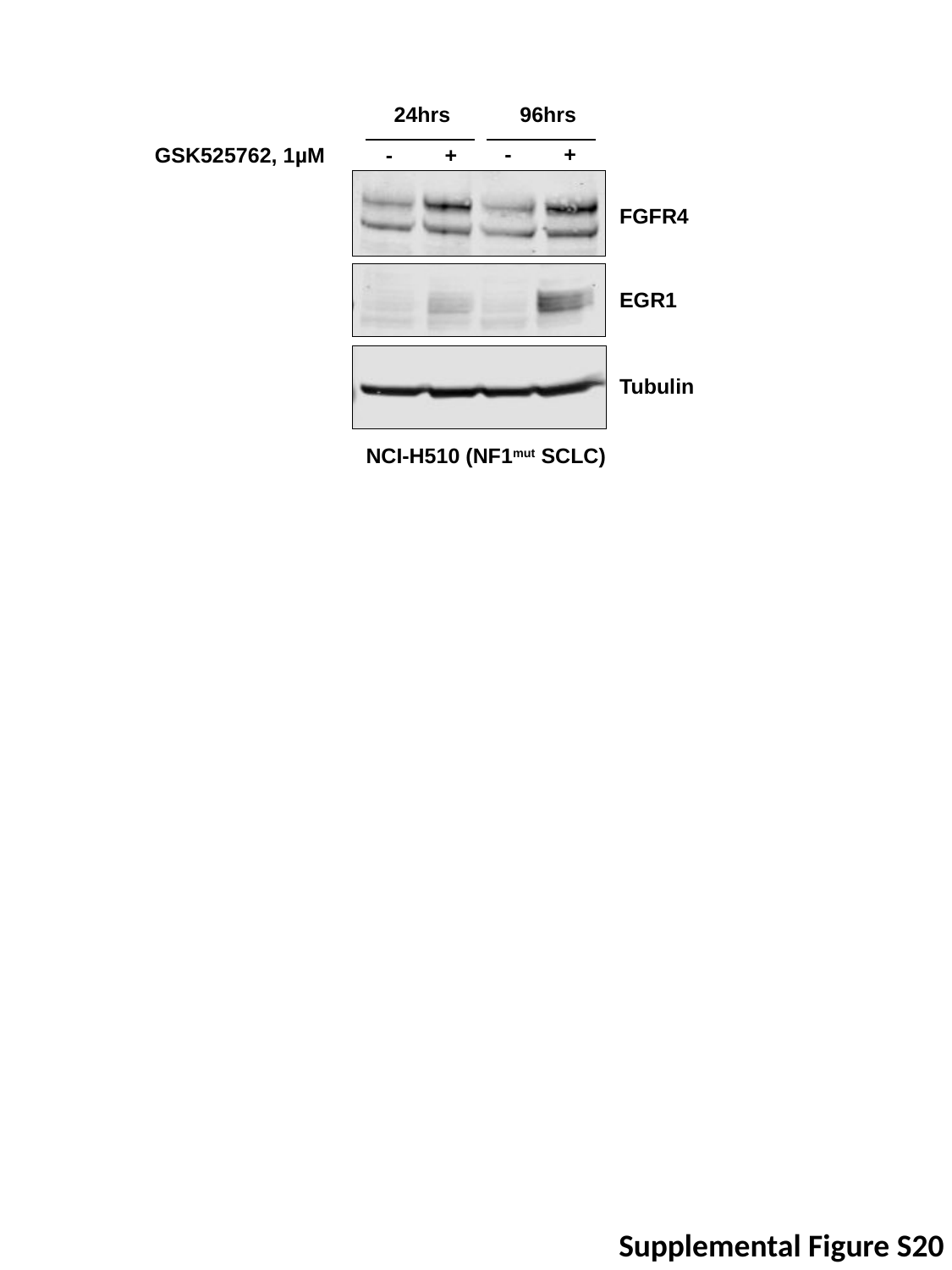

24hrs
96hrs
-
+
GSK525762, 1µM
-
+
FGFR4
EGR1
Tubulin
NCI-H510 (NF1mut SCLC)
Supplemental Figure S20
