# Supplementary material for: MEK inhibitors overcome resistance to BET inhibition across a number of solid and hematologic cancers
Source: Oncogenesis. 2018 Apr 20;7(4):35. doi: 10.1038/s41389-018-0043-9 (PMC5908790; doi:10.1038/s41389-018-0043-9)
Supplement: Supplementary file 4 — Supplemental Table S2 [file 41389_2018_43_MOESM4_ESM.pdf]

Gene	p-value	Number of mutant cell lines	median (gIC50) WT	median (gIC50) MT
TTN	2.03E-05	148	194	909.5
SP1	4.28E-05	120	429	957
SPTA1	7.46E-05	47	601.5	1763
**KRAS**	**0.000118975**	**59**	**550**	**1667**
LPHN3	0.000610149	28	645	2158
DMRT3	0.000610585	13	653.5	3455
ID2	0.000614769	70	550	1226
STK11	0.000875304	33	605	1763
RAG2	0.00107827	8	676	3610.5
OR14K1	0.001183913	18	662	2785.5
XCL1	0.001233386	9	676.75	3455
GABRA2	0.001248074	14	676	3513
CDH23	0.001323427	39	625.5	1665
MED17	0.001354369	5	740.5	49
ADRB1	0.001468471	5	678.75	3976
SGCZ	0.001511119	11	669	3455
WDR17	0.001587777	13	669.75	2363
OR4C15	0.001703178	12	676	2622.5
SDAD1	0.00175536	7	767	124
ANXA8	0.001792847	9	669	3766
NELL1	0.001810239	12	662	3017.5
GTF2H1	0.001817433	5	678.75	3976
SDE2	0.001898776	7	678.75	20000
USP1	0.001964454	75	585	1481
TMEM131	0.002037167	16	662	2269
GPD2	0.002097692	10	676	3405.5
CDK8	0.002342529	6	677.5	3610.5
USP10	0.002405992	4	680	14262
NPR3	0.002503317	7	676.75	2882
LRTM1	0.002616461	5	678.75	3571
TAS2R4	0.002770259	18	662	2363
XKR6	0.002788804	8	677.5	3169.5
TP53	0.002814469	163	391.5	826
DUSP27	0.002820499	19	669	2455
PDIA5	0.002938156	4	680	19594.5
FAM47B	0.003083305	16	676	2759
TAS2R16	0.003479873	6	677.5	6891.5
GOLGA8R	0.003727503	10	780	145
MAP2	0.003839946	57	569	959
TNPO1	0.00385541	6	758	115
DHX32	0.003866607	5	678.75	3766
TK1	0.004151691	51	586.5	1249
MAGEA2	0.004157603	11	676.75	3541
TRIM46	0.004215206	5	678.75	3455
PEX6	0.004268965	7	678.75	3455
EPB41L4A	0.004579312	9	767	187
HOXD1	0.004671426	21	653.5	1955
NTRK3	0.004771297	17	653.5	1763
VSX2	0.004771697	6	677.5	3564.5
ENTPD8	0.004999214	5	678.75	3455
TBX1	0.005048966	25	627.5	1683
CLPTM1	0.005059968	5	678.75	3356

MUC19	0.005336279	33	608	1643
TSTD2	0.005504273	5	740.5	138
F9	0.005528814	94	532	983
CDKL1	0.005530155	4	680	11727.5
CBX4	0.00563753	5	740.5	75
TMEM132C	0.005658266	19	653.5	1117
SAR1A	0.005679578	4	680	11727.5
ZNF485	0.005988177	7	678.75	20000
OR11H1	0.006033049	11	678.75	3455
ABHD16B	0.006110105	7	678.75	3980
ZNF540	0.006200142	5	740.5	75
GRIK2	0.00624603	14	662	2758
TLK2	0.006276508	6	758	140
OR11H12	0.006424766	7	678.75	3455
C8A	0.006434046	61	597.5	907
RPL10L	0.00655466	7	678.75	2882
STIP1	0.006974248	5	678.75	19189
MYH2	0.007058807	21	653.5	1661
ADAMTS12	0.007111741	28	662	1612
RYR2	0.007180087	67	604.5	959
TENM3	0.00721323	27	608	2102
INHBE	0.007294529	6	680	3873
RGPD2	0.007307456	5	678.75	3356
ELAVL2	0.00731122	13	676.75	2455
DMRTA2	0.007312209	7	676.75	3455
RPL10	0.00731844	11	676.75	2363
PDZD7	0.007430681	9	676.75	2633
EMR1	0.007458096	7	767	124
MYSM1	0.007580406	4	680	11727.5
ROR2	0.00761532	11	676.75	3455
LEP	0.007704644	24	662	2125.5
SBNO2	0.007737064	11	676.75	3455
ZNF684	0.007744016	5	740.5	124
EMILIN1	0.007757723	7	676.75	2882
FASTKD5	0.008075145	8	677.5	2526
ST6GAL2	0.008098868	16	676	2105
EGR2	0.008109937	6	677.5	2909
HTR7	0.008177589	9	676.75	2251
IL9	0.008189438	4	680	5298.5
IL9R	0.008189438	4	680	5298.5
ABCB11	0.008306769	11	676.75	2455
FOXC1	0.008637475	6	758	126.5
NYAP2	0.008775819	12	776	218.5
EFCAB4B	0.008827115	10	676	2288.5
ZSCAN18	0.008972486	10	677.5	3347
OR10V1	0.008990994	9	678.75	3976
ABR	0.009095322	81	565.5	959
EYA4	0.009097598	13	669	1324
HLA-DRB5	0.009097598	13	740.5	132
TNN	0.0091008	92	510	909.5
ARHGEF35	0.009225023	9	676.75	2363
FUT5	0.009388503	6	677.5	3008
PODN	0.009421511	10	677.5	2527
PSMB1	0.00942368	7	678.75	3766
ANKRD13B	0.009709452	9	678.75	3455

SV2A	0.009731892	10	676	2582.5
RALY	0.009732413	16	676	2220
ABRA	0.009746636	42	677.5	1080.5
PPAPDC3	0.009783951	4	680	3773.5
JAKMIP1	0.009784495	11	676.75	3455
PRSS1	0.009825559	59	604.5	829
CCDC54	0.010033017	4	680	3498
WNT8B	0.010067997	5	740.5	124
PRM1	0.010216427	9	676.75	2214
OPRM1	0.010216427	9	676.75	2214
EZR	0.010312711	8	680	3405.5
SLC22A16	0.010326751	11	740.5	143
SS18	0.010490004	12	676	2576.5
MRPL23	0.010768424	5	678.75	3976
KIAA1211	0.010802975	32	645	1715
ZNF574	0.01084392	6	758	177.5
C5orf42	0.01093181	23	653.5	2102
ZNF683	0.010949804	4	680	11727.5
TAAR5	0.011225069	4	680	11322
ARHGEF19	0.011364992	4	723	74.5
DMAP1	0.011364992	4	680	3666
NPAP1	0.011421886	21	627.5	1763
ACRBP	0.01176958	5	740.5	124
C9orf41	0.011900358	5	678.75	3455
EXOC7	0.011940292	4	680	6302.5
MTR	0.011976544	36	645	1184
PRAME	0.011991196	49	599.5	1187
OR2T2	0.012131895	14	676	1853
B4GALT5	0.012165786	5	678.75	3455
PHC1	0.012268087	10	677.5	2784
SLC9A9	0.012418397	8	677.5	2386
SLC44A2	0.012522506	7	740.5	202
CADM1	0.012561275	10	776	218.5
ZCCHC18	0.012639788	7	678.75	2982
G3BP1	0.01269563	4	723	92
MAGEA1	0.012719325	25	627.5	1324
NEU4	0.012773348	13	676.75	2175
MROH6	0.012773348	13	676.75	2409
RP11-1220K2.2	0.012777594	22	645	1797
DAD1	0.012861361	12	758	194.5
TPR	0.012878253	158	235	790.5
PAFAH1B1	0.013007969	6	758	174
ATN1	0.013223231	13	676.75	2250
ITIH6	0.013236265	12	676	2086
PLA2G4E	0.013270564	6	758	154.5
CXCL1	0.013270564	6	680	3610.5
TMEM74	0.013270564	6	677.5	3498
LIPT2	0.013330042	4	680	10799
FASTK	0.013600462	25	660.5	1751
DDC	0.013673244	6	677.5	2893
SIX5	0.013867146	7	678.75	2363
IL2RB	0.013992316	4	680	3758.5
MIDN	0.014086678	6	680	2909
PHKB	0.014122222	10	677.5	2548.5
UBE2O	0.014122222	10	776	220

VAV1	0.014175221	5	678.75	2982
KRT75	0.01424056	9	676.75	1861
PTH	0.014307994	17	653.5	1202
C5	0.01435553	180	161	740.5
GLRA3	0.014368394	6	680	3362
NAPSA	0.014386828	7	740.5	202
AIM1	0.014394805	17	789	221
C20orf26	0.014449489	15	676.75	2455
KSR2	0.01448208	17	676.75	2363
ISYNA1	0.014507914	4	723	99.5
MAF	0.014610515	30	676	1820
ZNF114	0.014642883	8	680	3978
C22orf29	0.014653052	7	676.75	1955
NFRKB	0.014787786	7	678.75	2363
CDH2	0.014810064	72	606	1071
COL4A6	0.014887645	16	680	2316.5
OR5T3	0.015013248	14	677.5	1541
TFRC	0.015040193	4	723	92
PNKD	0.015122491	5	678.75	3455
GDI2	0.015221403	4	680	3218.5
SPHKAP	0.015364772	27	669	1680
LY9	0.015393917	6	680	2909
TRIM68	0.015443102	9	678.75	2689
HOXD13	0.015450409	5	678.75	3455
RALYL	0.015478034	13	676.75	2689
SLC27A6	0.015491768	10	677.5	2728
SUGP2	0.015694062	9	669	1915
EIF4B	0.015776619	4	680	4001.5
INSC	0.015917226	12	676	1708
FUT2	0.015948603	9	678.75	2178
GLCCI1	0.016008407	6	680	2410
ULK4	0.016038801	17	676.75	1888
CAPN12	0.016050353	7	678.75	2363
APAF1	0.016222463	8	758	177
SPTBN2	0.016303523	15	778	249
ITIH2	0.016491778	7	678.75	2711
TPK1	0.016491778	7	678.75	3455
SPI1	0.016544575	4	723	91
TMEM41A	0.01674162	4	680	2758
FBXO5	0.01674162	4	723	115
RPL23	0.016806772	6	677.5	2869.5
METAP1	0.016825493	5	678.75	1915
CSMD3	0.016874234	69	600	1265
VGLL3	0.017135777	6	680	3168.5
TPRN	0.017156274	38	662	1138
UGP2	0.017252497	15	669	1915
HFM1	0.017308079	12	677.5	2572
KRTAP5-10	0.017345169	4	680	12252
UGT1A4	0.017368198	5	740.5	85
GIP	0.017376797	24	676	1901.5
OR10AG1	0.017498941	10	680	2163.5
AL390778.1	0.01755055	4	680	3172.5
MCTP2	0.017553428	9	678.75	3205
TRPC1	0.017693343	9	678.75	3205
RUNX1	0.017714595	23	669	1496

ACOX1	0.017721444	7	678.75	3455
GBP4	0.017721444	7	680.5	3455
OR5T2	0.01816351	17	627.5	1461
ZNF516	0.018185108	13	669	2455
CCDC37	0.018262824	9	678.75	3455
ARHGEF5	0.018486335	17	676.75	2251
OR4A16	0.018560451	8	677.5	2572
WDR1	0.01859684	42	645	1180
PCDHGA1	0.018747636	41	608	1352
TTC7A	0.018866952	6	758	168
ADIPOR2	0.018891122	5	678.75	3455
KEAP1	0.018965338	19	676.75	1667
GAP43	0.019048207	6	680	3610.5
KCNIP1	0.019048844	4	680	3498
EML3	0.019200198	7	678.75	2409
TMEM181	0.019200198	7	717	106
GPAA1	0.019230982	6	680	3067.5
EID2	0.019271768	4	680	3022.5
PGBD1	0.019271768	4	723	135
ZSCAN4	0.019289633	5	678.75	3455
GLS	0.019450104	9	678.75	2363
GRIA1	0.019466534	22	676	1575.5
MAF1	0.019491629	5	678.75	3455
DTWD1	0.019496969	4	680	6252
ZNF341	0.019543091	7	678.75	3455
LRRC8E	0.019672771	8	677.5	2159
C1orf50	0.019695471	5	680.5	3455
DR1	0.019787264	52	610	1180
UQCRC1	0.019977499	6	758	229
BCL11B	0.020001085	8	677.5	2517
TDP1	0.020026295	10	680	2909
TTC40	0.020048172	26	606	1195
TTC4	0.020048172	26	606	1195
ORAI1	0.020360155	6	680	2760
MCCC1	0.020529569	5	678.75	3455
SGK3	0.020749188	6	680	2830
GJB5	0.020897053	4	723	128
TRIM51	0.020946123	6	677.5	2288.5
KIAA0408	0.021092016	10	776	177
HRNR	0.021127026	24	677.5	1366.5
MUC13	0.021144686	6	677.5	2095
POTEG	0.02129807	13	669	1763
PDZRN3	0.02129807	13	678.75	1680
TET2	0.021377508	17	740.5	320
NF1	0.021383005	153	451	826
PER2	0.021500924	17	676.75	1481
NELFA	0.021615384	5	678.75	2363
RIOK1	0.021714917	7	767	202
FAAH	0.021843241	12	676	2109
SPAG4	0.021878417	4	680	2909
ABCC12	0.022002104	13	669	1763
OBSL1	0.022051042	16	676	2093.5
KRTAP5-1	0.022063513	5	678.75	4504
TEP1	0.022075798	20	676	2127
TIMM8B	0.022129935	4	723	79

RAC2	0.022162284	6	680	4887.5
ZNF135	0.022253995	8	677.5	2622.5
TRIM50	0.022534596	10	677.5	2409
HMCN2	0.022581097	6	680	3978
GAB4	0.02268009	7	678.75	2689
AK7	0.022772677	14	677.5	2909
AK5	0.022793071	6	680	3717.5
OR5F1	0.022877536	7	678.75	2455
CCDC3	0.022889684	41	660.5	829
NOX4	0.022960109	11	778	202
KCNC4	0.02303415	10	677.5	2803.5
EIF4G2	0.023161325	4	680	11727.5
YTHDF3	0.023161325	4	680	3072
SETD1A	0.023175764	15	789	249
SLC7A1	0.023236036	28	676	959
MT-CO3	0.023236036	28	610	1349.5
SUFU	0.023425603	4	680	11181.5
SMARCA4	0.023487739	27	669	2633
ID1	0.023557186	74	596	1141
CSNK1E	0.023658348	6	677.5	2784
AFTPH	0.023658348	6	680	3171.5
USP17L2	0.023658348	6	680	3743
KDM4E	0.023682328	7	676.75	1827
SYTL4	0.023682328	7	740.5	187
NOP2	0.023692494	4	680	3304
AARS2	0.023696201	5	740.5	124
ZBTB41	0.023740998	15	678.75	2065
ZNF12	0.023832886	12	680	2020
LRRC66	0.023939316	11	678.75	2455
GFI1	0.024101575	6	680	11988
L3MBTL3	0.02410599	11	680.5	1661
TPGS1	0.024181525	5	678.75	3455
EBLN1	0.024234201	4	681	18193
OR2C3	0.024237329	10	676	2159
FTMT	0.024313752	12	680	2013
OR52E2	0.024413584	10	676	2015
SLC17A7	0.024509057	4	680	6250
CACNA1E	0.024572162	31	661.25	1109
DDX25	0.024675443	5	680.5	2363
GPATCH2	0.024769429	10	776	277
TRIM69	0.024786611	4	681	11988
TRIM16L	0.024786611	4	723	143.5
OR2T27	0.024923929	8	677.5	2294
SUSD2	0.024925663	5	740.5	202
LEPR	0.025006247	22	676	1777.5
OR2T3	0.025217617	17	680.5	1763
HES1	0.025349895	4	680	11602.5
CNKSR2	0.025494614	10	677.5	3304
LRFN5	0.02564703	17	676.75	1461
ATP8B4	0.025791566	17	653.5	1809
AHCTF1	0.025813769	16	677.5	2159
CTF1	0.025813769	16	677.5	2159
ATP12A	0.025834057	13	778	235
MTUS2	0.025836069	23	636	1324
DCAF8L2	0.02586007	9	680.5	3766

SLITRK3	0.025861206	20	662	1471
RETSAT	0.025942537	8	723	135.5
C3orf58	0.025942537	8	677.5	2710.5
P2RY6	0.025948639	5	680.5	3455
TCF23	0.025948639	5	678.75	2711
DBP	0.026150523	8	677.5	2159
SLC5A12	0.026209985	5	680.5	2689
ARHGAP12	0.026245782	7	740.5	202
ZSCAN1	0.026252683	26	645	1235
PAF1	0.026366017	11	767	249
ZNF420	0.026473602	5	740.5	187
HDAC9	0.026535376	21	669.75	1109
SMOX	0.026670212	6	677.5	2758
FBXW11	0.026670212	6	723	157
XK	0.026717091	55	625.5	1324
PPP2R3C	0.027007715	5	678.75	3455
TRPS1	0.027211746	24	645	1047
C12orf10	0.027410522	6	758	188
BMP4	0.027410522	6	677.5	1856
GBF1	0.027521286	13	678.75	2152
PGK2	0.027615101	7	678.75	2982
FAIM	0.02764718	8	677.5	2846.5
NRAS	0.027695123	13	740.5	143
NID2	0.027726976	19	653.5	1496
FLNC	0.027807535	33	634	1202
SLC39A1	0.027843523	28	676	1216.5
CYP4A11	0.027849211	7	740.5	249
SLC7A11	0.027849211	7	676.75	1375
RHOBTB3	0.027878856	9	680.5	2363
KIAA0907	0.027913853	6	680	11883
NPHS2	0.028168493	6	680	2672.5
OTOP2	0.028168493	6	680	3513
VIPR1	0.028168493	6	681	11727.5
ABLIM1	0.02832257	7	680.5	3980
SLC25A31	0.028335446	4	723	167
PAICS	0.028335446	4	680	3668.5
SPATA3	0.028441341	55	669	801
PARK2	0.028612415	11	678.75	2178
CFL1	0.028650028	4	680	3169.5
FOLR3	0.028650028	4	680	9920.5
CUL4A	0.028650028	4	680	2859.5
PDPR	0.028709196	15	676.75	1888
GIMAP2	0.028951004	5	680.5	2689
GTF3C4	0.029217137	8	677.5	1991.5
CCDC168	0.029315778	17	678.75	1955
RIMS2	0.029432699	45	653.5	912
MUC12	0.029459568	26	680	1001.5
TMEM151B	0.029471931	6	680	3169.5
PROL1	0.029527874	5	678.75	2035
SLC22A12	0.029679552	8	680	2668.5
PCDHGA4	0.029796144	11	676.75	2102
GABRG1	0.029810509	9	680.5	3541
VAV3	0.029983154	18	680	2269
PCDHGA12	0.030004172	14	677.5	1569
PELI1	0.030268436	4	723	115

PEG10	0.030278637	6	677.5	2641
FAIM3	0.030285962	7	678.75	2982
GLI3	0.030344813	22	676	1455
TMC2	0.030539423	7	680.5	3976
ANKRD36C	0.030640002	24	662	1471
SLC16A2	0.030711324	5	717	124
C1QC	0.030937459	4	680	3715.5
RGS20	0.030937459	4	723	200.5
PCDHGA10	0.030971179	12	677.5	2057
DDX41	0.031013473	5	680.5	3455
EPX	0.031051813	7	740.5	75
PADI2	0.031104242	6	680	2671.5
TOPBP1	0.031161276	9	740.5	117
XIRP2	0.031191154	47	669.75	758
METTL12	0.031276705	4	723	151.5
OTOF	0.031282745	19	625.5	1117
SKP1	0.03131817	5	740.5	202
EPPK1	0.031427988	25	627.5	1324
HTR5A	0.031542499	10	680	2549
LILRA4	0.031571565	7	680.5	2464
DYRK1B	0.031625431	5	680.5	3455
ZNF429	0.031665316	6	680	3330
GAL3ST2	0.031665316	6	680	3717.5
HYAL4	0.031665316	6	758	182
CCDC87	0.031862627	11	767	224
GPR64	0.031986187	10	758	269.5
IQGAP3	0.032042957	16	677.5	1349.5
ASXL3	0.032077285	28	645	1294.5
FAM132B	0.032235048	6	681	3429
RGMB	0.032235048	6	723	204
CEP72	0.032247717	5	680.5	3455
MFN2	0.03229035	11	676.75	2065
TBX18	0.03229035	11	679.25	2065
ZFHX2	0.032479513	18	676	1492.5
PSMB11	0.032523192	6	680	2716.5
ZNF124	0.032562774	5	678.75	2152
FUT11	0.03266579	4	680	3717.5
TPM4	0.03266579	4	680	3590.5
FRAS1	0.032717175	24	677.5	2053.5
USP47	0.032841326	8	677.5	2112
BHLHE41	0.032880465	5	680.5	3976
SLITRK2	0.03291809	20	662	1334.5
TRIM9	0.033175816	7	678.75	2633
ISX	0.033379926	4	680	3717.5
ACTL6B	0.033379926	4	723	203.5
NACA	0.033512634	25	653.5	1680
SLC24A5	0.033523812	5	678.75	2409
NLRP10	0.033526799	18	676	994
JAKMIP3	0.033529304	9	678.75	3205
ADAMTSL4	0.033529304	9	680.5	2464
IGLL5	0.033529304	9	740.5	237
KLK15	0.033581471	10	676	1302.5
CCNB2	0.033741975	4	723	156.5
TMPRSS15	0.033767964	22	677.5	1478.5
AC079354.1	0.033849504	5	680.5	3980

RBMXL3	0.033870217	12	677.5	2109
EAF2	0.034003771	7	717	85
RDH10	0.034020946	9	676.75	1187
EFHC1	0.034107376	4	680	2909
DBN1	0.03413199	8	677.5	2214
SP2	0.034132921	108	599	823.5
PGS1	0.034269069	9	678.75	3455
TMEM175	0.034269069	9	678.75	2455
CEP57	0.03450901	5	740.5	139
UBXN2B	0.03450901	5	740.5	124
C10orf11	0.03484339	28	662	1670.5
CD209	0.034848335	4	680	3850.5
SLTM	0.03492701	8	758	225.5
PDE8B	0.035022737	9	680.5	3455
ITGB6	0.035179463	5	678.75	2152
FBP2	0.035195507	8	680	2083.5
PAX9	0.035217233	6	677.5	2468
ARID5B	0.035254019	13	678	1809
XPC	0.035518837	5	717	140
WDR45	0.035528008	6	680	3072
PID1	0.035602996	4	680	9210.5
SPEG	0.035714449	25	669	1324
NLRP3	0.035804769	23	676.75	821
MAS1L	0.03584112	6	723	163
TMEM176A	0.035892623	11	676.75	1751
VIP	0.035972972	23	678.75	1888
ELL3	0.035985526	4	723	183
TAS2R41	0.036156582	6	681	3072
SESN1	0.036205973	5	717	75
PRSS36	0.03628718	8	680	2909
MS4A6A	0.036366139	11	740.5	128
VPS72	0.036371554	4	680	2363
CDH10	0.036558193	48	604	1271.5
NPAS4	0.036572593	9	676.75	1955
FRMD6	0.036572593	9	678	1888
C7orf10	0.036594396	7	680.5	3455
ZNF449	0.036761106	4	723	169
ARFGEF1	0.03677649	15	778	235
GPR115	0.036782416	13	681.5	2363
DNAJC13	0.03680963	16	676	2003
CCDC74B	0.036892362	7	678.75	2363
KBTBD12	0.036904407	5	740.5	202
MPP3	0.037154207	4	680	3479
C1orf173	0.037224601	32	645	1106
AGBL1	0.037252299	19	669	1809
LRRC53	0.037407582	8	680	2413.5
CNOT1	0.037529928	25	653.5	1643
STAR	0.037600076	40	662	1580.5
TNPO3	0.037614283	5	740.5	187
KNSTRN	0.037741412	10	723	188
TRPV3	0.037769624	6	680	2932
CCDC108	0.037829306	15	680.5	1809
ATF7	0.037935225	21	799.5	447
PALD1	0.038099475	6	680	2955
PTPRJ	0.038099475	6	680	3065.5

CTGF	0.038099475	6	677.5	2671.5
CDH11	0.038152999	36	680	734.5
C7	0.038164326	144	488	717
NR5A2	0.038180505	9	680.5	2711
NCAPH	0.038313795	11	676.75	2152
ZNF716	0.03847322	15	676.75	1763
ARPP21	0.038633062	17	669	1667
SLC8B1	0.038700865	5	680.5	2363
ALAS1	0.038700865	5	680.5	3455
IMPAD1	0.038700865	5	678.75	1955
LRRC55	0.03876259	4	680	4775.5
EVX1	0.03876653	6	680	2671.5
TNFRSF21	0.038781581	10	677.5	2006.5
ACVR1	0.038901651	14	677.5	2000.5
ALMS1	0.039076268	31	608	1294
AGTR1	0.039173806	4	681	11727.5
SEMA6B	0.039443482	6	680	2798.5
EPHA2	0.039575566	11	740.5	138
ZNF853	0.039575566	11	678.75	2464
IQCC	0.039588721	4	681	3706
OR6T1	0.039668255	7	680.5	3205
GLIPR1L1	0.039785704	6	680	2409
MIA2	0.039785704	6	680	3330
SP100	0.039811679	14	680	1917.5
TACR1	0.040130443	6	677.5	2567.5
PRR23C	0.040417569	9	680.5	3455
PDE9A	0.040429749	4	681	11727.5
ZFR	0.040505998	14	677.5	2103
COLEC12	0.040619287	12	677.5	1684
P4HA2	0.040827525	6	680	2932
SMAD7	0.040855913	4	680	10740.5
SLC19A3	0.040954191	5	740.5	132
TMPRSS11E	0.040954191	5	678.75	2102
DTX1	0.040957759	7	676.75	2214
SLC9A3	0.041045985	16	677.5	1418.5
OR52A1	0.041284652	9	678.75	2363
SOCS5	0.041534842	6	680	2527
EZH2	0.041602674	15	778	260
STK11IP	0.041834226	15	676.75	1861
BAZ1B	0.041874914	8	680	2784
OR10X1	0.041948042	7	680.5	3455
ZNF331	0.042121926	5	680.5	3541
SLC1A1	0.042121926	5	740.5	202
ITLN1	0.042252507	6	680	2362
MED15	0.042252507	6	680	2307
GLIS1	0.042282608	7	678.75	2363
RARS	0.042331885	10	677.5	2409
ZNF268	0.042476038	11	678.75	2035
CACNA1B	0.04258888	29	653.5	1667
SHOX2	0.042619428	7	680.5	4504
SLC29A4	0.042748413	11	749.5	163
DUSP7	0.042915969	5	680.5	3455
OLFM3	0.043066145	9	678.75	2363
ATR	0.043277108	95	569	776
RYR3	0.043303759	46	606	933.5

TBX2	0.04342067	20	677.5	1475
MTRR	0.043461005	8	677.5	2678.5
UBE2QL1	0.043493614	4	723	188
GLB1L	0.043619688	24	801	462.5
PPM1J	0.043722628	5	680.5	3976
GP1BA	0.043946926	4	681	3715.5
OR12D3	0.043946926	4	680	3043.5
TOP3A	0.044015565	16	677.5	2108.5
AAR2	0.044109502	8	677.5	1797
WDR7	0.044227826	53	625.5	1194
CRB2	0.044289557	9	676.75	1763
CDH1	0.044325758	137	432.5	798
RGPD4	0.044384762	14	677.5	1540
TGFB2	0.04440422	4	711	74
CYP11B1	0.044436801	8	680	2322
MAGEB6	0.044436801	8	680	1828.5
CADPS2	0.044507551	13	778	352
NKAIN3	0.044865523	4	680	2601
PLEKHM1	0.044912181	9	676.75	1888
DCAF12L1	0.044945015	15	778	260
NOC3L	0.044956603	5	717	129
HLCS	0.045041267	7	678.75	2102
HABP2	0.045082042	12	677.5	1665
FN1	0.045096958	79	608	758
TBX21	0.045228951	6	680	2643
NBPF3	0.045330861	4	680	2623.5
TMEM178B	0.045330861	4	680	2705
C14orf39	0.045795466	5	678.75	2365
CHRNB2	0.045800261	4	680	2270.5
ITGA10	0.046104196	8	677.5	2363
POGK	0.046114174	7	680.5	3455
GCC2	0.046133721	11	676.75	2065
ATL2	0.046190188	12	776	321
DCAF8	0.046215786	24	681	878.5
PJA2	0.046273749	4	723	229
TG	0.046418669	174	351	788.5
INPP4A	0.046425749	11	767	161
SH2B2	0.046458961	10	677.5	1839
PDP1	0.046647476	5	680.5	3455
DDX60	0.046660733	26	610	1349.5
TTC1	0.046699731	46	662	1194.5
NUSAP1	0.046751352	4	681	3405.5
MIP	0.046804818	40	676	1086
ZNF382	0.046824506	9	740.5	202
XKR7	0.046824506	9	678.75	2251
RB1	0.04689513	105	564	758
INS	0.046910137	51	653.5	907
ECHDC1	0.047078459	5	740.5	194
PDZK1	0.047149796	9	678.75	2711
ALDH1A1	0.047233096	4	723	225.5
DGAT1	0.047512788	5	680.5	3766
MGAT5	0.047658645	21	653.5	1461
SLC12A7	0.047698551	10	677.5	1812
TEX11	0.047698551	10	681	3304
ZBTB14	0.047806079	9	680.5	2214

BCAR1	0.047950159	7	678.75	2982
HNMT	0.04795048	5	680.5	3455
ADM2	0.048012756	10	681	2131
GIF	0.048092852	17	678.75	1661
POLR2A	0.048137089	9	740.5	152
ATE1	0.048174317	8	680	2270
FRMD1	0.048174317	8	677.5	2546.5
RBM10	0.048174317	8	677.5	2583
WDR88	0.048209116	4	723	154
CLDN8	0.048210778	11	678.75	2152
SYNJ2	0.048210778	11	678.75	2455
OGFR	0.048242741	22	676	1737
TMBIM4	0.048391555	5	680.5	3455
GOLGA8I	0.048391555	5	740.5	147
PRAMEF2	0.048575549	13	676.75	2152
SPATA31D1	0.048726485	24	662	1673.5
PCSK4	0.048965825	10	680	1787.5
CSMD2	0.049033632	37	678	758
FOXL1	0.049080954	7	680.5	2363
PTGER3	0.049080954	7	678.75	2689
SLCO4C1	0.049080954	7	740.5	128
POTEF	0.049118434	14	680	1602
MYO3B	0.049194938	16	780	316
ZRANB2	0.049202023	4	680	2909
CDC7	0.049325837	39	608	1202
SP4	0.049363266	64	662	1027
TMEM232	0.049462825	7	678.75	2175
GOLIM4	0.049608573	6	723	320
PCDHGC4	0.049704877	4	723	194.5
STEAP4	0.049735277	5	740.5	174
NACAD	0.049951274	12	676	1770.5
ATP13A5	0.049999114	13	789	351

Supplemental Table S2: Genes with protein-changing mutations that significantly correspond to sensitivity or resistance to GSK525762. p-values based on Wilcoxon rank sum test and number of mutant cell lines are indicated. The median gIC50 score for wildtype or mutant cell lines is indicated. See Methods section for further description of the analysis.
