# Supplementary material for: MEK inhibitors overcome resistance to BET inhibition across a number of solid and hematologic cancers
Source: Oncogenesis. 2018 Apr 20;7(4):35. doi: 10.1038/s41389-018-0043-9 (PMC5908790; doi:10.1038/s41389-018-0043-9)
Supplement: Supplementary file 5 — Supplemental Table S3 [file 41389_2018_43_MOESM5_ESM.pdf]

Type	Cell Line	Combination	Fold Change from Single Agent	Synergistic Growth Effect	Mutations (RAS, RAF, NF1)
AML	OCI-AML3	Strong Synergy	6	Growth inhibition	NRAS Q61L
AML	SHI-1	Strong Synergy	8	Growth inhibition, Cytotoxicity	Unknown
AML	SKM-1	Strong Synergy	8	Cytotoxicity	KRAS K117N
AML	MOLM-16	Strong Synergy	5	Growth inhibition, Cytotoxicity	None
AML	UCSD-AML1	Strong Synergy	11	Growth inhibition, Cytotoxicity	Unknown
AML	NOMO-1	Synergy	4	Growth inhibition	KRAS G13D
AML	TF-1	Synergy	3	Growth inhibition, Cytotoxicity	NRAS Q61P
AML	MV-4-11	Synergy	4	Cytotoxicity	None
AML	KASUMI-1	Synergy	3	Growth inhibition	None
AML	SH-2	Additive	< 3	NA	Unknown
AML	MOLM-13	Additive	< 3	NA	NF1 E1720D
AML	UT-7	Additive	< 3	NA	None
AML	GDM-1	Additive	< 3	NA	None
MM	MCCAR	Strong Synergy	7	Growth inhibition	HRAS Q61R
MM	MM1.R	Strong Synergy	7	Cytotoxicity	KRAS G12A
MM	RPMI-8226	Strong Synergy	12	Growth inhibition, Cytotoxicity	KRAS G12A
MM	U266B1	Strong Synergy	6	Growth inhibition	BRAF K601N
MM	ARH-77	Strong Synergy	7	Growth inhibition	None
MM	MOLP-8	Synergy	4	Growth inhibition	NRAS Q61L
MM	OPM-2	Synergy	3	Growth inhibition	None
MM	NCI-H929	Additive	< 3	NA	NRAS G13D
MM	AMO-1	Additive	< 3	NA	KRAS A146T
MM	HuNS-1	Additive	< 3	NA	NRAS D108N
MM	JJN-3	Additive	< 3	NA	NRAS Q61K
MM	KMS-11	Additive	< 3	NA	None
MM	LP-1	Additive	< 3	NA	None
MM	EJM	Additive	< 3	NA	None
MM	KMS-12-BM	Additive	< 3	NA	None
MM	KMS-12-PE	Additive	< 3	NA	None
TNBC	MDA-MB-231	Strong Synergy	6	Growth inhibition	KRAS G13D, BRAF G464V, NF1 T467fs
TNBC	BT-20	Strong Synergy	14	Growth inhibition	None
TNBC	HCC70	Strong Synergy	13	Growth inhibition, Cytotoxicity	None
TNBC	MDA-MB-468	Strong Synergy	5	Growth inhibition	None
TNBC	MDA-MB-157	Synergy	4	Growth inhibition	NF1 SEESLL2751fs
TNBC	HCC1937	Synergy	3	Growth inhibition	None
TNBC	Hs578T	Additive	< 3	NA	HRAS G12D, NF1 G2745R
TNBC	DU4475	Additive	< 3	NA	BRAF V600E
TNBC	MDA-MB-436	Additive	< 3	NA	BRAF GAGA30del
TNBC	MDA-MB-453	Additive	< 3	NA	None
TNBC	BT-549	Additive	< 3	NA	None
TNBC	HCC1143	Additive	< 3	NA	None
TNBC	HCC38	Additive	< 3	NA	None
TNBC	HCC1806	Additive	< 3	NA	None
NSCLC	NCI-H1355	Strong Synergy	7	Growth inhibition	KRAS G13C
NSCLC	NCI-H2087	Strong Synergy	18	Growth inhibition, Cytotoxicity	NRAS Q61K; BRAF L597V
NSCLC	NCI-H358	Strong Synergy	5	Cytotoxicity	KRAS G12C
NSCLC	SW-900	Strong Synergy	8	Growth inhibition, Cytotoxicity	KRAS G12V
NSCLC	NCI-H1666	Strong Synergy	7	Growth inhibition, Cytotoxicity	BRAF G466V
NSCLC	NCI-H1792	Synergy	4	Cytotoxicity	KRAS G12C
NSCLC	NCI-H2009	Synergy	4	Growth inhibition	KRAS G12A
NSCLC	NCI-H460	Synergy	3	Growth inhibition	KRAS Q61H
NSCLC	NCI-H661	Synergy	4	Growth inhibition	None
NSCLC	A-427	Additive	< 3	NA	KRAS G12D
NSCLC	A549	Additive	< 3	NA	KRAS G12S
NSCLC	NCI-H1155	Additive	< 3	NA	KRAS Q61H
NSCLC	NCI-H1299	Additive	< 3	NA	NRAS Q61K
NSCLC	NCI-H520	Additive	< 3	NA	None
CRC	COLO201	Strong Synergy	8	Growth inhibition, Cytotoxicity	BRAF V600E
CRC	RKO	Strong Synergy	9	Growth inhibition, Cytotoxicity	BRAF V600E, NF1 V2205A
CRC	HT29	Strong Synergy	6	Growth inhibition	BRAF V600E, T119S
CRC	SW837	Strong Synergy	10	Growth inhibition	KRAS G12C
CRC	NCIH630	Strong Synergy	10	Growth inhibition	NF1 C324Y
CRC	SNUC2B	Strong Synergy	8	Growth inhibition	KRAS G12D
CRC	SW403	Strong Synergy	6	Growth inhibition	KRAS G12V
CRC	SW480	Strong Synergy	5	Growth inhibition	KRAS G12V
CRC	NCIH747	Strong Synergy	7	Cytotoxicity	KRAS G13D
CRC	CoLo320	Additive	< 3	NA	None
PaCa	BXPC-3	Strong Synergy	13	Growth inhibition, Cytotoxicity	BRAF p.487_492VTAPTP>A
PaCa	HPAF-II	Strong Synergy	7	Growth inhibition, Cytotoxicity	KRAS G12D
PaCa	CAPAN-1	Strong Synergy	5	Growth inhibition	KRAS G12V
PaCa	HPAC	Synergy	4	Growth inhibition	KRAS G12D
PaCa	CAPAN-2	Additive	< 3	NA	KRAS G12V
ER+ BC	ZR-75-1	Additive	< 3	NA	HRAS E162K
ER+ BC	BT-474	Additive	< 3	NA	None
ER+ BC	EFM-19	Additive	< 3	NA	None
ER+ BC	HCC1428	Additive	< 3	NA	None
ER+ BC	KPL-1	Additive	< 3	NA	None
ER+ BC	MCF7	Additive	< 3	NA	None
ER+ BC	T-47D	Additive	< 3	NA	None

Supplemental Table S3: Combination activity for I-BET151 and PD0325901 in cancer cell lines. Combination effects for the indicated cancer cell lines treated with I-BET151 and PD0325901 for 3 or 6 days. Strong synergy, synergy, and additive effects are defined as described in the Methods section. Fold change in potency for the combination compared to the more potent single agent therapy is indicated. Synergistic growth inhibition indicates potency shifts in gIC_{50} or gIC_{100} values, wherease synergistic cytotoxicity indicates potency shifts in dEC_{50} values. Mutations in RAS, RAF, or NF1 genes were extracted from CCLE.
