# Supplementary material for: MEK inhibitors overcome resistance to BET inhibition across a number of solid and hematologic cancers
Source: Oncogenesis. 2018 Apr 20;7(4):35. doi: 10.1038/s41389-018-0043-9 (PMC5908790; doi:10.1038/s41389-018-0043-9)
Supplement: Supplementary file 6 — Supplemental Table S4 [file 41389_2018_43_MOESM6_ESM.pdf]

Top 10 Gene Sets Significantly Overlapping with GSK525762 Down-regulated Genes at 24 hours			
**Gene Set Name**	# Genes in Gene Set	# Genes in	FDR q-value
HALLMARK_E2F_TARGETS	200	26	3.56E-37
HALLMARK_G2M_CHECKPOINT	200	25	1.57E-35
HALLMARK_MITOTIC_SPINDLE	200	14	3.91E-16
HALLMARK_ESTROGEN_RESPONSE_LATE	200	6	0.000075
HALLMARK_GLYCOLYSIS	200	5	0.000732
HALLMARK_MYC_TARGETS_V1	200	5	0.000732
HALLMARK_EPITHELIAL_MESENCHYMAL_TRANSITION	200	4	0.00663
HALLMARK_MTORC1_SIGNALING	200	4	0.00663
HALLMARK_SPERMATOGENESIS	135	3	0.0191
HALLMARK_MYC_TARGETS_V2	58	2	0.0372

Top 10 Gene Sets Significantly Overlapping with GSK525762 Up-regulated Genes at 24 hours			
**Gene Set Name**	# Genes in Gene Set	# Genes in	FDR q-value
HALLMARK_XENOBIOTIC_METABOLISM	200	5	0.00154
HALLMARK_TGF_BETA_SIGNALING	54	3	0.00318

Supplemental Table S4A: Top ten genes sets with q≤ 0.05 from the Broad Molecular Signature Database (MSigDB; http://software.broadinstitute.org/gsea/msigdb/index.jsp) most significantly overlapping with down- or up-regulated genes (q≤ 0.05; log_2FC >1 or <-1) in RKO cells treated with 500nM GSK525762 compared to DMSO for 24 hours. Gene sets from the

Top 10 Gene Sets Significantly Overlapping with GSK525762 Down-regulated Genes at 96 hours			
**Gene Set Name**	# Genes in Gene Set (K)	# Genes in Overlap (k)	FDR q-value
HALLMARK_KRAS_SIGNALING_UP	200	8	0.0000017
HALLMARK_MTORC1_SIGNALING	200	8	0.0000017
HALLMARK_P53_PATHWAY	200	8	0.0000017
HALLMARK_HYPOXIA	200	7	0.0000202
HALLMARK_UNFOLDED_PROTEIN_RESPONSE	113	5	0.000171
HALLMARK_EPITHELIAL_MESENCHYMAL_TRANSITION	200	6	0.000183
HALLMARK_ESTROGEN_RESPONSE_LATE	200	5	0.00141
HALLMARK_GLYCOLYSIS	200	5	0.00141
HALLMARK_IL2_STAT5_SIGNALING	200	5	0.00141
HALLMARK_ESTROGEN_RESPONSE_EARLY	200	4	0.0111

Top 10 Gene Sets Significantly Overlapping with GSK525762 Up-regulated Genes at 96 hours			
**Gene Set Name**	# Genes in Gene Set (K)	# Genes in Overlap (k)	FDR q-value
HALLMARK_TNFA_SIGNALING_VIA_NFKB	200	10	0.000000252
HALLMARK_ESTROGEN_RESPONSE_LATE	200	9	0.00000186
HALLMARK_APOPTOSIS	161	8	0.00000314
HALLMARK_ESTROGEN_RESPONSE_EARLY	200	7	0.0000945
HALLMARK_HEME_METABOLISM	200	7	0.0000945
HALLMARK_P53_PATHWAY	200	7	0.0000945
HALLMARK_COAGULATION	138	6	0.000103
HALLMARK_TGF_BETA_SIGNALING	54	4	0.000324
HALLMARK_XENOBIOTIC_METABOLISM	200	5	0.00542
HALLMARK_UV_RESPONSE_UP	158	4	0.0151

Supplemental Table S4B: Top ten genes sets with q≤ 0.05 from the Broad Molecular Signature Database (MSigDB; http://software.broadinstitute.org/gsea/msigdb/index.jsp) most significantly overlapping with down- or up-regulated genes (q≤ 0.05; log_2FC >1 or <-1) in RKO cells treated with 500nM GSK525762 compared to DMSO for 96 hours. Gene sets from the Hallmark

Top 10 Gene Sets Significantly Overlapping with Trametinib Down-regulated Genes at 24 hours			
**Gene Set Name**	# Genes in Gene Set (K)	# Genes in Overlap (k)	FDR q-value
HALLMARK_TNFA_SIGNALING_VIA_NFKB	200	24	1.19E-22
HALLMARK_E2F_TARGETS	200	18	6.6E-15
HALLMARK_KRAS_SIGNALING_UP	200	17	7.84E-14
HALLMARK_MTORC1_SIGNALING	200	16	9.82E-13
HALLMARK_G2M_CHECKPOINT	200	15	1.22E-11
HALLMARK_HYPOXIA	200	13	1.97E-09
HALLMARK_ESTROGEN_RESPONSE_EARLY	200	12	1.46E-08
HALLMARK_ESTROGEN_RESPONSE_LATE	200	12	1.46E-08
HALLMARK_IL2_STAT5_SIGNALING	200	12	1.46E-08
HALLMARK_P53_PATHWAY	200	12	1.46E-08

Top 10 Gene Sets Significantly Overlapping with Trametinib Up-regulated Genes at 24 hours			
**Gene Set Name**	# Genes in Gene Set (K)	# Genes in Overlap (k)	FDR q-value
HALLMARK_P53_PATHWAY	200	19	1.08E-12
HALLMARK_APOPTOSIS	161	15	4.02E-10
HALLMARK_TNFA_SIGNALING_VIA_NFKB	200	16	5.39E-10
HALLMARK_UV_RESPONSE_DN	144	14	5.39E-10
HALLMARK_EPITHELIAL_MESENCHYMAL_TRANSITION	200	15	3.49E-09
HALLMARK_INTERFERON_ALPHA_RESPONSE	97	11	7.36E-09
HALLMARK_TGF_BETA_SIGNALING	54	9	7.36E-09
HALLMARK_IL2_STAT5_SIGNALING	200	13	0.000000183
HALLMARK_COAGULATION	138	11	0.000000237
HALLMARK_HYPOXIA	200	12	0.00000108

Supplemental Table S4C: Top ten genes sets with q≤ 0.05 from the Broad Molecular Signature Database (MSigDB; http://software.broadinstitute.org/gsea/msigdb/index.jsp) most significantly overlapping with down- or up-regulated genes (q≤ 0.05; log2FC >1 or <-1) in RKO cells treated with 30nM trametinib compared to DMSO for 24 hours. Gene sets

Top 10 Gene Sets Significantly Overlapping with Trametinib Down-regulated Genes at 96 hours			
**Gene Set Name**	# Genes in Gene Set (K)	# Genes in Overlap (k)	FDR q-value
HALLMARK_TNFA_SIGNALING_VIA_NFKB	200	32	4.91E-29
HALLMARK_MTORC1_SIGNALING	200	27	1.14E-22
HALLMARK_HYPOXIA	200	20	1.79E-14
HALLMARK_KRAS_SIGNALING_UP	200	20	1.79E-14
HALLMARK_UNFOLDED_PROTEIN_RESPONSE	113	14	1.39E-11
HALLMARK_GLYCOLYSIS	200	16	2.19E-10
HALLMARK_P53_PATHWAY	200	16	2.19E-10
HALLMARK_APOPTOSIS	161	14	1.09E-09
HALLMARK_ESTROGEN_RESPONSE_EARLY	200	15	1.43E-09
HALLMARK_G2M_CHECKPOINT	200	15	1.43E-09

Top 10 Gene Sets Significantly Overlapping with Trametinib Up-regulated Genes at 96 hours			
**Gene Set Name**	# Genes in Gene Set (K)	# Genes in Overlap (k)	FDR q-value
HALLMARK_INTERFERON_ALPHA_RESPONSE	97	19	3.53E-15
HALLMARK_P53_PATHWAY	200	24	1.85E-14
HALLMARK_UV_RESPONSE_DN	144	18	2.54E-11
HALLMARK_INTERFERON_GAMMA_RESPONSE	200	20	5.94E-11
HALLMARK_TNFA_SIGNALING_VIA_NFKB	200	20	5.94E-11
HALLMARK_TGF_BETA_SIGNALING	54	12	6.24E-11
HALLMARK_ESTROGEN_RESPONSE_LATE	200	19	3.51E-10
HALLMARK_APOPTOSIS	161	16	5.3E-09
HALLMARK_EPITHELIAL_MESENCHYMAL_TRANSITION	200	17	1.29E-08
HALLMARK_HEME_METABOLISM	200	17	1.29E-08

Supplemental Table S4D: Top ten genes sets with q≤ 0.05 from the Broad Molecular Signature Database (MSigDB; http://software.broadinstitute.org/gsea/msigdb/index.jsp) most significantly overlapping with down- or up-regulated genes (q≤ 0.05; log2FC >1 or <-1) in RKO cells treated with 30nM trametinib compared to DMSO for 96 hours. Gene sets

Top 15 Gene Sets Significantly Overlapping with Combination Down-regulated Genes at 24 hours			
**Gene Set Name**	# Genes in Gene Set (K)	# Genes in Overlap (k)	FDR q-value
HALLMARK_E2F_TARGETS	200	80	3.89E-89
HALLMARK_G2M_CHECKPOINT	200	67	1.76E-68
HALLMARK_MTORC1_SIGNALING	200	41	3.15E-32
HALLMARK_MYC_TARGETS_V2	58	24	2.7E-27
HALLMARK_MITOTIC_SPINDLE	200	33	4.59E-23
HALLMARK_TNFA_SIGNALING_VIA_NFKB	200	33	4.59E-23
HALLMARK_MYC_TARGETS_V1	200	28	9.92E-18
HALLMARK_ESTROGEN_RESPONSE_LATE	200	27	9.24E-17
HALLMARK_DNA_REPAIR	150	24	9.51E-17
HALLMARK_ESTROGEN_RESPONSE_EARLY	200	24	6.85E-14
HALLMARK_KRAS_SIGNALING_UP	200	23	5.53E-13
HALLMARK_IL2_STAT5_SIGNALING	200	22	4.28E-12
HALLMARK_GLYCOLYSIS	200	21	3.18E-11
HALLMARK_HYPOXIA	200	20	2.25E-10
HALLMARK_UNFOLDED_PROTEIN_RESPONSE	113	14	9.52E-09

Top 15 Gene Sets Significantly Overlapping with Combination Up-regulated Genes at 24 hours			
**Gene Set Name**	# Genes in Gene Set (K)	# Genes in Overlap (k)	FDR q-value
HALLMARK_APOPTOSIS	161	22	8E-16
HALLMARK_P53_PATHWAY	200	23	3.62E-15
HALLMARK_UV_RESPONSE_DN	144	15	1.95E-09
HALLMARK_TGF_BETA_SIGNALING	54	10	6.05E-09
HALLMARK_ADIPOGENESIS	200	16	1.03E-08
HALLMARK_HEME_METABOLISM	200	16	1.03E-08
HALLMARK_HYPOXIA	200	16	1.03E-08
HALLMARK_COAGULATION	138	13	4.38E-08
HALLMARK_IL2_STAT5_SIGNALING	200	15	5.64E-08
HALLMARK_TNFA_SIGNALING_VIA_NFKB	200	15	5.64E-08
HALLMARK_INTERFERON_ALPHA_RESPONSE	97	11	0.000000066
HALLMARK_EPITHELIAL_MESENCHYMAL_TRANSITION	200	14	0.000000316
HALLMARK_MITOTIC_SPINDLE	200	14	0.000000316
HALLMARK_PROTEIN_SECRETION	96	9	0.0000055
HALLMARK_ESTROGEN_RESPONSE_LATE	200	12	0.0000116

Supplemental Table S4E: Top fifteen genes sets with q≤ 0.05 from the Broad Molecular Signature Database (MSigDB; http://software.broadinstitute.org/gsea/msigdb/index.jsp) most significantly overlapping with down- or up-regulated genes (q≤ 0.05; log2FC >1 or <-1) in combination treated RKO cells compared to DMSO for 24 hours. Gene sets from the Hallmark collection were

Top 15 Gene Sets Significantly Overlapping with Combination Down-regulated Genes at 96 hours			
**Gene Set Name**	# Genes in Gene Set (K)	# Genes in Overlap (k)	FDR q-value
HALLMARK_E2F_TARGETS	200	98	8.74E-104
HALLMARK_G2M_CHECKPOINT	200	84	8.18E-82
HALLMARK_MYC_TARGETS_V1	200	55	7.95E-42
HALLMARK_MTORC1_SIGNALING	200	48	1.36E-33
HALLMARK_MITOTIC_SPINDLE	200	38	8.39E-23
HALLMARK_MYC_TARGETS_V2	58	22	1.39E-20
HALLMARK_ESTROGEN_RESPONSE_LATE	200	34	5.54E-19
HALLMARK_TNFA_SIGNALING_VIA_NFKB	200	28	1.45E-13
HALLMARK_DNA_REPAIR	150	23	3.77E-12
HALLMARK_UNFOLDED_PROTEIN_RESPONSE	113	20	6.83E-12
HALLMARK_GLYCOLYSIS	200	24	2.09E-10
HALLMARK_KRAS_SIGNALING_UP	200	23	1.05E-09
HALLMARK_OXIDATIVE_PHOSPHORYLATION	200	23	1.05E-09
HALLMARK_SPERMATOGENESIS	135	18	7.78E-09
HALLMARK_UV_RESPONSE_UP	158	19	1.52E-08

Top 15 Gene Sets Significantly Overlapping with Combination Up-regulated Genes at 96 hours			
**Gene Set Name**	# Genes in Gene Set (K)	# Genes in Overlap (k)	FDR q-value
HALLMARK_P53_PATHWAY	200	44	6.45E-23
HALLMARK_MITOTIC_SPINDLE	200	40	1.71E-19
HALLMARK_HEME_METABOLISM	200	34	1.69E-14
HALLMARK_APOPTOSIS	161	30	4.7E-14
HALLMARK_UV_RESPONSE_DN	144	27	7.53E-13
HALLMARK_TNFA_SIGNALING_VIA_NFKB	200	31	2.03E-12
HALLMARK_HYPOXIA	200	30	1.01E-11
HALLMARK_EPITHELIAL_MESENCHYMAL_TRANSITION	200	28	2.09E-10
HALLMARK_IL2_STAT5_SIGNALING	200	28	2.09E-10
HALLMARK_MYOGENESIS	200	28	2.09E-10
HALLMARK_TGF_BETA_SIGNALING	54	15	3.23E-10
HALLMARK_ESTROGEN_RESPONSE_EARLY	200	27	8.92E-10
HALLMARK_WNT_BETA_CATENIN_SIGNALING	42	13	1.13E-09
HALLMARK_APICAL_JUNCTION	200	26	3.51E-09
HALLMARK_GLYCOLYSIS	200	26	3.51E-09

Supplemental Table S4F: Top fifteen genes sets with q≤ 0.05 from the Broad Molecular Signature Database (MSigDB; http://software.broadinstitute.org/gsea/msigdb/index.jsp) most significantly overlapping with down- or up-regulated genes (q≤ 0.05; log2FC >1 or <-1) in combination treated RKO cells compared to DMSO for 96 hours. Gene sets from the Hallmark collection were analyzed.
