# Supplementary material for: MEK inhibitors overcome resistance to BET inhibition across a number of solid and hematologic cancers
Source: Oncogenesis. 2018 Apr 20;7(4):35. doi: 10.1038/s41389-018-0043-9 (PMC5908790; doi:10.1038/s41389-018-0043-9)
Supplement: Supplementary file 7 — Supplemental Table S5 [file 41389_2018_43_MOESM7_ESM.pdf]

Top 10 Gene Sets Significantly Overlapping with Down-regulated Genes Specific to Combination in RKO Cells			
**Gene Set Name**	**# Genes in Gene Set (K)**	**# Genes in Overlap (k)**	**FDR q-value**
REACTOME_DNA_REPLICATION	192	11	9.9E-12
REACTOME_CELL_CYCLE_MITOTIC	395	12	5.12E-11
REACTOME_CELL_CYCLE	421	12	7.16E-10
REACTOME_DNA_STRAND_ELONGATION	30	6	1.59E-09
REACTOME_S_PHASE	109	8	1.59E-09
KEGG_DNA_REPLICATION	36	6	4.11E-09
HALLMARK_G2M_CHECKPOINT	200	9	4.13E-09
BMI1_DN_MEL18_DN.V1_UP	145	8	9.98E-09
REACTOME_SYNTHESIS_OF_DNA	92	7	1.56E-08
REACTOME_MITOTIC_M_M_G1_PHASES	172	8	3.13E-08

Top 10 Gene Sets Significantly Overlapping with Up-regulated Genes Specific to Combination in RKO Cells			
**Gene Set Name**	**# Genes in Gene Set (K)**	**# Genes in Overlap (k)**	**FDR q-value**
REACTOME_RNA_POL_I_PROMOTER_OPENING	62	20	2.71E-45
KEGG_SYSTEMIC_LUPUS_ERYTHEMATOSUS	140	22	6.59E-43
REACTOME_AMYLOIDS	83	20	7.94E-43
REACTOME_MEIOTIC_RECOMBINATION	86	20	1.33E-42
REACTOME_RNA_POL_I_TRANSCRIPTION	89	20	2.31E-42
REACTOME_MEIOSIS	116	20	6.74E-40
REACTOME_RNA_POL_I_RNA_POL_III_AND_MITOCHONDRIAL_TRANSCRIPTION	122	20	1.73E-39
REACTOME_TRANSCRIPTION	210	20	1.55E-34
REACTOME_PACKAGING_OF_TELOMERE_ENDS	48	10	8.16E-20
REACTOME_DEPOSITION_OF_NEW_CENPA_CONTAINING_NUCLEOSOMES_AT_THE_CENTROMERE	64	10	1.69E-18

Supplemental Table S5: Top ten genes sets from the Broad Molecular Signature Database (MSigDB; http://software.broadinstitute.org/gsea/msigdb/index.jsp) most significantly overlapping with down- or up-regulated genes in RKO cells specifically observed with combination treatment at 24 hours. Gene sets from the following collections were included in the analysis: C6, CP, CP:BIOCARTA, CP:KEGG, CP:REACTOME, H.
