# Supplementary material for: MEK inhibitors overcome resistance to BET inhibition across a number of solid and hematologic cancers
Source: Oncogenesis. 2018 Apr 20;7(4):35. doi: 10.1038/s41389-018-0043-9 (PMC5908790; doi:10.1038/s41389-018-0043-9)
Supplement: Supplementary file 8 — Supplemental Table S6 [file 41389_2018_43_MOESM8_ESM.pdf]

**Supplemental Table S6:** Top ten genes sets from the Broad Molecular Signature Database (MSigDB; http://software.broadinstitute.org/gsea/msigdb/index.jsp) most significantly overlapping with down- or up-regulated genes in RKO cells specifically observed with combination treatment at 96 hours. Gene sets from the following collections were included in the analysis: C6, CP, CP:BIOCARTA, CP:KEGG, CP:REACTOME, H.

| Top 10 Gene Sets Significantly Overlapping with Down-regulated Genes Specific to Combination in RKO Cells | | | |
|---|---|---|---|
| **Gene Set Name** | # Genes in Gene Set (K) | # Genes in Overlap (k) | FDR q-value |
| HALLMARK_KRAS_SIGNALING_UP | 200 | 10 | 1.98E-09 |
| RB_P107_DN.V1_UP | 140 | 8 | 6.75E-08 |
| HALLMARK_INFLAMMATORY_RESPONSE | 200 | 8 | 5.76E-07 |
| HALLMARK_TNFA_SIGNALING_VIA_NFKB | 200 | 8 | 5.76E-07 |
| REACTOME_CELL_CYCLE_MITOTIC | 325 | 9 | 1.04E-06 |
| NABA_MATRISOME | 1028 | 13 | 2.99E-06 |
| REACTOME_CELL_CYCLE | 421 | 9 | 4.59E-06 |
| REACTOME_DNA_REPLICATION | 192 | 7 | 4.59E-06 |
| REACTOME_G1_S_TRANSITION | 112 | 6 | 4.59E-06 |
| EGFR_UP.V1_UP | 193 | 7 | 4.59E-06 |

| Top 10 Gene Sets Significantly Overlapping with Up-regulated Genes Specific to Combination in RKO Cells | | | |
|---|---|---|---|
| **Gene Set Name** | # Genes in Gene Set (K) | # Genes in Overlap (k) | FDR q-value |
| STK33_SKM_UP | 290 | 11 | 2.46E-11 |
| KEGG_SYSTEMIC_LUPUS_ERYTHEMATOSUS | 140 | 8 | 2.29E-09 |
| STK33_UP | 293 | 7 | 1.65E-05 |
| EGFR_UP.V1_DN | 196 | 6 | 2.97E-05 |
| PTEN_DN.V1_UP | 191 | 5 | 4.76E-04 |
| MTOR_UP.N4.V1_DN | 193 | 5 | 4.76E-04 |
| LTE2_UP.V1_DN | 196 | 5 | 4.76E-04 |
| MEK_UP.V1_UP | 196 | 5 | 4.76E-04 |
| CSR_LATE_UP.V1_DN | 170 | 4 | 6.35E-03 |
| HALLMARK_COMPLEMENT | 200 | 4 | 9.72E-03 |
