# Supplementary material for: MEK inhibitors overcome resistance to BET inhibition across a number of solid and hematologic cancers
Source: Oncogenesis. 2018 Apr 20;7(4):35. doi: 10.1038/s41389-018-0043-9 (PMC5908790; doi:10.1038/s41389-018-0043-9)
Supplement: Supplementary file 9 — Supplemental Table S7 [file 41389_2018_43_MOESM9_ESM.pdf]

**96 hours**		**RKO**			**COLO 201**			**BxPC-3**			**HPAF-II**	
**Signature**	**BET vs. DMSO**	**MEK vs. DMSO**	**Combo vs. DMSO**	**BET vs. DMSO**	**MEK vs. DMSO**	**Combo vs. DMSO**	**BET vs. DMSO**	**MEK vs. DMSO**	**Combo vs. DMSO**	**BET vs. DMSO**	**MEK vs. DMSO**	**Combo vs. DMSO**
REACTOME_CELL_CYCLE_MITOTIC	0.1623	0.1279	0.0000	ND	0.0453	0.0273	0.0277	1.26E-05	0.0000	7.52E-06	0.0000	0.0000
HALLMARK_G2M_CHECKPOINT	0.0336	0.0722	0.0000	ND	0.0998	0.0777	0.0103	2.39E-06	0.0000	0.0000	0.0000	0.0000
REACTOME_CELL_CYCLE	ND	0.2170	0.0001	ND	0.0878	0.0601	0.1496	1.27E-05	1.03E-05	2.30E-04	0.0000	0.0000
REACTOME_DNA_REPLICATION	0.0956	0.0129	0.0000	ND	0.0005	0.0003	0.0037	0.0000	0.0000	0.0000	0.0000	0.0000
REACTOME_MITOTIC_M_M_G1_PHASES	0.0661	0.0252	0.0000	ND	0.0010	0.0007	0.0088	0.0000	0.0000	1.35E-05	0.0000	0.0000
REACTOME_MITOTIC_PROMETAPHASE	0.0763	0.0604	0.0000	ND	0.0780	0.0645	0.0037	7.25E-05	0.0000	0.0000	0.0000	0.0000

Supplemental Table S7: FDR values from Gene Set Enrichment Analysis (GSEA) in the indicated cell lines following 96 hour exposure to GSK525762 (500nM) and/or trametinib (3-30 nM) compared to DMSO treatment. All FDR values shown indicate down-regulation of the signature in treated samples compared to control. ND indicates that the signature was not down-regulated
