# Supplementary material for: MEK inhibitors overcome resistance to BET inhibition across a number of solid and hematologic cancers
Source: Oncogenesis. 2018 Apr 20;7(4):35. doi: 10.1038/s41389-018-0043-9 (PMC5908790; doi:10.1038/s41389-018-0043-9)
Supplement: Supplementary file 10 — Supplemental Table S8 [file 41389_2018_43_MOESM10_ESM.pdf]

	24hrs		96 hrs	
**Gene**	**FDR q value**	**log_2fold change**	**FDR q value**	**log_2fold change**
DUSP6	0.020	1.20	0.002	1.77
SPRED1	0.923	0.04	0.060	0.52
SPRED2	0.741	-0.11	0.769	0.10
EGR1	0.001	1.39	2.62E-06	2.93
ETV5	0.005	1.22	2.01E-04	1.83
SPRY4	0.398	0.21	0.012	0.59
SPRY2	0.197	0.30	6.51E-05	1.24
FGFR1	0.782	0.06	0.397	0.15
FGFR2	0.003	0.50	0.005	0.46
FGFR3	0.039	0.75	0.276	0.41
FGFR4	0.011	0.79	0.016	0.72
EGFR	0.235	0.41	0.212	0.43
ERBB2	0.530	0.12	0.764	0.07
ERBB3	0.506	-0.13	2.60E-04	-0.80
ERBB4	0.778	-0.07	0.494	-0.15
IGF1R	0.177	0.25	0.384	-0.17
FOS	0.051	1.14	0.001	2.13
JUN	0.154	0.41	0.069	0.51
DUSP1	0.128	0.43	0.372	0.27
JUND	0.018	0.40	0.054	0.31
SESN3	2.37E-05	1.45	1.57E-06	1.90
ELL2	0.003	0.92	9.17E-05	1.46
ATF3	0.039	0.77	0.002	1.27
GADD45A	0.007	1.10	0.003	1.21

Supplemental Table S8: FDR and log_2fold change values for the indicated genes in NCI-H510 cells treated with 1uM GSK525762 for 24 or 96 hours.
