# Supplementary material for: MEK inhibitors overcome resistance to BET inhibition across a number of solid and hematologic cancers
Source: Oncogenesis. 2018 Apr 20;7(4):35. doi: 10.1038/s41389-018-0043-9 (PMC5908790; doi:10.1038/s41389-018-0043-9)
Supplement: Supplementary file 11 — Supplemental Figure Legends [file 41389_2018_43_MOESM11_ESM.docx]

**Supplemental Figure S1:** Gene mutations predict sensitivity or resistance to GSK525762 treatment. Bar charts depicting correspondence between the indicated gene mutations (top 10 most significant based on p-value, along with KRAS in Figure 1C) and sensitivity to GSK525762 (gIC_50_, nM).

**Supplemental Figure S2:** KRAS^G12^ missense mutations significantly predict resistance to GSK525762. Bar charts depicting correspondence between KRAS^G12^ mutations and sensitivity to GSK525762 (gIC_50_, nM). p-value from Wilcoxon rank sum test is indicated.

**Supplemental Figure S3:** Anti-proliferative activity of I-BET151 is highly correlated to GSK525762. Growth IC_50_ values for I-BET151 (x-axis) and GSK525762 (y-axis) in a panel of cancer cell lines treated with compound for 6 days. 1:1 correlation is indicated by the solid black line. P-value is based on a two-tailed Pearson correlation.

**Supplemental Figure S4:** Synergistic effects for BET/MEK combinations in cancer cell line models. **(A)** Combination indices (CI) for the combination of I-BET151 with the indicated MEK inhibitors in RPMI-8226 (MM), BxPC-3 (PaCa), and RKO (CRC) cell lines. CI values were calculated from the dEC_50_ values obtained following single agent or combination treatment for 6 days. Bars indicate mean CI +/- SEM (n≥ 3). **(B)** Heat map of cancer cell line response to a combination of I-BET151 with the indicated MEK inhibitors in a 3 or 6 day proliferation assay. Dark orange, light orange, and white bars reflect strong synergy, synergy, and additive effects, respectively (see Materials and Methods). Black circles indicate cell lines possessing mutations in RAS, BRAF, or NF1 genes based on CCLE data. **(C)** X-Y plot indicating the average dEC_50_ of the indicated cell lines at day 6 with GSK525762 treated as a single agent (x-axis) or in combination with various fixed concentrations of trametinib (y-axis). N=2 for RPMI-8226 and BxPC-3. N=3 for RKO.

**Supplemental Figure S5:** Individual tumor growth curves for mice bearing RKO xenografts treated with Vehicle, GSK525762 (15 mg/kg, po, qd), trametinib (1 mg/kg, po, qd), or a combination of the two agents (GSK525762, 15 mg/kg + trametinib, 1 mg/kg; po, qd) for the duration of the study. Individual mice were sacrificed upon reaching an end point tumor volume of 2000 mm^3^ (indicated by red line).

**Supplemental Figure S6:** Individual tumor growth curves for mice bearing MDA-MB-231 xenografts treated with Vehicle, GSK525762 (15 mg/kg, po, qd), trametinib (1 mg/kg, po, qd), or a combination of the two agents (GSK525762, 15 mg/kg + trametinib, 1 mg/kg; po, qd) for the duration of the study. Individual mice were sacrificed upon reaching an end point tumor volume of 1500 mm^3^ (indicated by red line).

**Supplemental Figure S7:** Individual tumor growth curves for mice bearing RPMI-8226 xenografts treated with Vehicle, GSK525762 (25 mg/kg, po, qd), trametinib (0.1 mg/kg, po, qd), or a combination of the two agents (GSK525762, 25 mg/kg + trametinib, 0.1 mg/kg; po, qd) for the duration of the study. Individual mice were sacrificed upon reaching an end point tumor volume of 2000 mm^3^ (indicated by red line).

**Supplemental Figure S8:** Efficacy of BET/MEK combination in HPAF-II xenografts. Mean tumor volume +/- SEM for mice treated with vehicle, 25 mg/kg qd GSK525762, 1 mg/kg qd trametinib, or a combination of the two therapies for 21 days. Single asterisk indicates statistically significant tumor growth inhibition compared to vehicle treated animals on day 18 based on Mann-Whitney test (p=0.01 for GSK525762, p=0.003 for trametinib, p= 0.0002 for combination). Double asterisk indicates statistically significant tumor growth inhibition for combination group compared to single agent groups on day 18. ΔTGI from single agent therapy and p-values are indicated in the table.

**Supplemental Figure S9:** Body weight changes associated with single agent and combination treatments across four xenograft studies detailed in Figure 2D and Supplemental Figure S2. Average body weight ± SD is indicated for vehicle, GSK525762, trametinib, and combination-treated groups.

**Supplemental Figure S10:** Activity for BET/MEK combinations in SCLC. **(A)** Representative curves for the combination of GSK525762 + trametinib in the indicated cell lines plated in a 4:1 fixed ratio (BET:MEK) following 6 days compound exposure. Curves are representative of data obtained from 2 independent biological replicates. For the combination curves, the x-axis represents the concentration of trametinib within the combination. **(B)** Western analysis of cleaved PARP in the indicated SCLC cell lines following 3 day treatment with GSK525762, PD0325901, or a combination of the two agents. **(C)** Response summary for 6 SCLC patient derived xenograft models treated *ex vivo* with a combination of GSK525762 and PD0325901 in a tumor colony formation assay (n=2). **(D)** Representative data for the LXFS-650 patient derived xenograft described in D. **(E)** p-ERK1/2 levels in the indicated SCLC cell lines as determined by capillary-based Western analysis. Data represent peak area for p-ERK1/2 normalized to total ERK1/2.

**Supplemental Figure S11:** Down-regulation of mitotic gene signatures by BET/MEK combination. **(A)** Volcano plots depicting differentially expressed genes at 24 hours in GSK525762 (BET), trametinib (MEK), or combination treated RKO cells. Blue circles highlight expression changes with q< 0.05 and log_2_FC <-1 or >1. **(B)** Volcano plots depicting differentially expressed genes at 96 hours in RKO cells as described in A. **(C)** Venn diagrams indicating overlap of up- (top) and down-regulated (bottom) genes between single agent and combination treatments in RKO cells at 24 hours. Differentially expressed genes were defined by comparison of each treatment to time point matched DMSO controls (q≤ 0.05; log_2_FC ≤-1 or ≥1). **(D)** Venn diagrams indicating overlap of up- (top) and down-regulated (bottom) genes between single agent and combination treatments in RKO cells at 96 hours, as described in C. **(E)** Down-regulation of mitotic gene signatures by BET/MEK combinations across cancer cell lines. Heat map depicting gene set enrichment in the indicated cell lines following 96 hour exposure to a combination of GSK525762 (500nM) and trametinib (3 nM for COLO 201; 10 nM for BxPC-3 and HPAF-II; 30 nM for RKO) compared to DMSO. Heatmap is color coded based on signed (1-FDR) derived from individual GSEA analyses, where a negative value indicates down-regulation in combination treated samples and a positive value indicates up-regulation.

**Supplemental Figure S12:** Down-regulation of mitotic effectors in RKO cells treated with a combination of GSK525762 and trametinib. Western analysis of BUB1B, TTK, and PBK in RKO cells treated with the indicated compounds for 1 or 6 days.

**Supplemental Figure S13:** Down-regulation of mitotic genes by BET/MEK combination in RKO xenografts. qPCR analysis of the indicated genes in RKO xenograft tumors collected at the end point of the efficacy study (2000mm^3^ or day 61). Gene specific data was normalized to GUSB expression, and is presented as average relative expression compared to vehicle controls. Symbols represent individual mice, with mean and standard error indicated with horizontal bars. Asterisk indicates p ≤0.05 for comparison to vehicle group (based on Mann-Whitney test).

**Supplemental Figure S14:** GSK525762 reverses adaptive ERK re-activation in RKO cells following treatment with trametinib. Western blot of phosphorylated (p-ERK1/2) and total ERK1/2 in RKO cells treated with a titration of trametinib (1, 3, 10, 30, 100 nM), alone or in combination with 1 µM GSK525762, for the indicated time points.

**Supplemental Figure S15:** Trametinib as a single agent and in combination with GSK525762 reduces p-ERK1/2 levels in RKO xenografts at early time points. **(A)** H-scores for immunohistochemical staining with p-ERK1/2, ERK1, or ERK2 antibodies in RKO xenograft tumors treated as indicated for 7 days. **(B)** Representative images from RKO xenografts treated as indicated for 7 days following immunohistochemistry with antibodies for p-ERK1/2 or total ERK1.

**Supplemental Figure S16: (A)** Combination BET/MEK treatment results in sustained p-ERK1/2 down-regulation in RKO xenografts. H-scores for immunohistochemical staining with p-ERK1/2, ERK1, or ERK2 antibodies in RKO xenograft tumors treated as indicated. Tumors were harvested as mice reached the endpoint of the efficacy study (2000mm^3^ or day 61). **(B)** Representative images from RKO mouse xenograft samples treated with vehicle, GSK525762, trametinib, or a combination of the two agents following immunohistochemistry with antibodies for p-ERK1/2 or total ERK1.

**Supplemental Figure S17:** Minimal potency shifts over time for p-ERK1/2 inhibition in HPAF-II cells treated with trametinib. Western blot of p-ERK1/2 and total ERK1/2 in HPAF-II cells treated with a titration of trametinib, alone or in combination with 2 µM GSK525762, for the indicated time points.

**Supplemental Figure S18:** Up-regulation of p-ERK1/2 is observed with chemically distinct BET inhibitors. Western analysis of p-ERK1/2 levels in the MM cell line RPMI-8226 following 4 days exposure to the indicated concentrations of I-BET151 or JQ-1.

**Supplemental Figure S19:** BET inhibition up-regulates p-ERK1/2 in a subset of cancer cell lines. **(A)** qPCR analysis of the indicated genes following 24 or 96 hours treatment with GSK525762 in the SCLC cell line NCI-H510 (1 µM). Gene specific data was normalized to GAPDH expression, and is presented as average relative expression compared to DMSO controls (n=2). Standard deviation is indicated. **(B)** qPCR analysis of the indicated genes following 96 hours treatment with 500 nM GSK525762 in the MM cell line RPMI-8226 (n=2), the CRC cell line RKO (n=3), and the PaCa cell line HPAF-II (n=2). Gene specific data was normalized to GAPDH expression, and is presented as average relative expression compared to DMSO controls. Standard deviation is indicated. Asterisks indicate p ≤0.05 for comparison to DMSO controls (two-tailed paired Student’s t-test). Statistical analyses were only performed on data with n ≥ 3.

**Supplemental Figure S20:** BET inhibition up-regulates FGFR4 and EGR1 in the NCI-H510 SCLC cell line. Western analysis of FGFR4 and EGR1 levels in NCI-H510 cells following 1 or 4 days exposure to 1 µM GSK525762.
